# Synthesis of C2-tetrasubstituted indolin-3-ones *via* Cu-catalyzed oxidative dimerization of 2-aryl indoles and cross-addition with indoles†

**DOI:** 10.1039/c9ra04741g

**Published:** 2019-08-02

**Authors:** Anoop Singh, Satheeshvarma Vanaparthi, Sachin Choudhary, Rangan Krishnan, Indresh Kumar

**Affiliations:** Department of Chemistry, Birla Institute of Technology and Science Pilani 333 031 Rajasthan India indresh.chemistry@gmail.com indresh.kumar@pilani.bits-pilani.ac.in; Department of Chemistry, BITS Pilani Hyderabad Campus Secunderabad India

## Abstract

An efficient protocol for the synthesis of 2,2-disubstituted indolin-3-ones under mild conditions has been developed. This reaction involves the copper-catalyzed *in situ* oxidative de-aromatization of 2-arylindoles to indol-3-one, followed by self-dimerization as well as cross-addition with indoles under mild conditions. The result generates a wide variety of C2-tetrasubstituted indolin-3-ones with good to high yields (62–82%).

## Abstract 2

A general protocol for the synthesis of 2,2-disubstituted indolin-3-ones has been developed through self-dimerization of 2-aryl indoles and cross-addition with indoles under mild Cu-catalyzed oxidative conditions with good to high yields.

## Graphical abstract

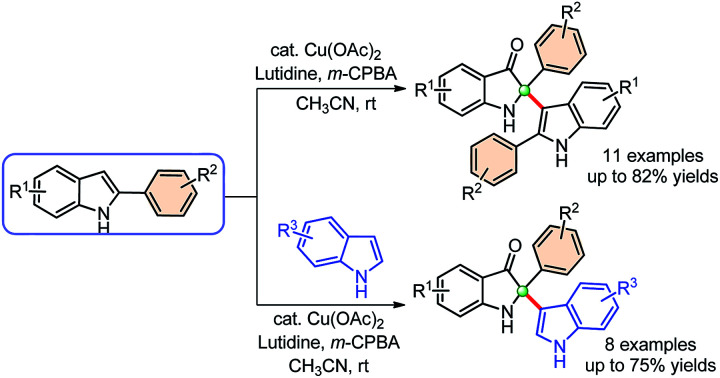

## Introduction

**Fig. 1:**
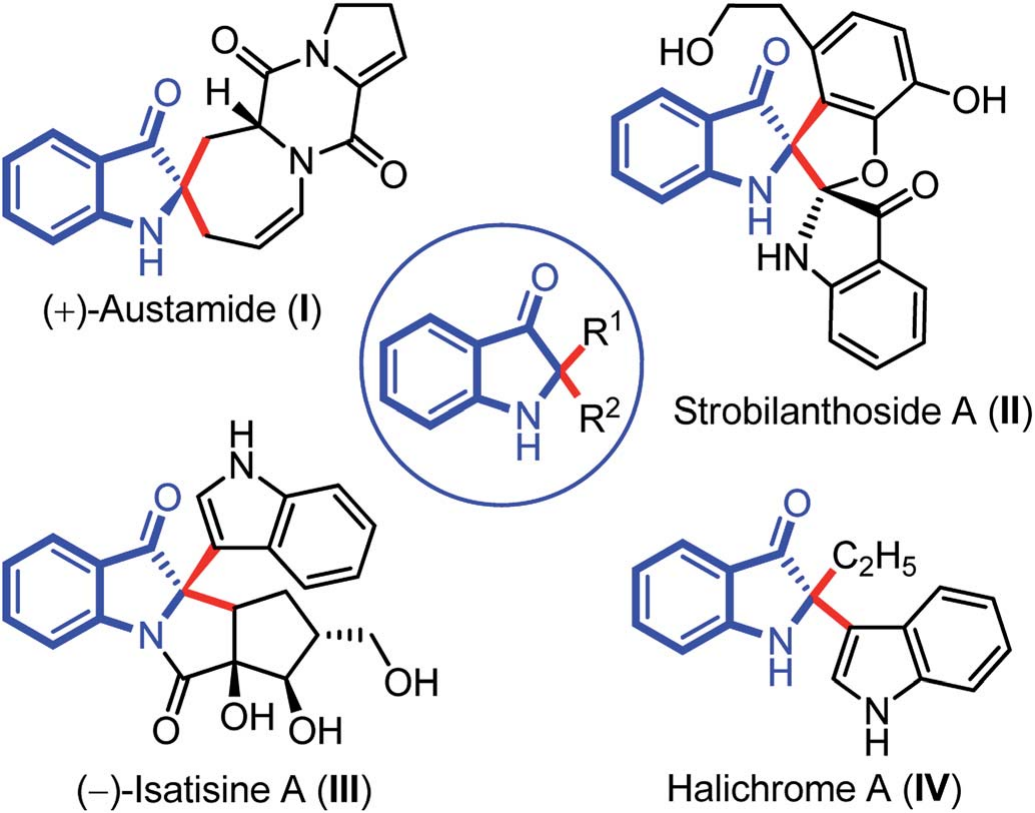
2,2-disubstituted indolin-3-one as basic core in important natural products.

Indolin-3-ones are privileged scaffolds that function as intermediates for the synthesis of medicinally important compounds.^1^ In particular, 2,2-disubstituted 1,2-dihydro-3*H*-indol-3-one, also known as pseudoindoxyls bearing C2 stereocenters have continually appeared in natural products such as austamide (I), strobilanthoside A (II), isatisine A (III), and halichrome A (IV), as well as in many other bioactive synthetic compounds (Fig. 1).^2^ Moreover, compounds with this skeleton have also exhibited exciting applications in the areas of fluorescence labeling and optoelectronic materials in recent years.^3^

**Scheme 1:**
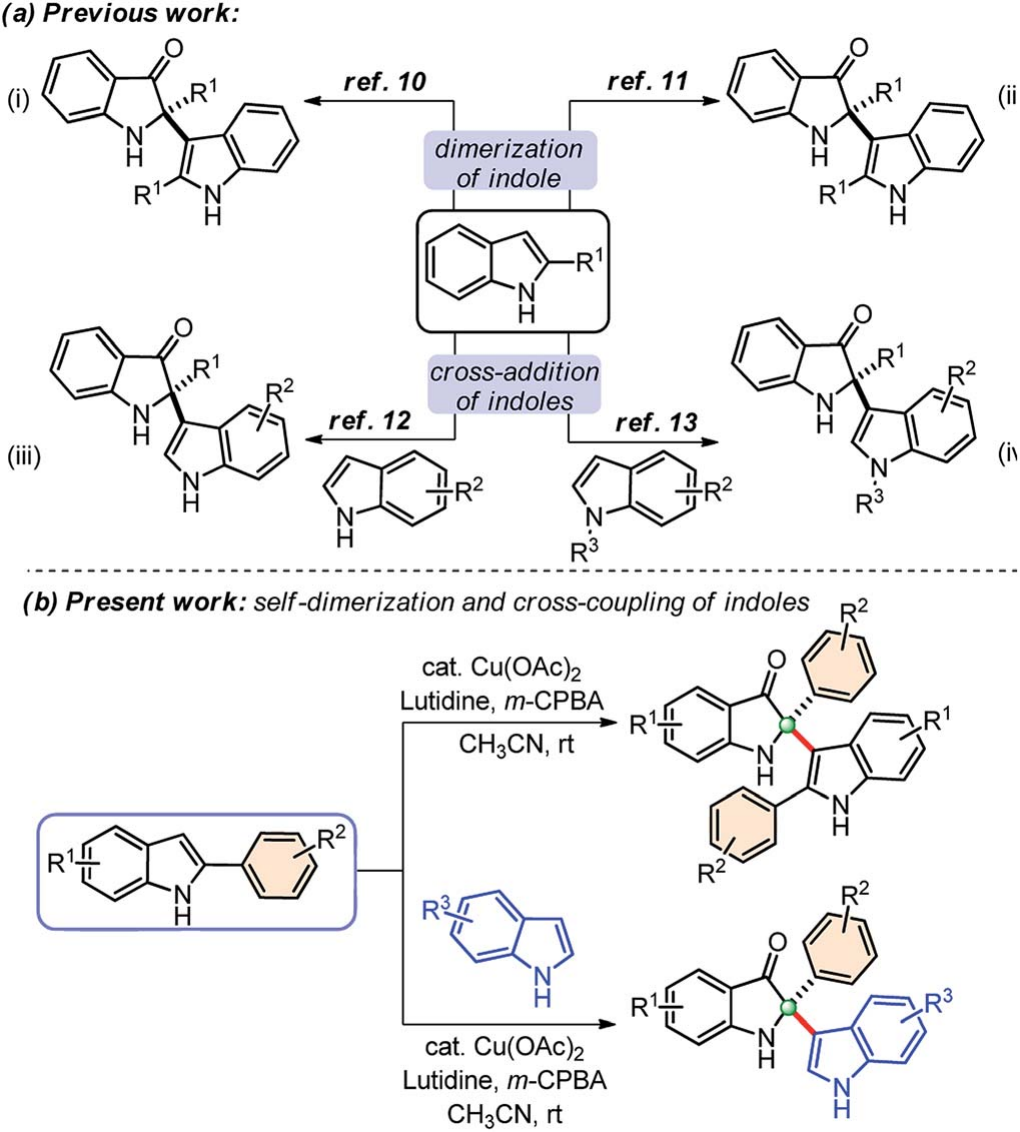
Synthetic approaches from 2-aryl indoles to access 2,2-disubstituted indolin-3-ones.

Owing to the wide utility of 2,2-disubstituted indolin-3-ones, several methods had been developed in the past few years which include; transition-metal catalyzed annulation reactions,^4^ the cascade Fischer indolization/Claisen rearrangement,^5^ and photooxidative rearrangements,^6^ along with other methods.^7^ Besides these methods, chemoselective addition of various nucleophiles to 2-aryl-indol-3-one, an activated cyclic C-acylimine, is another exciting way to access 2,2-disubstituted indolin-3-one derivatives.^8^ However, the synthesis of 2-aryl-3*H*-indol-3-ones requires troublesome multistep syntheses and are not easily accessible.^9^ To overcome this problem, some attention has recently been given to the chemistry of dearomative cascade reactions of 2-substituted indoles for the direct construction of C2-quaternary indolin-3-ones (Scheme 1a). In this context, self-dimerization of 2-substituted indoles have been explored either through Cu-catalysis (Scheme 1a(i))^10^ or other metal-catalysis (Scheme 1a(ii)).^11^ However, these methods required high temperature and sometimes hazardous components. Likewise, oxidative cross-addition of indole to 2-substituted indole could be another way to achieve C2-quaternary indolin-3-ones; however, that is a difficult task to accomplish in terms of selectivity. Guchhait and coworkers developed an exciting and the very first protocol for the cross-addition of indoles to 2-substituted indoles to access 2,2-disubstituted indolin-3-ones with a chiral center under Pd-catalysis in a chemoselective fashion (Scheme 1a(iii)).^12^ Very recently, Yu and coworkers developed a metal-free approach for the cross-addition of indoles with a series of 2-substituted indoles to rapidly access 2,2-disubstituted indolin-3-ones (Scheme 1a(iv)).^13^

Due to the high significance of C2-quaternary indolin-3-ones, and limitations of the existing methods, finding direct access to these compounds through the self-dimerization of 2-substituted indoles or the cross-addition of indoles with 2-substituted indoles under mild conditions, is still highly desirable. Here, we present such a general and straightforward finding for the synthesis of 2,2-disubstituted indolin-3-ones from 2-aryl indoles through copper-catalyzed self-dimerization and cross-addition with indoles at room temperature (Scheme 1(b)).

## Results and discussion

Copper-catalyzed transformations are one of the most studied methods in synthetic chemistry due to their efficiency, good functional group tolerance.^14^ In this context, Cu-catalyzed tandem oxidative reactions of 2-aryl indol-3-ones, *in situ* generated from 2-arylindoles, have been explored to synthesize 2-arylbenzoxazinone,^15^ and polyhydropyrido[1,2-*a*]indoles/tetracyclic quinazolinones.^16^ Encouraged by these relevant precedents, we envisaged that a general copper-catalyzed method could be developed for the self-dimerization of 2-substituted indoles and cross-addition with indoles through the *in situ* generations of indol-3-ones under mild conditions. Herein, we describe the successful implementation of our protocol.

We begin this study for the oxidative cross-dimerization of 2-phenyl indole 1a as model substrate to prepare 2-phenyl-2-(2-phenyl-1*H*-indol-3-yl)indolin-3-one 2a. In this context, optimization of the reaction conditions was carried out by employing several bases, oxidizing agents, catalysts and solvents, and the results are shown in Table 1. Initially, reaction failed to work, when 1a was treated with catalysts CuCl (30 mol%), pyridine with; K_2_S_2_O_8_ (entry 1, Table 1), oxone (entry 2, Table 1). Trace amount (<10%) of the product was obtained with air as oxidants in toluene (entry 3, Table 1) and DMSO (entry 4, Table 1) as solvents, respectively. However, product 2a was obtained with low yield (34%), when the reaction was carried out with CuCl (cat.), pyridine, and TBHP (*tert*-butyl hydroperoxide) in CH_3_CN (entry 5, Table 1) at room temperature. Additional efforts were made to improve the reaction yields either by changing the oxidants, base, and catalysts (entries 6–11, Table 1). An improvement in the reaction yields was observed by employing lutidine, in place of pyridine, with TBHP (45%) (entry 8, Table 1), and with *m*-CPBA (*meta*-chloro perbenzoic acid) (52%) (entry 10, Table 1). The dimerized product 2a was obtained with moderate yield (63%) when Cu(OAc)_2_ (entry 11, Table 1) was employed in place of CuCl as a catalyst with TBHP as oxidant, which was again improved to 75% yield by using *m*-CPBA as oxidant (entry 12, Table 1). Any additional change in the reaction conditions either; by changing oxidant (entry 12, Table 1) or lowering the catalyst loading (entry 14, Table 1) failed to improve the reaction yield. The reaction failed to produce any dimerization product in the absence of catalyst (entry 15, Table 1), and base (entry 16, Table 1). Thus, we preferred to perform this reaction to yield cross-dimerized product 2a under the standardized conditions (entry 12, Table 1). Moreover, reaction only furnished 2-indoles substituted 3-oxindole 2a through the addition of indole as a nucleophile at the C2-position of *in situ* generated indol-3-one in a chemoselective fashion.

**Table 1:** Optimization of reaction conditions^a^

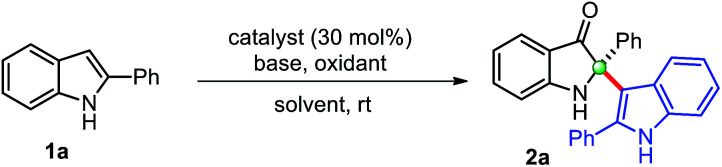						
Entry	Solvent	Catalyst	Base	Oxidant	Time (h)	Yield^b^ (%)
1	Toluene	CuCl	Py	K_2_S_2_O_8_	24	n.r.
2	Toluene	CuCl	Py	Oxone	24	n.r.
3	Toluene	CuCl	Py	Air	24	<10
4	DMSO	CuCl	Py	Air	24	<10
5	CH_3_CN	CuCl	Py	TBHP	24	34
6	CH_3_CN	CuCl	Py	Oxone	24	25
7	CH_3_CN	CuCl	Py	Air	24	30
8	CH_3_CN	CuCl	Lutidine	TBHP	22	45
9	CH_3_CN	CuCl	K_2_CO_3_	TBHP	24	20
10	CH_3_CN	CuCl	Lutidine	*m*-CPBA	20	52
11	CH_3_CN	Cu(OAc)_2_	Lutidine	TBHP	20	63
**12**	**CH**_**3**_ **CN**	**Cu(OAc)**_**2**_	**Lutidine**	***m*-CPBA**	**18**	**75**
13	CH_3_CN	Cu(OAc)_2_	Lutidine	H_2_O_2_	18	43
14^c^	CH_3_CN	Cu(OAc)_2_	Lutidine	*m*-CPBA	24	65
15	CH_3_CN	—	Lutidine	*m*-CPBA	24	n.r.
16	CH_3_CN	Cu(OAc)_2_	—	*m*-CPBA	24	n.r.

a Unless otherwise indicated, the reaction was carried out with 2-phenylindole 1a (0.5 mmol), solvent (3.0 mL), catalyst (30 mol%), base (1.0 mmol), oxidant (0.3 mmol), reaction time (h) at rt.

b Isolated yield of 2a refer to 1a.

c Cu(OAc)_2_ (15 mmol%) was used.

Next, we explored the generality of our developed cross-dimerization protocol with variously substituted 2-aryl indoles 1a–k under standardized conditions, and results are shown in Table 2. The reaction was found to be quite general concerning the substituents on both the aryl-rings of 2-aryl-indole 1 and accomplished within 14–22 h at room temperature to the furnish corresponding cross-dimerized product, *i.e.*, 2-indoles substituted 3-oxindoles 2 in good to high yields (68–82%). Initially, electron-withdrawing substituents like 4-F, 3-Cl, 4-Cl, 3-Br substitutions on the phenyl ring of 2-aryl indole 1b, 1c, 1d, and 1e gave corresponding products 2b (71%), 2c (67%), 2d (69%), and 2e (68%), respectively. The electron-donating substituents like –CH_3_ and –OCH_3_ on the phenyl ring of 2-aryl indoles furnished similar products 2g, 2h, and 2i with relatively higher yields (75–82%) than the substrates with electron-withdrawing groups. The product 2f obtained in high yields (81%) when 5-bromo-2-phenyl-1*H*-indole 1f was employed under standardized conditions. Moreover, densely substituted 2-aryl indole 1j could furnish corresponding cross-dimerized product 2j with moderate (64%) yields, due to the presence of internal steric hindrance. In case of 2-methyl indole as substrate, corresponding dimerized product 2k was also obtained with good yield (71%). The developed protocol was found to be a general w.r.t. the alkyl/aryl substituents at the C2-position of indoles.

**Table 2:** Substrate scope for the self-dimerized synthesis of C2-quaternary indolin-3-ones^a^

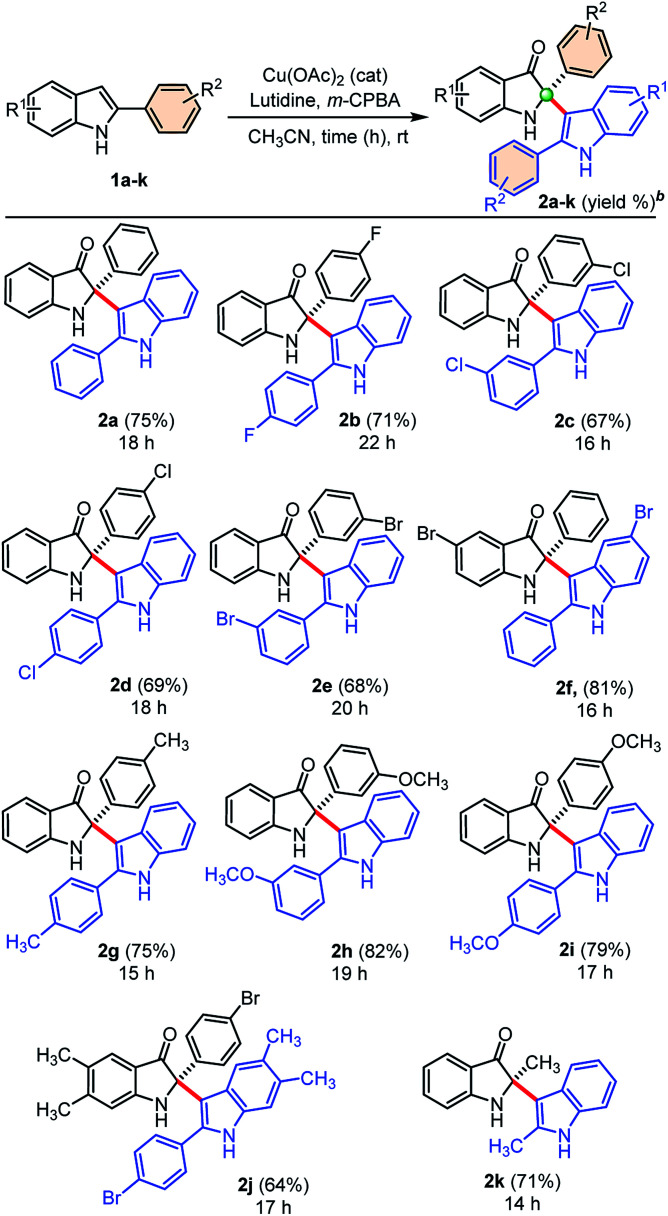

a Unless otherwise indicated, the reaction was carried out with 2-phenylindole 1 (0.5 mmol), CH_3_CN (3.0 mL), Cu(OAc)_2_ (30 mol%), lutidine (1.0 mmol), *m*-CPBA (0.3 mmol), reaction time at rt.

b Isolated yield of 2 refers to 1.

We also extended this developed protocol for the cross addition of indoles 3 at the C2-position of 2-arylindole 1, and the results are shown in Table 3. Pleasingly, cross-addition of indoles furnished similar products in good to high yields, in all the cases, under similar reaction conditions. Initially, indole 3a reacted with 2-aryl indoles (1b, 1d, 1e) substituted with electron-withdrawing groups (–F, –Cl, –Br) and furnished corresponding cross-addition products 4ab (67%), 4ad (66%), and 4ae (68%), respectively. Similarly, products 4ag (69%) and 4ak (62%) were obtained, when simple indole 3a reacted with 2-aryl indoles, 1g and 1k substituted with electron donating groups (–CH_3_, –OH), respectively. Moreover, 5-methoxy indole 3b furnished corresponding cross-addition products 4ba (72%), 4bb (74%), and 4bh (75%) with improved yields, when treated with 2-phenylindole 1a and other substituted 2-aryl indoles (1b and 1h), respectively, in the presence of Cu(OAc)_2_ (cat.), *m*-CPBA (1.0 equiv.), and lutidine (2.0 equiv.) as base at room temperature. Interestingly, the cross-addition reaction of indoles is relatively faster as compared to the cross-dimerization. In general, the self-dimerization products yields were found slightly more that than the cross-addition reactions, because in case of cross-addition of indoles we also observed a trace amount (<10%) of self-dimerized products in almost all the cases.

**Table 3:** Substrate scope for the synthesis of indolin-3-ones through indole-addition^a^

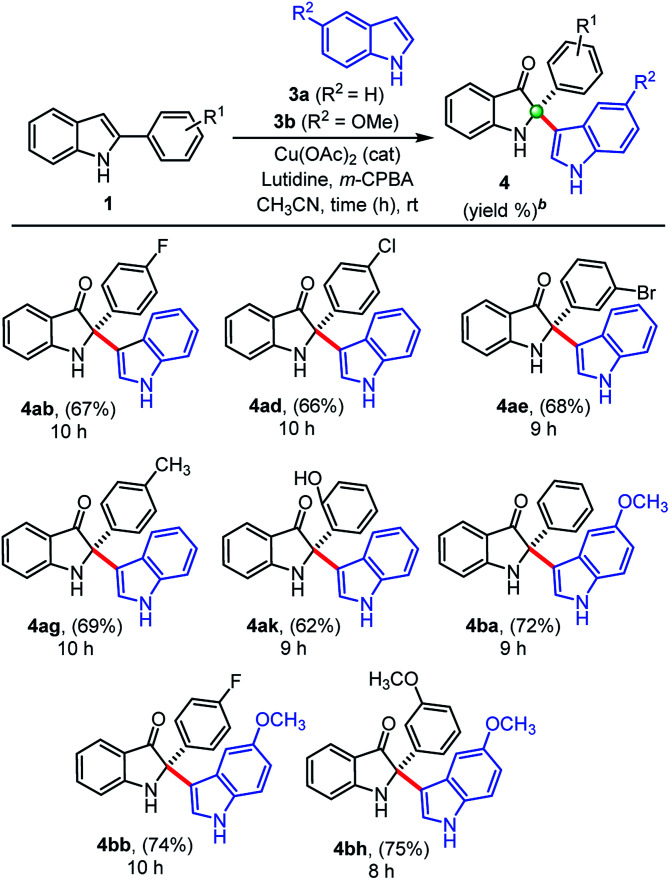

a Unless otherwise indicated, the reaction was carried out with 2-phenylindole 1 (0.5 mmol), indole 3 (0.55 mmol), CH_3_CN (3.0 mL), Cu(OAc)_2_ (30 mol%), lutidine (1.0 mmol), *m*-CPBA (0.5 mmol), reaction time (h) at rt.

b Isolated yield of 4 refers to 1.

**Scheme 2:**
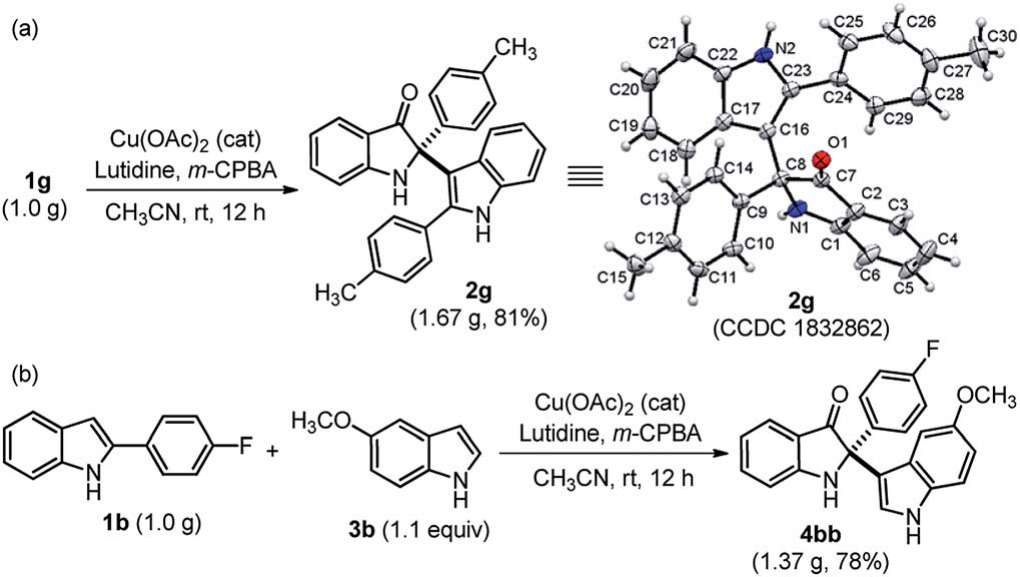
Practical utility at the gram-scale synthesis of 2g and 4bb. Single crystal X-ray structure of 2g (the thermal ellipsoids are drawn at the 50% probability level).

The practical use of this method was also demonstrated to access both cross-dimerization of 2-arylindole and cross-addition of indole with 2-arylindole products on a gram-scale without much variation in yield, as shown in Scheme 2. Pleasingly, the gram-scale reaction successfully afforded 2g with a higher yield (81%) (Scheme 2a) as compared to the small-scale response (Table 2). The single-crystal X-ray diffraction analysis confirmed the structure of cross-dimerized product 2g.^17^ Moreover, 2,2-disubstituted indole-3-one 4bb was isolated with 78% yield, when cross-addition of 2-substituted indole 1b was performed with 4-methoxy indole 3b, under standardized conditions (Scheme 2b).

**Scheme 3:**
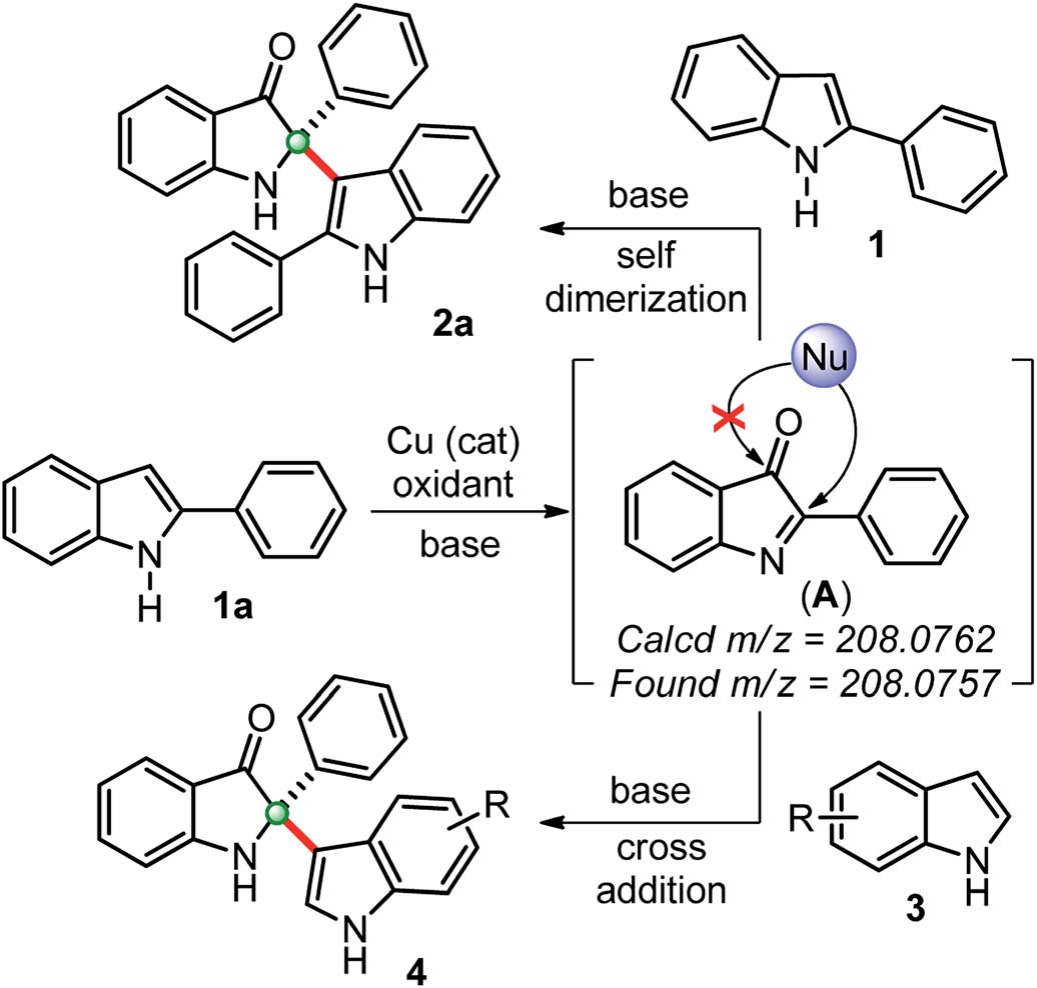
Plausible way of reaction for the synthesis of 2,2-disubstituted indole-2-ones through *in situ* generations of indole-3-one confirmed by HRMS.

Based on previous reports and our findings, a tentative mechanism for the synthesis of self-dimerized 2-phenyl-2-(2-phenyl-1*H*-indol-3-yl)indolin-3-ones 2 and cross-addition 2-(1*H*-indol-3-yl)-2-phenylindole-3-ones 4 is drawn, as shown in Scheme 3. Initially, the copper-catalyzed oxidation of 2-phenylindole 1a to intermediate 2-phenylindole-3-one (A), which was confirmed by *in situ* HRMS data. This intermediate (A) undergoes a chemoselective C2-addition with another 2-phenylindole 1a to furnished self-dimerization product 2a. Whereas, for the cross-addition product 4, intermediate (A) was trapped with another indole moiety.

## Conclusions

In summary, we have developed an efficient and general protocol for the synthesis of 2,2-disubstituted indolin-3-ones through the self-dimerization of 2-aryl indoles and cross-addition of 2-aryl indoles with indoles. This reaction proceeds with *in situ* generation of indol-3-one, followed by chemoselective nucleophilic addition under mild copper-catalyzed conditions. This simple strategy provides convenient and either way to access indolin-3-ones bearing C2-quaternary center in good to high yields. The developed protocol utilized nontoxic, readily available materials, and practically viable at the gram-scale synthesis.

## Experimental

### General remarks

All reactions were observed using thin-layer chromatography (on SiO_2_ gel F254 plates) under standard condition. The desired compounds were purified through flash column chromatography packed with silica gel (100–200 meshes size) as the stationary phase and eluting solvent, hexane–ethyl acetate solvent mixture was used as mobile phase. Melting points were determined in open capillary tubes on an EZ-Melt Automated melting point apparatus and are uncorrected. NMR spectra were recorded on a Bruker AV 400 spectrometer. Chemical shifts were reported in parts per million (ppm) using deuterated solvent or tetramethylsilane (TMS) as an internal standard. High-resolution mass spectra (HRMS-ESI) were recorded using quadrupole time-of-flight (Q-TOF) mass spectrometer (Applied Biosystem). All the chemicals were obtained from the commercial supplier and were used without purification.

### Typical procedure for the synthesis of oxidative dimerized product 2

To a stirred solution of 2-phenylindole 1 (0.5 mmol) in CH_3_CN (3.0 mL) was added lutidine (1.0 mmol), Cu(OAc)_2_ (30 mol%) and *m*-CPBA (*meta*-chloroperoxybenzoic acid, 0.3 mmol) successively at room temperature. The combined reaction mixture was stirred at the same temperature until TLC confirmed the complete consumption of starting material. Subsequently, the reaction was quenched with H_2_O (3.0 mL) and stirred with EtOAc (10 mL). The organic layer was separated, and the aqueous layer was again extracted with EtOAc (5.0 mL). The combined organic extracts were washed with brine, dried over Na_2_SO_4_ anhydrous, and concentrated under reduced pressure. Column chromatography purification through silica gel by eluting the mixture of hexane/EtOAc gave corresponding dimerized product 2 as mainly yellow solid with 64–82% yields.

#### 2-Phenyl-2-(2-phenyl-1*H*-indol-3-yl)indolin-3-one (2a)

Yellow solid (165 mg, 75% yield, mp = 207–212 °C). ^1^H NMR (400 MHz, CDCl_3_) *δ* 5.17 (s, 1H), 6.71 (d, *J* = 8.2 Hz, 1H), 6.80 (t, *J* = 7.7 Hz, 1H), 6.91–6.93 (m, 1H), 7.00 (d, *J* = 8.0 Hz, 1H), 7.10–7.20 (m, 8H), 7.23–7.27 (m, 1H), 7.32 (d, *J* = 8.1 Hz, 1H), 7.40 (d, *J* = 7.7 Hz, 1H), 7.42–7.46 (m, 1H), 7.48–7.51 (m, 2H), 8.11 (s, 1H). ^13^C NMR (100 MHz, CDCl_3_) *δ* 72.0, 110.7, 112.0, 112.2, 119.1, 120.0, 120.4, 121.5, 122.3, 125.4 (2C), 127.2 (2C), 127.3, 127.5, 127.6 (2C), 128.2 (2C), 129.7 (2C), 133.2, 135.5, 137.0, 137.1, 140.3, 159.4, 200.3. HRMS (ESI-TOF) *m*/*z*: calcd for C_28_H_21_N_2_O [M + H]^+^ 401.1654, found 401.1637.

#### 2-(4-Fluorophenyl)-2-(2-(4-fluorophenyl)-1*H*-indol-3-yl)indolin-3-one (2b)

Yellow solid (175 mg, 71% yield, mp = 175–180 °C). ^1^H NMR (400 MHz, CDCl_3_) *δ* 5.20 (s, 1H), 6.77–6.95 (m, 8H), 7.05 (dd, *J* = 8.8, 5.3 Hz, 2H), 7.14–7.18 (m, 1H), 7.29–7.37 (m, 2H), 7.45–7.51 (m, 3H), 8.16 (s, 1H). ^13^C NMR (100 MHz, CDCl_3_) *δ* 71.3, 110.8, 112.4, 112.6, 114.5, 114.7, 114.9, 115.1, 119.6, 120.2, 120.4, 121.0, 122.6, 125.4, 127.1, 128.9, 128.9, 131.5, 131.6, 135.4, 135.8 (2C), 136.0, 137.4, 159.2, 162.4 (d, *J* = 246 Hz), 162.6 (d, *J* = 246 Hz), 200.1. HRMS (ESI-TOF) *m*/*z*: calcd for C_28_H_19_F_2_N_2_O [M + H]^+^ 437.1465, found 437.1445.

#### 2-(3-Chlorophenyl)-2-(2-(3-chlorophenyl)-1*H*-indol-3-yl)indolin-3-one (2c)

Yellow solid (156 mg, 67% yield, mp = 160–164 °C). ^1^H NMR (400 MHz, CDCl_3_) *δ* 5.21 (s, 1H), 6.86–6.91 (m, 2H), 6.94 (d, *J* = 4.0 Hz, 2H), 7.02 (m, 5H), 7.15–7.21 (m, 2H), 7.33 (d, *J* = 8.1 Hz, 1H), 7.38 (dt, *J* = 8.0 Hz, 4.0 Hz, 1H), 7.46 (d, *J* = 7.3 Hz, 1H), 7.50–7.55 (m, 2H), 8.20 (s, 1H). ^13^C NMR (100 MHz, CDCl_3_) *δ* 71.3, 111.0, 112.3, 112.6, 120.0, 120.4, 120.4, 120.9, 122.8, 125.4, 125.8, 126.9, 127.1, 127.7, 127.8, 128.4, 128.8, 129.4, 129.9, 133.6, 134.0, 134.5, 135.5, 135.9, 137.7, 141.93, 159.3, 199.9. HRMS (ESI-TOF) calcd for C_28_H_19_Cl_2_N_2_O *m*/*z*: [M + H]^+^ 469.0874, found 469.0848.

#### 2-(4-Chlorophenyl)-2-(2-(4-chlorophenyl)-1*H*-indol-3-yl)indolin-3-one (2d)

Yellow solid (161 mg, 69% yield, mp = 197–201 °C). ^1^H NMR (400 MHz, CDCl_3_) *δ* 5.20 (s, 1H), 6.85–6.89 (m, 2H), 6.91–6.99 (m, 2H), 7.03 (d, *J* = 8.5 Hz, 2H), 7.13 (d, *J* = 8.5 Hz, 2H), 7.17–7.22 (m, 3H), 7.35–7.41 (m, 2H), 7.47 (d, *J* = 8.7 Hz, 2H), 7.50–7.54 (m, 1H), 8.14 (s, 1H). ^13^C NMR (100 MHz, CDCl_3_) *δ* 71.40, 110.9, 120.3, 119.7, 120.3, 120.4, 121.0, 122.7, 125.4, 127.1, 127.8 (2C), 128.3 (2C), 128.6 (2C), 131.0 (2C), 131.2, 132.0, 133.6, 134.5, 135.5, 135.8, 137.5, 138.6, 159.2, 199.7. HRMS (ESI-TOF) *m*/*z*: calcd for C_28_H_19_Cl_2_N_2_O [M + H]^+^ 469.0874, found 469.0853.

#### 2-(3-Bromophenyl)-2-(2-(3-bromophenyl)-1*H*-indol-3-yl)indolin-3-one (2e)

Yellow solid (188 mg, 68% yield, mp = 122–126 °C). ^1^H NMR (400 MHz, CDCl_3_) *δ* 5.25 (s, 1H), 6.90–6.93 (m, 2H), 6.97 (d, *J* = 3.9 Hz, 2H), 7.00–7.06 (m, 2H), 7.11 (d, *J* = 7.7 Hz, 1H), 7.17–7.21 (m, 2H), 7.26–7.27 (m, 1H), 7.33–7.38 (m, 2H), 7.44 (d, *J* = 7.9 Hz, 1H), 7.48 (d, *J* = 7.7 Hz, 1H), 7.53–7.57 (m, 1H), 7.71 (t, *J* = 1.8 Hz, 1H), 8.27 (s, 1H). ^13^C NMR (100 MHz, CDCl_3_) *δ* 71.3, 111.0, 112.3, 112.7, 120.1, 120.3, 120.4, 120.9, 121.8, 122.3, 122.8, 125.5, 126.2, 126.9, 128.1, 129.0, 129.7, 129.9, 130.7, 131.3, 132.7, 134.7, 135.5, 135.9, 137.7, 142.1, 159.3, 199.9. HRMS (ESI-TOF) *m*/*z*: calcd for C_28_H_19_Br_2_N_2_O [M + H]^+^ 556.9864, found 556.9836.

#### 5-Bromo-2-(5-bromo-2-phenyl-1*H*-indol-3-yl)-2-phenylindolin-3-one (2f)

Yellow solid (225 mg, 81% yield, mp = 148–153 °C). ^1^H NMR (400 MHz, CDCl_3_) *δ* 5.13 (s, 1H), 6.58 (d, *J* = 8.5 Hz, 1H), 7.04 (d, *J* = 1.4 Hz, 1H), 7.11–7.15 (m, 2H), 7.19–7.25 (m, 7H), 7.29–7.33 (m, 1H), 7.41–7.43 (m, 2H), 7.48–7.51 (m, 2H), 8.15 (s, 1H). ^13^C NMR (100 MHz, CDCl_3_) *δ* 72.5, 111.2, 111.4, 112.1, 113.3, 113.7, 121.7, 124.0, 125.4, 127.0 (2C), 127.7, 127.9 (2C), 128.1, 128.5 (2C), 128.7, 128.9, 129.6 (2C), 132.6, 134.1, 138.1, 139.5, 139.7, 157.5, 198.6. HRMS (ESI-TOF) *m*/*z*: calcd for C_28_H_19_Br_2_N_2_O [M + H]^+^ 556.9864, found 556.9836.

#### 2-(*p*-Tolyl)-2-(2-(*p*-tolyl)-1*H*-indol-3-yl)indolin-3-one (2g)

Yellow solid (215 mg, 75% yield, mp = 159–161 °C). ^1^H NMR (400 MHz, CDCl_3_) *δ* 2.27 (s, 3H), 2.33 (s, 3H), 5.17 (s, 1H), 6.68 (d, *J* = 8.2 Hz, 1H), 6.78 (t, *J* = 7.4 Hz, 1H), 6.89–7.05 (m, 8H), 7.13 (t, *J* = 8.0 Hz, 1H), 7.28 (d, *J* = 8.1 Hz, 1H), 7.34–7.40 (m, 3H), 7.41–7.45 (m, 1H), 8.10 (s, 1H). ^13^C NMR (100 MHz, CDCl_3_) *δ* 21.0, 21.2, 71.9, 110.6, 111.8, 112.1, 118.8, 119.7, 120.3, 121.6 (2C), 122.1, 125.3, 127.1 (2C), 127.4, 128.3 (2C), 128.9 (2C), 129.5, 130.3, 135.4, 136.9, 137.0, 137.1, 137.4, 137.9, 159.1, 200.4. HRMS (ESI-TOF) *m*/*z*: calcd for C_30_H_25_N_2_O [M + Na]^+^ 451.1787, found 451.1760.

#### 2-(3-Methoxyphenyl)-2-(2-(3-methoxyphenyl)-1*H*-indol-3-yl)indolin-3-one (2h)

Yellow solid (188 mg, 82% yield, mp = 206–210 °C). ^1^H NMR (400 MHz, CDCl_3_) *δ* 3.56 (s, 3H), 3.67 (s, 3H), 5.20 (s, 1H), 6.64 (m, 1H), 6.69–6.74 (m, 2H), 6.78–6.83 (m, 3H), 6.94 (t, *J* = 7.3 Hz, 1H), 7.05–7.17 (m, 6H), 7.32 (d, *J* = 8.2 Hz, 1H), 7.42–7.47 (m, 2H), 8.10 (s, 1H). ^13^C NMR (100 MHz, CDCl_3_) *δ* 54.9, 55.1, 71.9, 110.6, 111.7, 112.2, 112.8, 113.1, 114.7 (2C), 119.2, 119.7, 120.0, 120.4, 121.50, 121.7, 122.3, 125.3, 127.3, 128.8, 129.1, 134.5, 135.4, 136.9, 137.1, 141.9, 158.6, 159.2, 159.3, 200.1. HRMS (ESI-TOF) *m*/*z*: calcd for C_30_H_25_N_2_O_3_ [M + H]^+^ 461.1865, found 461.1843.

#### 2-(4-Methoxyphenyl)-2-(2-(4-methoxyphenyl)-1*H*-indol-3-yl)indolin-3-one (2i)

Yellow solid (181 mg, 79% yield, mp = 102–105 °C). ^1^H NMR (400 MHz, CDCl_3_) *δ* 3.73 (s, 3H), 3.77 (s, 3H), 5.18 (s, 1H), 6.65 (d, *J* = 8.7 Hz, 2H), 6.71–6.74 (m, 3H), 6.78 (t, *J* = 7.2 Hz, 1H), 6.90–6.99 (m, 2H), 7.02 (d, *J* = 8.7 Hz, 2H), 7.11–7.15 (m, 1H), 7.30 (d, *J* = 8.1 Hz, 1H), 7.37–7.46 (m, 4H), 8.07 (s, 1H). ^13^C NMR (100 MHz, CDCl_3_) *δ* 55.2, 55.3, 71.6, 110.6, 112.1, 112.2, 113.1 (2C), 113.6 (2C), 119.0, 119.8, 120.5, 121.5, 122.1, 125.4, 125.5, 127.5, 128.4 (2C), 130.9 (2C), 132.4, 135.4, 137.0, 136.8, 159.0, 159.1, 159.5, 200.6. HRMS (ESI-TOF) *m*/*z*: calcd for C_30_H_25_N_2_O_3_ [M + H]^+^ 461.1865, found 461.1851.

#### 2-(4-Bromophenyl)-2-(2-(4-bromophenyl)-5,6-dimethyl-1*H*-indol-3-yl)-5,6-dimethylindolin-3-one (2j)

Yellow solid (196 mg, 64% yield, mp = 192–195 °C). ^1^H NMR (400 MHz, CDCl_3_) *δ* 2.16 (s, 3H), 2.21 (s, 3H), 2.30 (s, 3H), 2.31 (s, 3H), 4.94 (s, 1H), 6.66 (d, *J* = 8.7 Hz 2H), 6.94 (d, *J* = 8.4 Hz, 2H), 7.11 (s, 1H), 7.16 (s, 1H), 7.25 (d, *J* = 8.4 Hz, 2H), 7.28 (d, *J* = 8.7 Hz, 2H), 7.36 (d, *J* = 8.7 Hz, 2H), 7.85 (s, 1H). ^13^C NMR (100 MHz, CDCl_3_) *δ* 19.2, 20.3 (2C), 21.3, 71.7, 111.1, 112.1, 113.4, 118.8, 121.0, 121.5, 122.4, 125.0, 125.4, 126.3, 128.9, 129.0 (2C), 130.6 (2C), 131.1 (2C), 131.3 (2C), 131.9, 132.0, 134.5, 134.9, 139.7, 148.2, 158.5, 199.3. HRMS (ESI-TOF) *m*/*z*: [M + H]^+^ calcd for C_32_H_27_Br_2_N_2_O 613.0490, found 615.0433.

#### 2-Methyl-2-(2-methyl-1*H*-indol-3-yl)indolin-3-one (2k)

Yellow solid (145 mg, 71% yield, mp = 151–153 °C). ^1^H NMR (400 MHz, CDCl_3_) *δ* 1.95 (s, 3H), 2.45 (s, 3H), 5.05 (s, 1H), 6.89–6.92 (m, 2H), 6.96–7.00 (m, 1H), 7.07–7.11 (m, 1H), 7.24–7.28 (m, 1H), 7.42 (dd, *J* = 8.1, 1.0 Hz, 1H), 7.52–7.56 (m, 1H), 7.73 (dd, *J* = 8.1 Hz, 1.0 Hz, 1H), 7.90 (s, 1H). ^13^C NMR (100 MHz, CDCl_3_) *δ* 14.6, 25.1, 67.14, 109.5, 110.4, 111.4, 112.4, 119.1, 119.5, 119.7, 121.2, 125.3, 127.4, 132.6, 134.9, 137.4, 159.5, 204.3. HRMS (ESI-TOF) *m*/*z*: calcd for C_18_H_17_N_2_O [M + H]^+^ 277.1341, found 277.1334.

### Typical procedure for the synthesis of (4)

To a stirred solution of 2-phenylindole 1 (0.5 mmol) in CH_3_CN (3.0 mL) was added lutidine (1.0 mmol), Cu(OAc)_2_ (30 mol%) and *m*-CPBA (0.5 mmol) successively, followed by substituted indole 3 (1.1 equiv.) at room temperature and progress of the reaction was monitored by TLC. Once the reaction was over, it was quenched with water (3.0 mL) and stirred with EtOAc (10 mL). The organic layer was separated, and the aqueous layer was again extracted with EtOAc (5.0 mL). The combined organic extracts were washed with brine and dried over anhydrous Na_2_SO_4_, followed by concentrated in the vacuum after filtration. Purification through silica-gel column chromatography by eluting the mixture of hexane/EtOAc gave corresponding product 4 as mainly yellow solid with 62–75% yields.

#### 2-(4-Fluorophenyl)-2-(1*H*-indol-3-yl)indolin-3-one (4ab)

Yellow solid (114 mg, 67% yield, mp = 206–210 °C). ^1^H NMR (400 MHz, CDCl_3_) *δ* 5.36 (s, 1H) 6.90–7.06 (m, 5H), 7.15–7.13 (m, 3H), 7.41 (d, *J* = 8.2 Hz, 1H), 7.50–7.60 (m, 3H), 7.72 (d, *J* = 7.7 Hz, 1H), 8.20 (s, 1H). ^13^C NMR (100 MHz, CDCl_3_) *δ* 70.7, 111.7, 113.0, 115.1, 115.3, 115.5, 119.5 (2C), 119.9, 120.2, 122.7, 123.6, 125.46, 125.6, 128.5, 128.6, 135.3, 136.9, 137.6 (2C), 160.5, 200.3. HRMS (ESI-TOF) *m*/*z*: calcd for C_22_H_16_FN_2_O [M + H]^+^ 343.1246, found 343.1236.

#### 2-(4-Chlorophenyl)-2-(1*H*-indol-3-yl)indolin-3-one (4ad)

Yellow solid (118 mg, 66% yield, mp = 123–1126 °C). ^1^H NMR (400 MHz, CDCl_3_) *δ* 5.35 (s, 1H), 6.91 (t, *J* = 8.2 Hz, 2H), 6.98–7.03 (m, 1H), 7.12–7.13 (m, 2H), 7.17–7.21 (m, 1H), 7.24–7.26 (m, 2H), 7.37 (d, *J* = 8.2 Hz, 1H), 7.49–7.53 (m, 3H), 7.69 (d, *J* = 7.7 Hz, 1H), 8.26 (s, 1H). ^13^C NMR (100 MHz, CDCl_3_) *δ* 70.7, 111.7, 113.1, 115.1, 119.4 (2C), 119.9, 120.1, 122.6, 123.6, 125.3, 125.6, 128.2 (2C), 128.5 (2C), 133.6, 136.9, 137.7, 138.1, 160.5, 200.1. HRMS (ESI-TOF) *m*/*z*: calcd for C_22_H_16_ClN_2_O [M + H]^+^ 359.0951, found 359.0951.

#### 2-(3-Bromophenyl)-2-(1*H*-indol-3-yl)indolin-3-one (4ae)

Yellow solid (137 mg, 68% yield, mp = 135–138 °C). ^1^H NMR (400 MHz, CDCl_3_) *δ* 5.38 (s, 1H), 6.92–9.97 (m, 2H), 7.01–7.05 (m, 1H), 7.15–7.23 (m, 4H), 7.38–7.44 (m, 2H), 7.48–7.52 (m, 1H), 7.52–7.56 (m, 1H), 7.72 (d, *J* = 7.7 Hz, 1H), 7.81 (t, *J* = 3.6 Hz, 1H), 8.30 (s, 1H). ^13^C NMR (100 MHz, CDCl_3_) *δ* 70.7, 111.8, 113.2, 115.0, 119.4, 119.4, 120.0, 120.2, 122.5, 122.7, 123.7, 125.3, 125.6, 126.0, 129.4, 130.0, 130.8, 136.9, 137.8, 142.0, 160.5, 199.9. HRMS (ESI-TOF) *m*/*z*: calcd for C_22_H_16_BrN_2_O [M + H]^+^ 403.0446, found 403.0441.

#### 2-(1*H*-Indol-3-yl)-2-(*p*-tolyl)indolin-3-one (4ag)

Yellow solid (122 mg, 69% yield, mp = 103–107 °C). ^1^H NMR (400 MHz, CDCl_3_) *δ* 2.31 (s, 3H), 5.39 (s, 1H), 6.86–6.91 (m, 2H), 6.95–7.01 (m, 1H), 7.09–7.11 (m, 3H), 7.14–7.19 (m, 2H), 7.34 (d, *J* = 8.1 Hz, 1H), 7.43 (d, *J* = 8.2 Hz, 2H), 7.47–751 (m, 1H), 7.69 (d, *J* = 7.3 Hz, 1H), 8.29 (s, 1H). ^13^C NMR (100 MHz, CDCl_3_) *δ* 21.1, 71.2, 111.7, 112.8, 115.5, 119.5, 119.5, 119.8, 119.9, 122.4, 123.8, 125.6 (2C), 126.7 (2C), 129.2 (2C), 136.5, 136.9, 137.4, 137.5, 160.5, 200.9. HRMS (ESI-TOF) *m*/*z*: calcd for C_23_H_18_N_2_O [M + H]^+^ 339.1497, found 339.1492.

#### 2-(2-Hydroxyphenyl)-2-(1*H*-indol-3-yl)indolin-3-one (4ak)

Yellow solid (105 mg, 62% yield, mp = 107–111 °C).^1^H NMR (400 MHz, CDCl_3_) *δ* 2.08 (s, 1H), 5.42 (s, 1H), 6.90–7.01 (m, 6H), 7.09 (d, *J* = 7.9 Hz, 1H), 7.12–7.16 (m, 1H), 7.23–7.31 (m, 2H), 7.56–7.60 (m, 1H), 7.71 (dd, *J* = 7.9 Hz, 1.6 Hz, 1H), 7.76 (d, *J* = 7.8 Hz, 1H), 8.39 (s, 1H). ^13^C NMR (100 MHz, CDCl_3_) *δ* 71.6, 111.8, 122.5, 113.4, 113.7, 118.8, 119.0, 120.1, 120.2, 120.7, 124.0, 124.5, 124.9, 125.6, 128.1, 129.9, 136.8, 138.7, 156.0, 160.1, 171.3, 204.0. HRMS (ESI-TOF) *m*/*z*: calcd for C_22_H_17_N_2_O_2_ [M + H]^+^ 341.1290, found 341.1286.

#### 2-(5-Methoxy-1*H*-indol-3-yl)-2-phenylindolin-3-one (4ba)

Yellow solid (128 mg, 72% yield, mp = 91–94 °C). ^1^H NMR (400 MHz, CDCl_3_) *δ* 3.63 (s, 3H), 5.39 (s, 1H), 6.58 (d, *J* = 2.2 Hz, 1H), 6.85 (dd, *J* = 8.8 Hz, 2.4 Hz, 1H), 6.92 (t, *J* = 7.4 Hz, 1H), 6.96 (d, *J* = 8.2 Hz, 1H), 7.12 (d, *J* = 2.6 Hz, 1H), 7.26–7.34 (m, 4H), 7.51–7.55 (m, 1H), 7.62 (dd, *J* = 8.0 Hz, 1.5 Hz, 2H), 7.72 (d, *J* = 7.7 Hz, 1H), 8.14 (s, 1H). ^13^C NMR (100 MHz, CDCl_3_) *δ* 55.5, 71.2, 101.8, 112.3, 112.3, 112.8, 115.4, 119.6, 119.7, 124.4, 125.5, 126.0, 126.84 (2C), 127.7, 128.4 (2C), 131.9, 137.5, 139.3, 154.2, 160.5, 200.1. HRMS (ESI-TOF) *m*/*z*: calcd for C_23_H_19_N_2_O_2_ [M + H]^+^ 355.1446, found 355.1429.

#### 2-(4-Fluorophenyl)-2-(5-methoxy-1*H*-indol-3-yl)indolin-3-one (4bb)

Yellow solid (137mg, 74% yield, mp = 201–204 °C).^1^H NMR (400 MHz, CDCl_3_) *δ* 3.65 (s, 3H), 5.37 (s, 1H), 6.55 (d, *J* = 2.3 Hz, 1H), 6.85 (dd, *J* = 8.8 Hz, 2.4 Hz, 1H), 6.91–7.03 (m, 4H), 7.09 (d, *J* = 2.6 Hz, 1H), 7.27 (d, *J* = 9.6 Hz, 1H), 7.52–7.60 (m, 3H), 7.72 (d, *J* = 7.7 Hz, 1H), 8.19 (s, 1H). ^13^C NMR (100 MHz, CDCl_3_) *δ* 55.6, 70.7, 101.7, 112.3, 112.4, 113.1, 115.1, 115.2, 115.3, 119.7, 119.9, 124.4, 125.5, 125.9, 128.6, 128.7, 132.0, 135.1, 137.7, 154.1, 160.5, 162.5 (d, *J* = 246 Hz), 200.6. HRMS (ESI-TOF) *m*/*z*: calcd for C_23_H_18_FN_2_O_2_ [M + H]^+^ 373.1352, found 373.1334.

#### 2-(5-Methoxy-1*H*-indol-3-yl)-2-(3-methoxyphenyl)indolin-3-one (4bh)

Yellow solid (140 mg, 75% yield, mp = 91–95 °C). ^1^H NMR (400 MHz, CDCl_3_) *δ* 3.62 (s, 3H), 3.74 (s, 3H), 5.32 (s, 1H), 6.59 (d, *J* = 2.4 Hz, 1H), 6.80–6.84 (m, 2H), 6.89 (t, *J* = 7.7 Hz, 1H), 6.93 (d, *J* = 8.2 Hz, 1H), 7.11 (d, *J* = 2.7 Hz, 1H), 7.16–7.21 (m, 2H), 7.23–7.25 (m, 1H), 7.27 (s, 1H) 7.48–7.52 (m, 1H), 7.69 (d, *J* = 7.7 Hz, 1H), 8.01 (s, 1H). ^13^C NMR (100 MHz, CDCl_3_) *δ* 55.2, 55.6, 71.1, 101.9, 112.2, 112.3, 112.8 (2C), 112.9, 115.5, 119.3, 119.6, 119.9, 124.4, 125.5, 126.0, 129.3, 131.9, 137.4, 141.0, 154.0, 159.6, 160.4, 200.4. HRMS (ESI-TOF) *m*/*z*: calcd for C_24_H_21_N_2_O_3_ [M + H]^+^ 385.1552, found 385.1535.

## Conflicts of interest

There are no conflicts to declare.

## Supplementary Material

RA-009-C9RA04741G-s001

RA-009-C9RA04741G-s002

## References

[cit1] Lee J. H., So J.-H., Jeon J. H., Choi E. B., Lee Y.-R., Chang Y.-T., Kim C.-H., Bae M. A., Ahn J. H. (2011). Chem. Commun..

[cit2] Liu J.-F., Jiang Z.-Y., Wang R.-R., Zeng Y.-T., Chen J.-J., Zhang X.-M., Ma Y.-B. (2007). Org. Lett..

[cit3] Wyrembak P. N., Hamilton A. D. (2009). J. Am. Chem. Soc..

[cit4] Wetzel A., Gagosz F. (2011). Angew. Chem., Int. Ed..

[cit5] Xia Z., Hu J., Gao Y. Q., Yao Q., Xie W. (2017). Chem. Commun..

[cit6] Lerch S., Unkel L.-N., Brasholz M. (2014). Angew. Chem., Int. Ed..

[cit7] Liu Y., Jr McWhorter W. W. (2003). J. Org. Chem..

[cit8] Li L.-Q., Han M.-Y., Xiao M.-X., Xie Z.-X. (2011). Synlett.

[cit9] Ling K.-Q. (1995). Synth. Commun..

[cit10] Sang P., Xie Y., Zou J., Zhang Y. (2012). Adv. Synth. Catal..

[cit11] Lin F., Chen Y., Wang B., Qin W., Liu L. (2015). RSC Adv..

[cit12] Guchhait S. K., Chaudhary V., Rana V. A., Priyadarshani G., Kandekar S., Kashyap M. (2016). Org. Lett..

[cit13] Jiang X., Zhu B., Lin K., Wang G., Su W.-K., Yu C. (2019). Org. Biomol. Chem..

[cit14] Wendlandt W. A., Suess A. M., Stahl S. S. (2011). Angew. Chem., Int. Ed..

[cit15] Yamashita M., Iida A. (2014). Tetrahedron Lett..

[cit16] Yamashita M., Nishizono Y., Himekawa S., Iida A. (2016). Tetrahedron.

[cit17] The X-ray crystallographic structure for 2g has been deposited at the Cambridge Crystallographic Data Centre (CCDC), under deposition numbers CCDC 1832862.†

