# Supplementary material for: Synthesis of C2-tetrasubstituted indolin-3-ones via Cu-catalyzed oxidative dimerization of 2-aryl indoles and cross-addition with indoles
Source: RSC Adv. 2019 Aug 2;9(42):24050–6. doi: 10.1039/c9ra04741g (PMC9069668; doi:10.1039/c9ra04741g)
Supplement: RA-009-C9RA04741G-s001 [file RA-009-C9RA04741G-s001.pdf]

Electronic Supplementary Material (ESI) for RSC Advances.
This journal is © The Royal Society of Chemistry 2019

Supporting Information

Synthesis of C2-tetrasubstituted indolin-3-ones via Cu-catalyzed oxidative dimerization of 2-aryl indoles and cross-addition of indoles

AnoopSingh,[a§] SatheeshvarmaVanaparthi,[a§] SachinChoudhary,[a] RanganKrishnan,[b] and Indresh Kumar*[a]

[a]*Department of Chemistry, Birla Institute of Technology & Science, Pilani 333 031, India*

[b]*Department of Chemistry, BITS Pilani, Hyderabad Campus, Secunderabad, India*

[§]*authors have contributed equally*

Corresponding Email: *indresh.kumar@pilani.bits-pilani.ac.in*, *indresh.chemistry@gmail.com*

Table of Contents

^{1}H, ^{13}C-NMRand HRMS data for the newly synthesized compounds……………..………..…......S2

Single Crystal X-ray and data for **2g**……………………………………………………………...S40

HRMS Facility, BITS Pilani, Pilani Campus
Compound Report

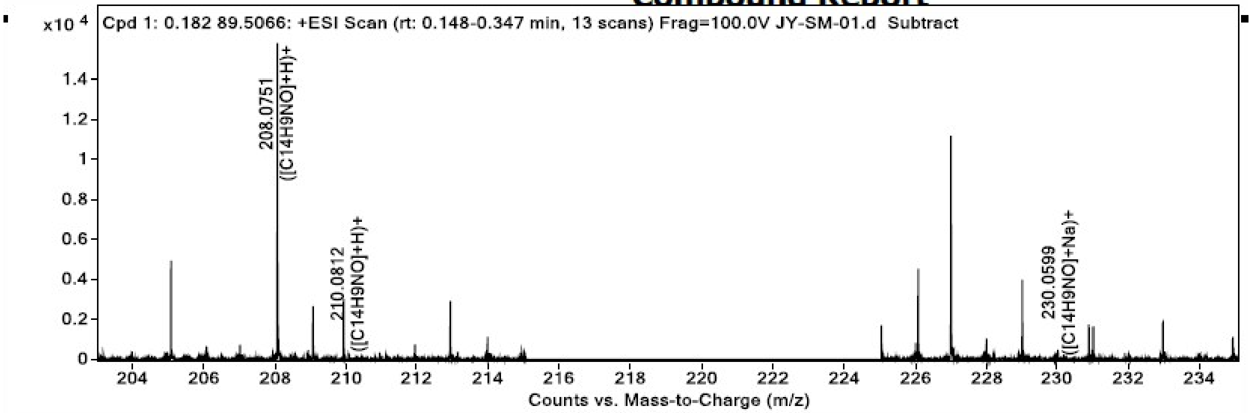

MS Spectrum Peak List

m/z	*Calc m/z*	Diff(ppm)	z	Abund	Formula	Ion
208.0751	208.0757	2.63	1	16179.21	C14H9NO	(M+H)+
209.0794	209.0789	-2.3	1	2670.21	C14H9NO	(M+H)+
210.0812	210.0818	3.01	1	238.68	C14H9NO	(M+H)+
230.0599	230.0576	-9.85	1	59.78	C14H9NO	(M+Na)+

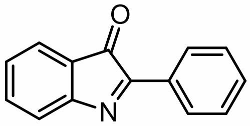

Calcd m/z = 208.0762
Found m/z = 208.0757

Instrument Info : Agilent Technologies 6545 Q-TOF LC/MS

Figure S1: HRMS confirmation of in situ generated 2-phenyl indole-3-one

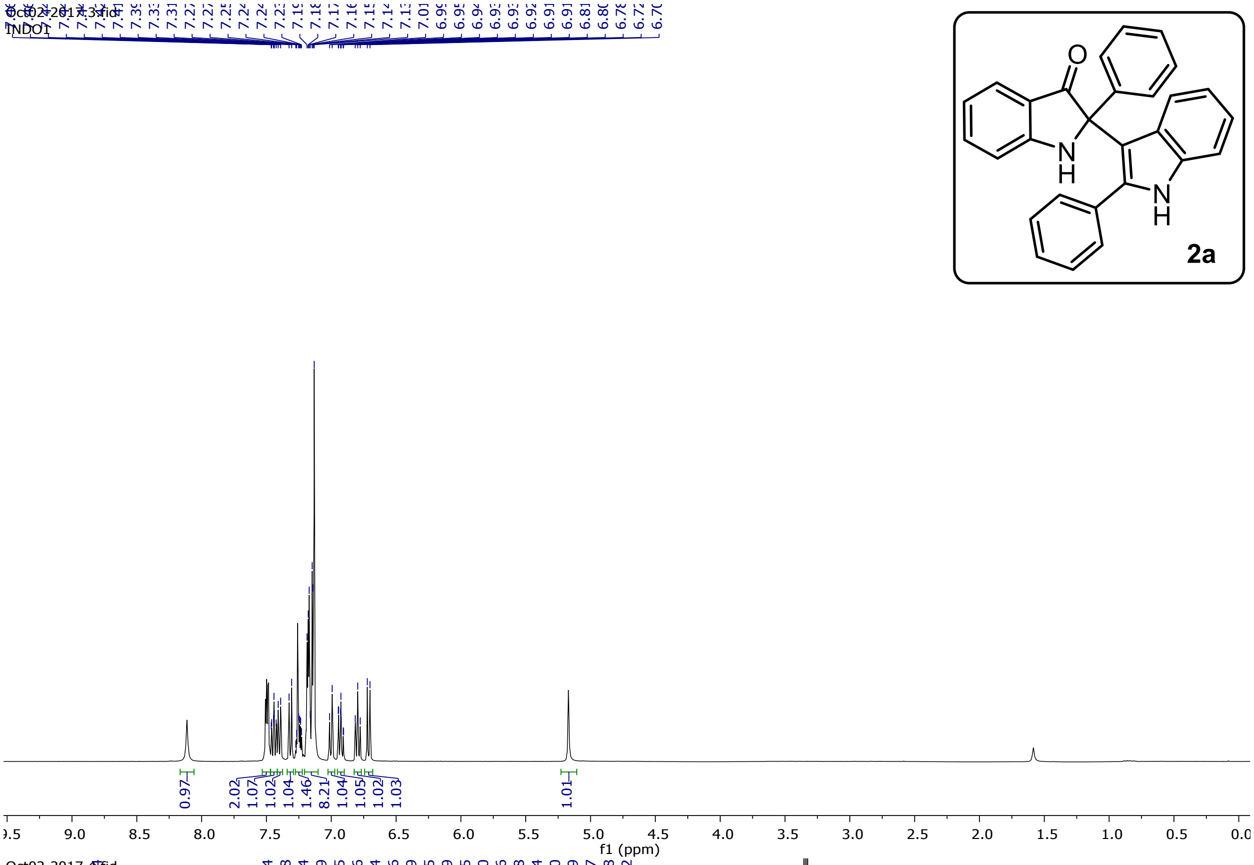
Oct02-2017.3.fid
INDO1
0.97
2.02
1.07
1.02
1.04
1.46
8.21
1.04
1.05
1.02
1.03
1.01
f1 (ppm)
O
NH
H
N
H
2a

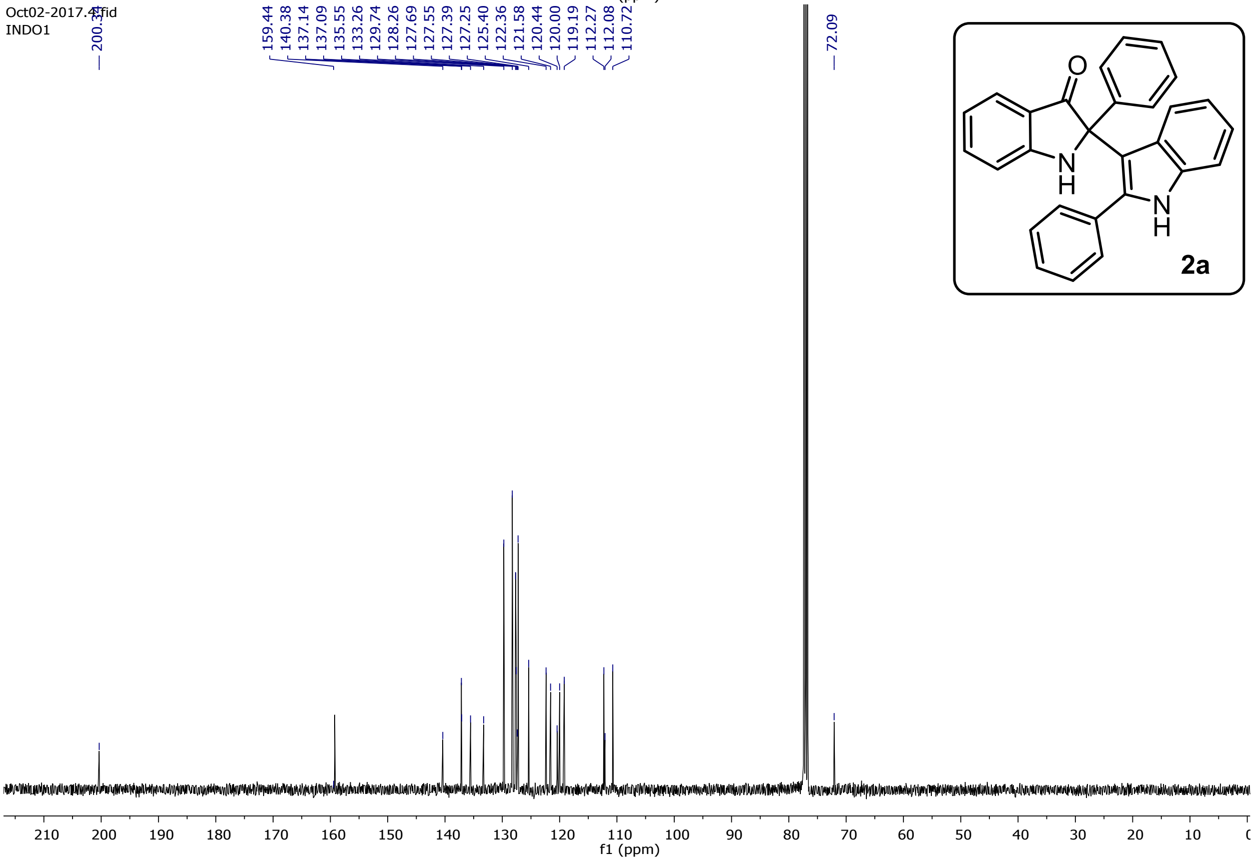
Oct02-2017.4.fid
INDO1
72.09
159.44
140.38
137.14
137.09
135.55
133.26
129.74
128.26
127.69
127.55
127.39
127.25
125.40
122.36
121.58
120.44
120.00
119.19
112.27
112.08
110.72
f1 (ppm)
O
NH
H
N
H
2a

Qualitative Compound Report

**Data File**	IND 01.d	**Sample Name**	IND 01
**Sample Type**	Sample	**Position**	P2-D3
**Instrument Name**	Instrument 1	**User Name**	
**Acq Method**	water_meoh_grad_6min_reg.m	**Acquired Time**	1/5/2018 11:05:38 AM
**IRM Calibration Status**	Success	**DA Method**	PROCESSNEW.m
**Comment**			

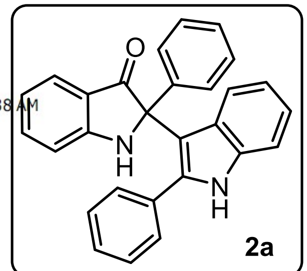

2a

**Sample Group**		**Info.**	
**Stream Name**	LC 1	**Acquisition SW Version**	6200 series TOF/6500 series Q-TOF B.06.01 (B6172 SP1)

Compound Table

Compound Label	RT	Mass	Abund	Formula	Tgt Mass	Diff (ppm)
Cpd 1: 1.611 401.1637	1.611	400.1565	112812	C28 H20 N2 O	400.1576	-2.73

Compound Label	*m/z*	RT	Algorithm	Mass
Cpd 1: 1.611 401.1637	401.1637	1.611	Find By Formula	400.1565

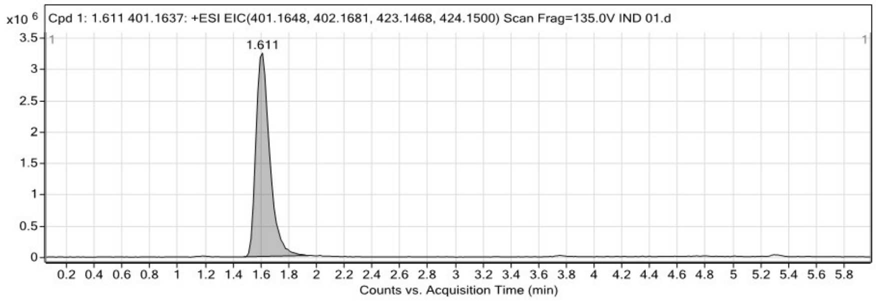

MS Zoomed Spectrum

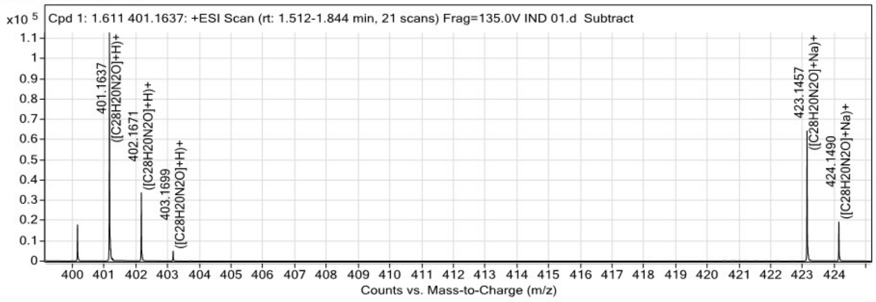

MS Spectrum Peak List

m/z	*Calc m/z*	Diff(ppm)	z	Abund	Formula	Ion
401.1637	401.1648	2.91	1	112812.26	C28H20N2O	(M+H)+
402.1671	402.1681	2.42	1	33913.1	C28H20N2O	(M+H)+

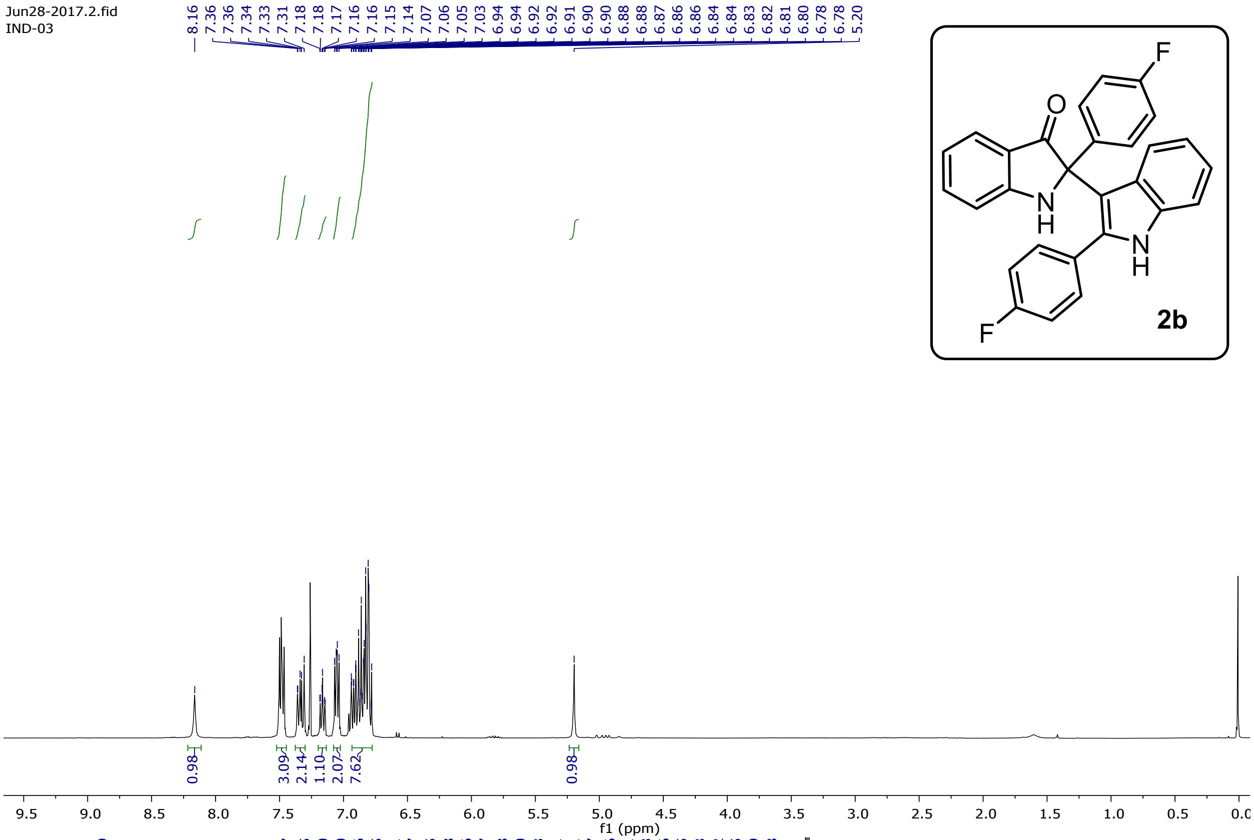
Jun28-2017.2.fid
IND-03
8.16
7.36
7.36
7.34
7.33
7.31
7.18
7.18
7.17
7.16
7.16
7.15
7.14
7.07
7.06
7.05
7.03
6.94
6.94
6.92
6.92
6.91
6.90
6.90
6.88
6.88
6.87
6.86
6.86
6.84
6.84
6.83
6.82
6.81
6.80
6.78
6.78
5.20
0.98
3.09
2.14
1.10
2.07
7.62
0.98
9.5
9.0
8.5
8.0
7.5
7.0
6.5
6.0
5.5
5.0
4.5
4.0
3.5
3.0
2.5
2.0
1.5
1.0
0.5
0.0
f1 (ppm)
F
O
N
H
N
H
F
2b

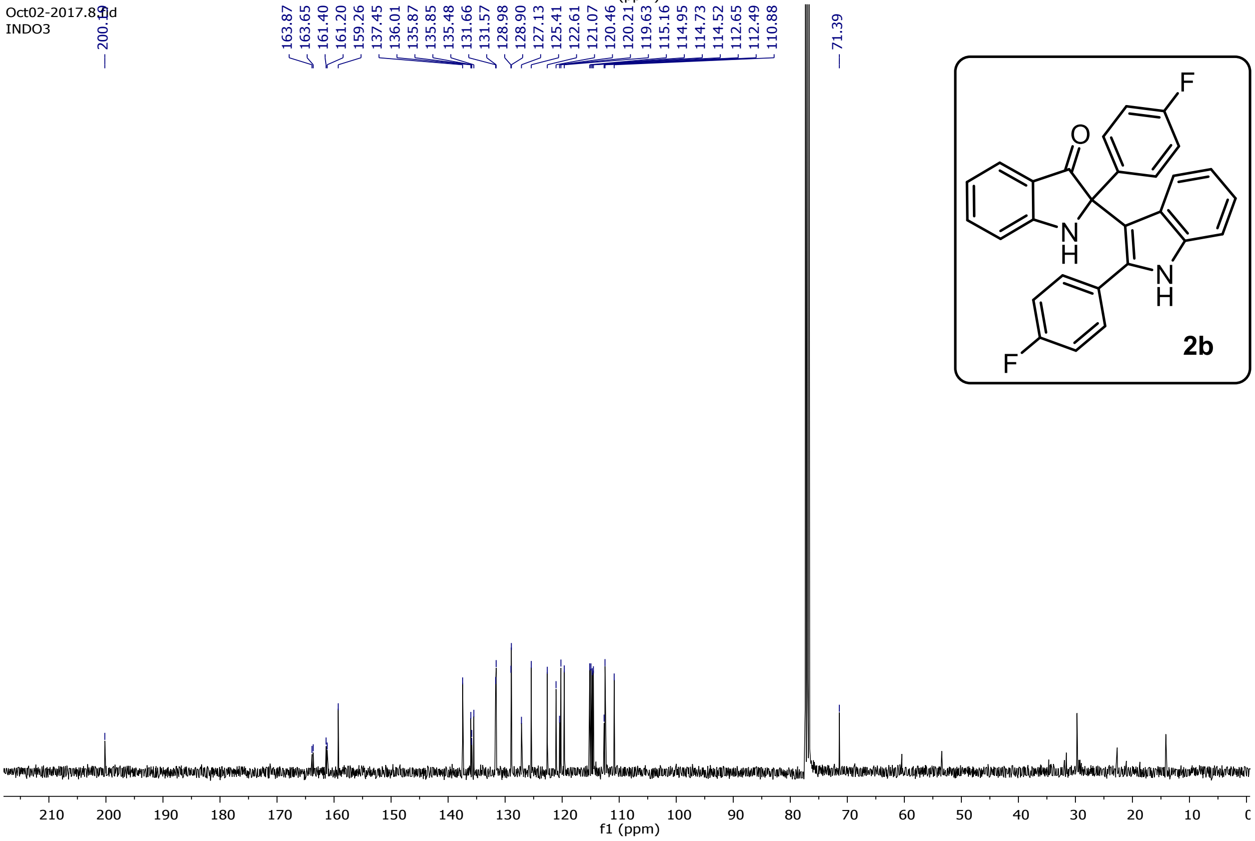
Oct02-2017.8.fid
INDO3
200.19
163.87
163.65
161.40
161.20
159.26
137.45
136.01
135.87
135.85
135.48
131.66
131.57
128.98
128.90
127.13
125.41
122.61
121.07
120.46
120.21
119.63
115.16
114.95
114.73
114.52
112.65
112.49
110.88
71.39
210
200
190
180
170
160
150
140
130
120
110
100
90
80
70
60
50
40
30
20
10
0
f1 (ppm)
F
O
N
H
N
H
F
2b

Qualitative Compound Report

**Data File**	IND 03.d	**Sample Name**	IND 03
**Sample Type**	Sample	**Position**	P2-D5
**Instrument Name**	Instrument 1	**User Name**	
**Acq Method**	water_meoh_grad_6min_reg.m	**Acquired Time**	1/5/2018 11:33:51 AM
**IRM Calibration Status**	Success	**DA Method**	PROCESSNEW.m
**Comment**			

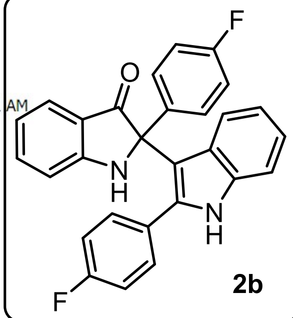

2b

**Sample Group**		**Info.**	
**Stream Name**	LC 1	**Acquisition SW Version**	6200 series TOF/6500 series Q-TOF B.06.01 (B6172 SP1)

Compound Table

Compound Label	RT	Mass	Abund	Formula	Tgt Mass	Diff (ppm)
Cpd 1: 1.894 437.1445	1.894	436.1373	108534	C28 H18 F2 N2 O	436.1387	-3.2

Compound Label	*m/z*	RT	Algorithm	Mass
Cpd 1: 1.894 437.1445	459.1266	1.894	Find By Formula	436.1373

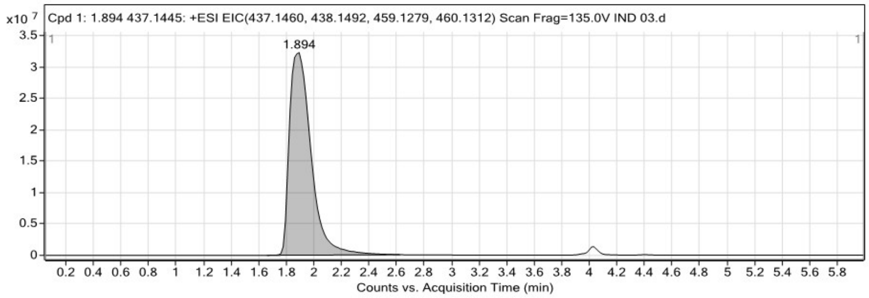

MS Zoomed Spectrum

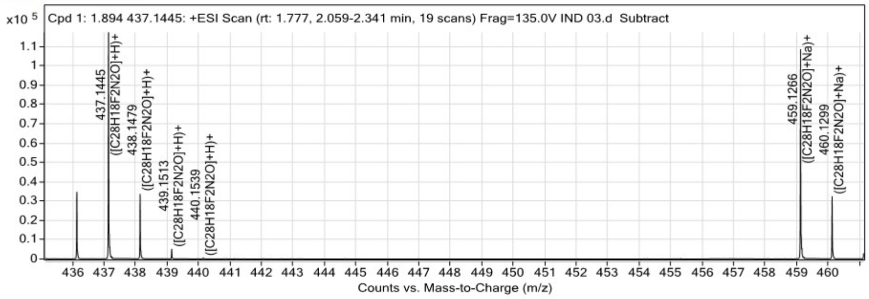

MS Spectrum Peak List

m/z	*Calc m/z*	Diff(ppm)	z	Abund	Formula	Ion
437.1445	437.146	3.47	1	118437.31	C28H18F2N2O	(M+H)+
438.1479	438.1492	3.1	1	34135.9	C28H18F2N2O	(M+H)+

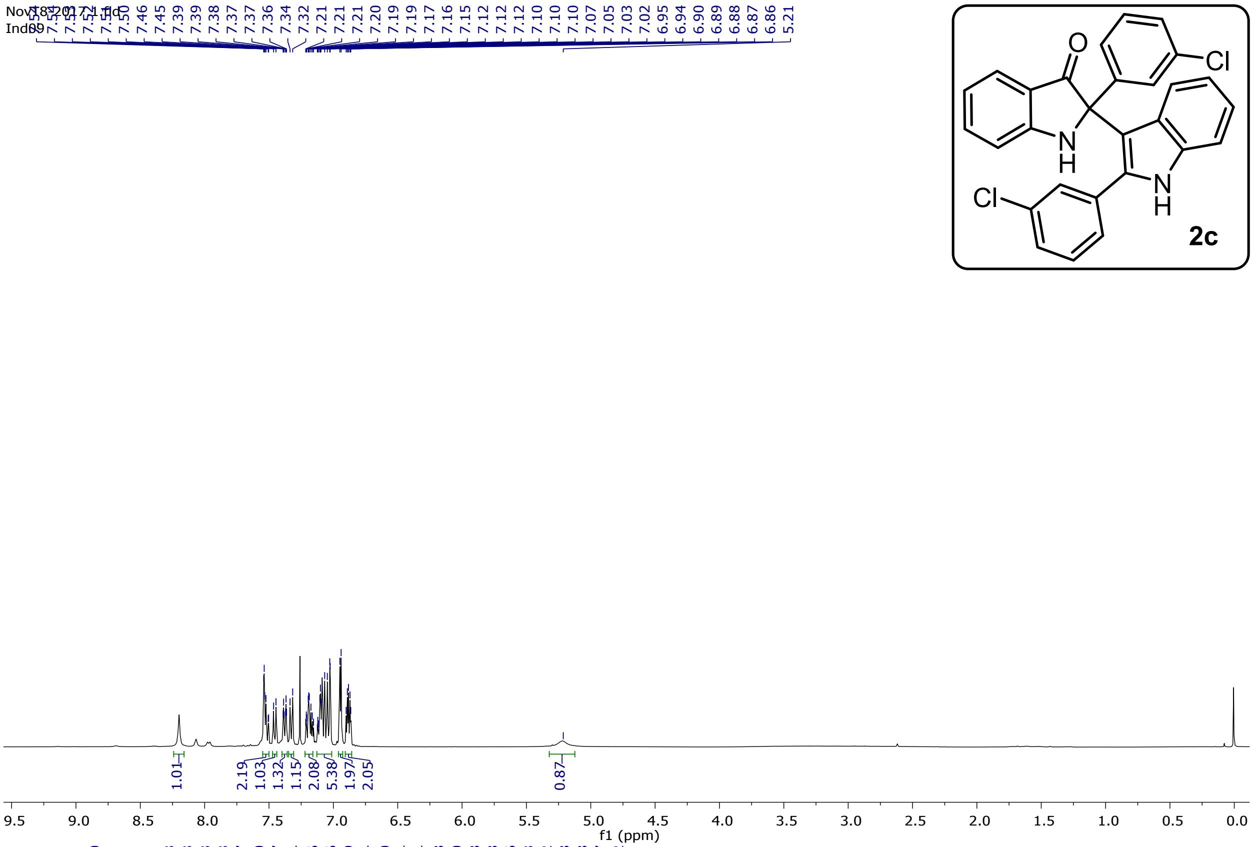

Nov18-2017.1.fid
Ind09
1.01
2.19
1.03
1.32
1.15
2.08
5.38
1.97
2.05
0.87
f1 (ppm)
O
Cl
N
H
2c

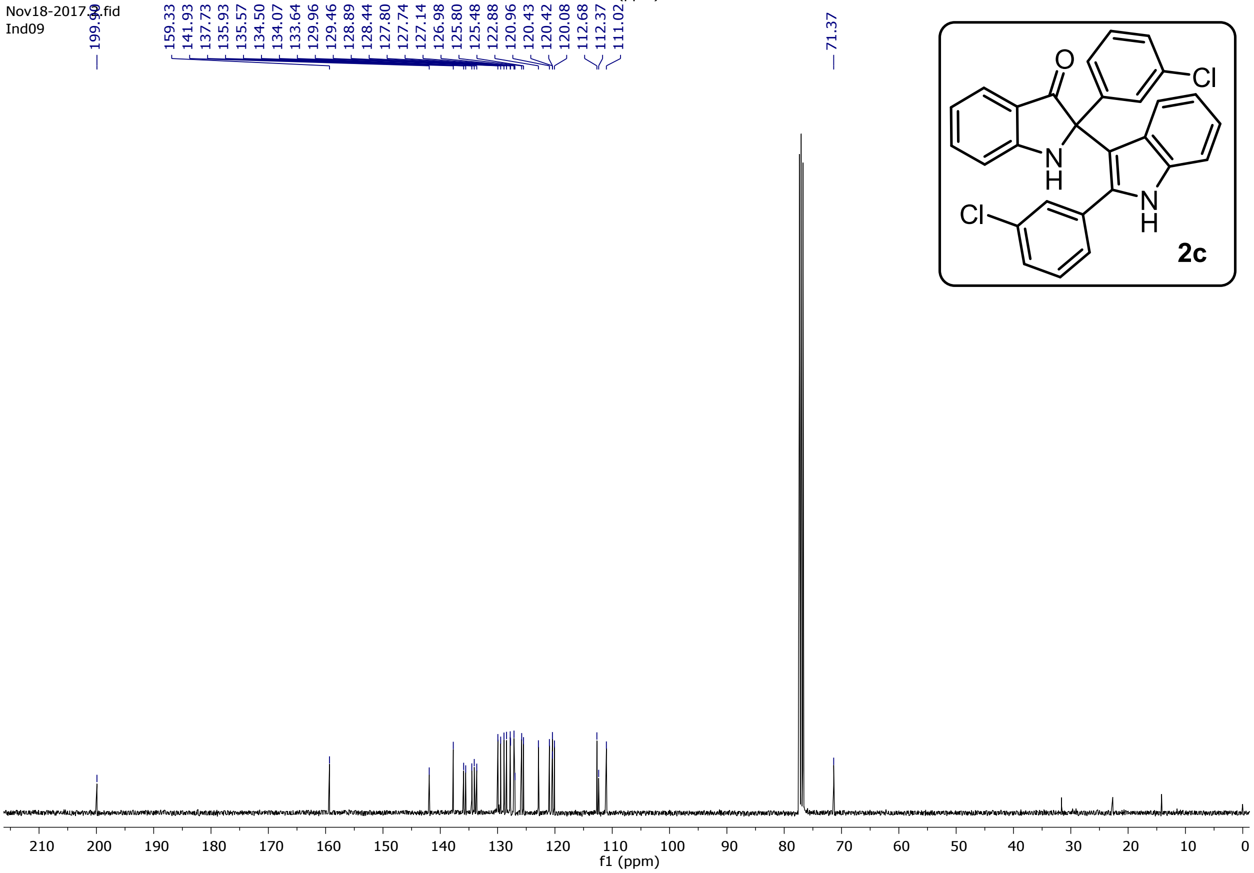

Nov18-2017.2.fid
Ind09
159.33
141.93
137.73
135.93
135.57
134.50
134.07
133.64
129.96
129.46
128.89
128.44
127.80
127.74
127.14
126.98
125.80
125.48
122.88
120.96
120.43
120.42
120.08
112.68
112.37
111.02
71.37
f1 (ppm)
O
Cl
N
H
2c

Qualitative Compound Report

**Data File**	IND 09.d	**Sample Name**	IND 09
**Sample Type**	Sample	**Position**	P2-D8
**Instrument Name**	Instrument 1	**User Name**	
**Acq Method**	water_meoh_grad_6min_reg.m	**Acquired Time**	1/5/2018 12:16:12 PM
**IRM Calibration Status**	Success	**DA Method**	PROCESSNEW.m
**Comment**			

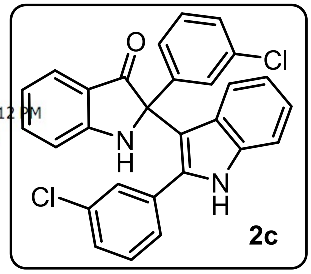

2c

**Sample Group**		**Info.**	
**Stream Name**	LC 1	**Acquisition SW Version**	6200 series TOF/6500 series Q-TOF B.06.01 (B6172 SP1)

Compound Table

Compound Label	RT	Mass	Abund	Formula	Tgt Mass	Diff (ppm)
Cpd 1: 3.083 491.0672	3.083	468.0774	59963	C28 H18 Cl2 N2 O	468.0796	-4.7

Compound Label	*m/z*	RT	Algorithm	Mass
Cpd 1: 3.083 491.0672	491.0672	3.083	Find By Formula	468.0774

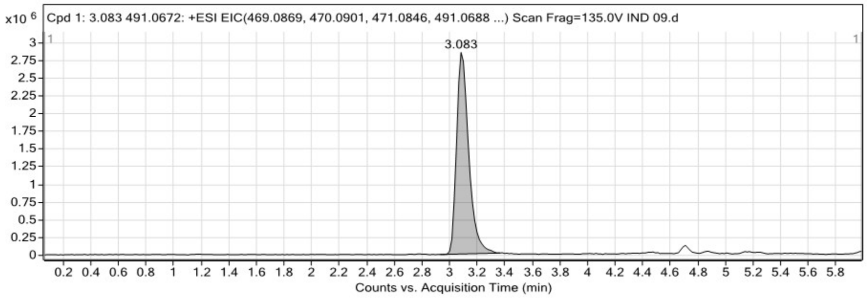

MS Zoomed Spectrum

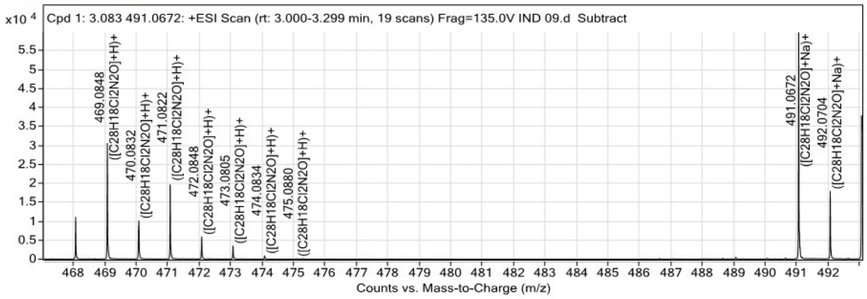

MS Spectrum Peak List

m/z	*Calc m/z*	Diff(ppm)	z	Abund	Formula	Ion
469.0848	469.0869	4.46	1	30947.37	C28H18Cl2N2O	(M+H)+
470.0832	470.0901	14.75	1	10161.32	C28H18Cl2N2O	(M+H)+

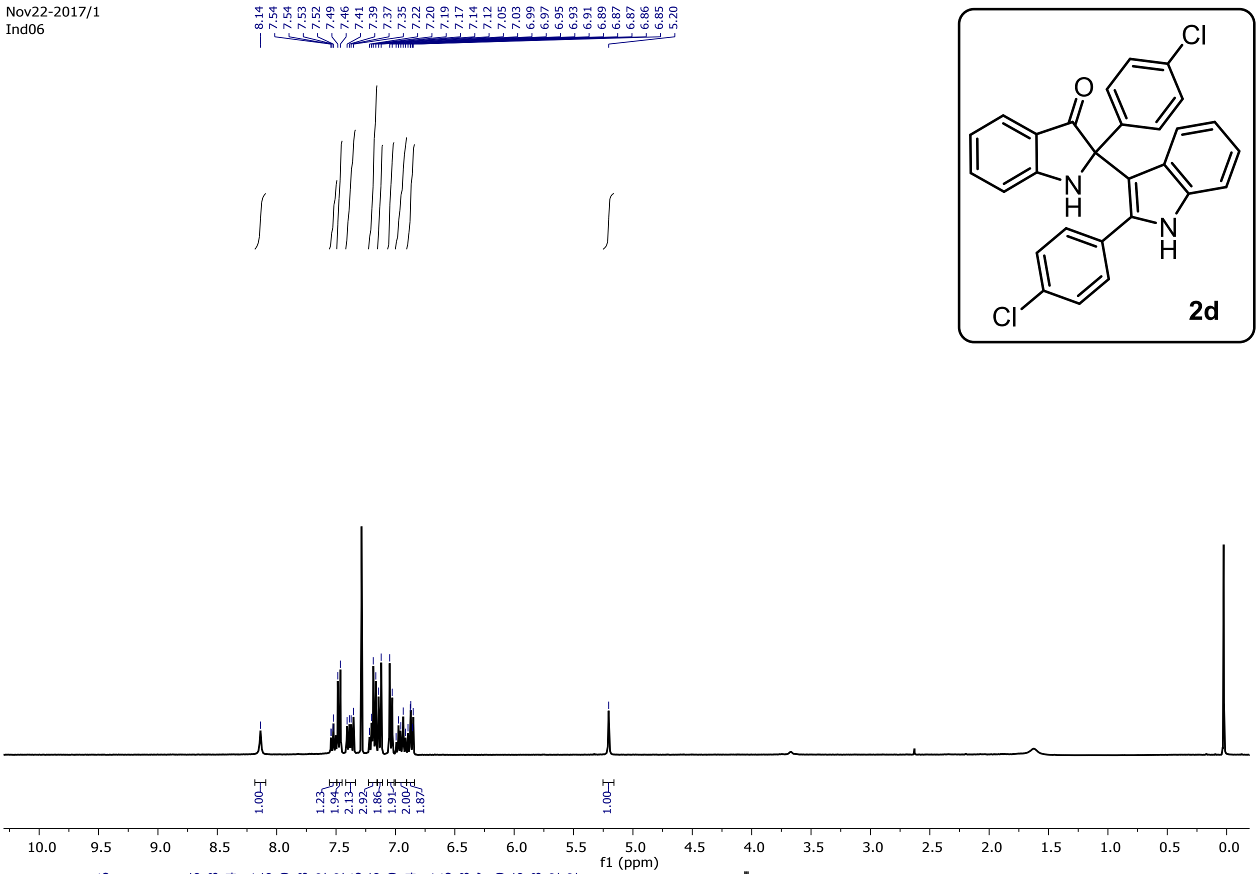
Nov22-2017/1
Ind06
8.14 7.54 7.54 7.53 7.52 7.49 7.46 7.41 7.39 7.37 7.35 7.22 7.20 7.19 7.17 7.14 7.12 7.05 7.03 6.99 6.97 6.95 6.93 6.91 6.89 6.87 6.86 6.85 5.20
1.00 1.23 1.94 2.13 2.92 1.86 1.91 2.00 1.87 1.00
f1 (ppm)
Cl
O
N
H
Cl
2d

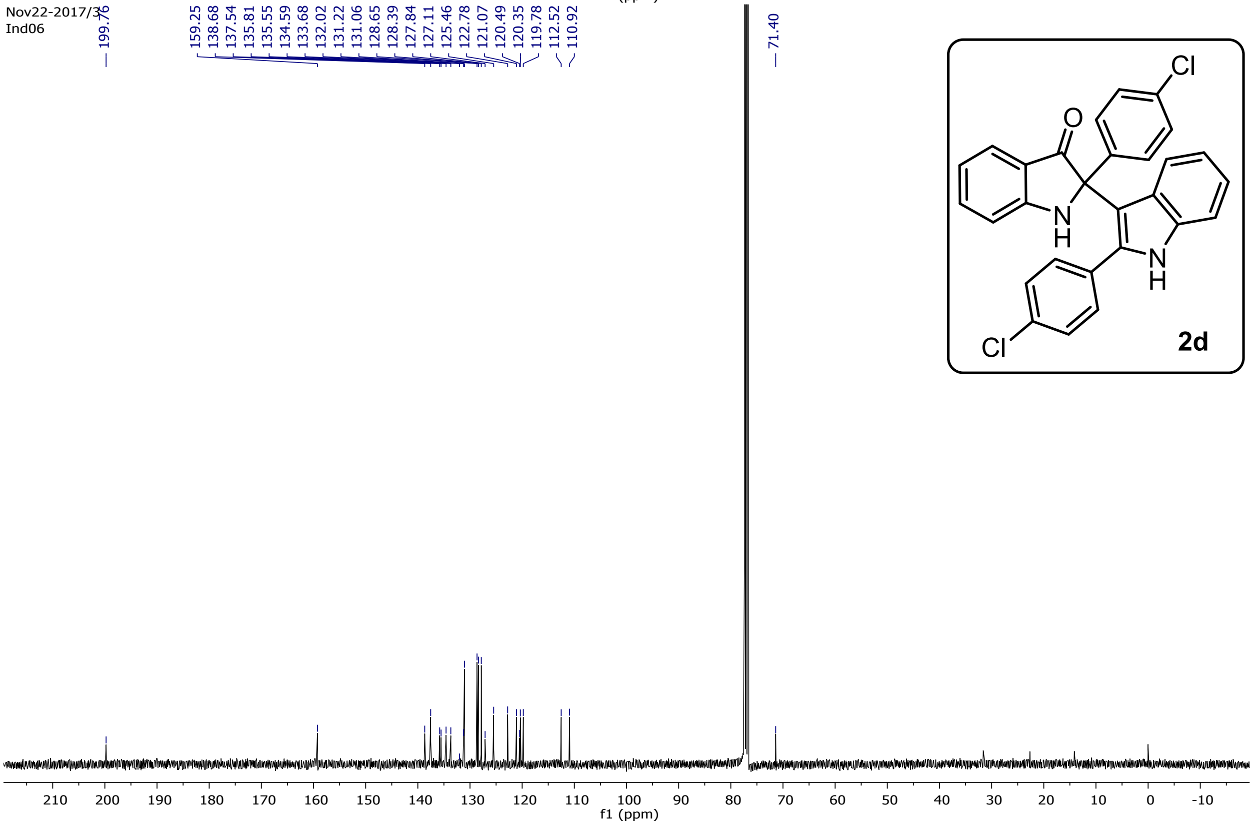
Nov22-2017/3
Ind06
199.76
159.25 138.68 137.54 135.81 135.55 134.59 133.68 132.02 131.22 131.06 128.65 128.39 127.84 127.11 125.46 122.78 121.07 120.49 120.35 119.78 112.52 110.92
71.40
f1 (ppm)
Cl
O
N
H
Cl
2d

Qualitative Compound Report

**Data File**	IND 06.d	**Sample Name**	IND 06
**Sample Type**	Sample	**Position**	P2-D6
**Instrument Name**	Instrument 1	**User Name**	
**Acq Method**	water_meoh_grad_6min_reg.m	**Acquired Time**	1/5/2018 11:47:59 AM
**IRM Calibration Status**	Success	**DA Method**	PROCESSNEW.m
**Comment**			

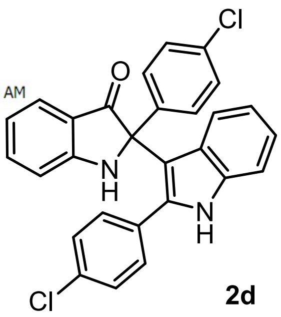

2d

**Sample Group**		**Info.**	
**Stream Name**	LC 1	**Acquisition SW Version**	6200 series TOF/6500 series Q-TOF B.06.01 (B6172 SP1)

Compound Table

Compound Label	RT	Mass	Abund	Formula	Tgt Mass	Diff (ppm)
Cpd 1: 3.288 491.0677	3.288	468.0777	95804	C28 H18 Cl2 N2 O	468.0796	-4.02

Compound Label	*m/z*	RT	Algorithm	Mass
Cpd 1: 3.288 491.0677	491.0677	3.288	Find By Formula	468.0777

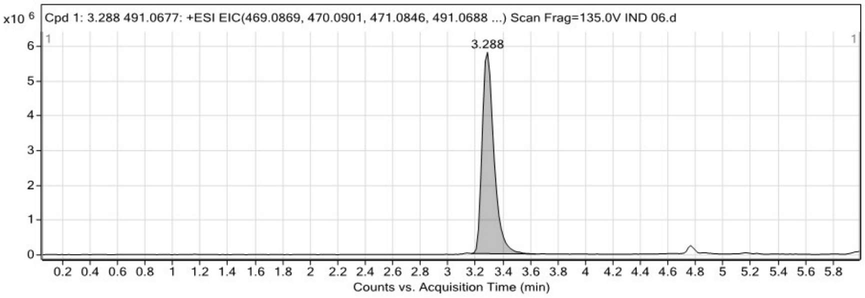

MS Zoomed Spectrum

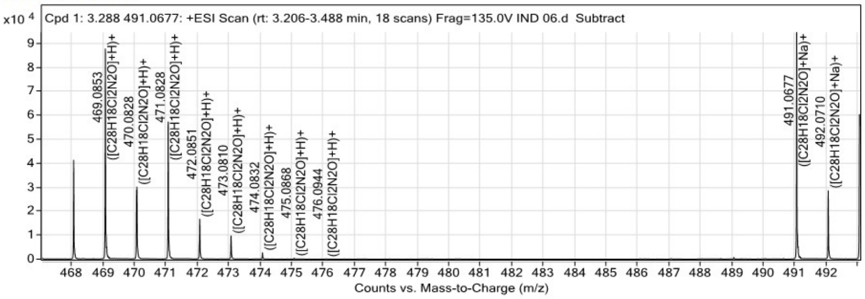

MS Spectrum Peak List

m/z	*Calc m/z*	Diff(ppm)	z	Abund	Formula	Ion
469.0853	469.0869	3.31	1	90402.71	C28H18Cl2N2O	(M+H)+
470.0828	470.0901	15.59	1	30174.06	C28H18Cl2N2O	(M+H)+

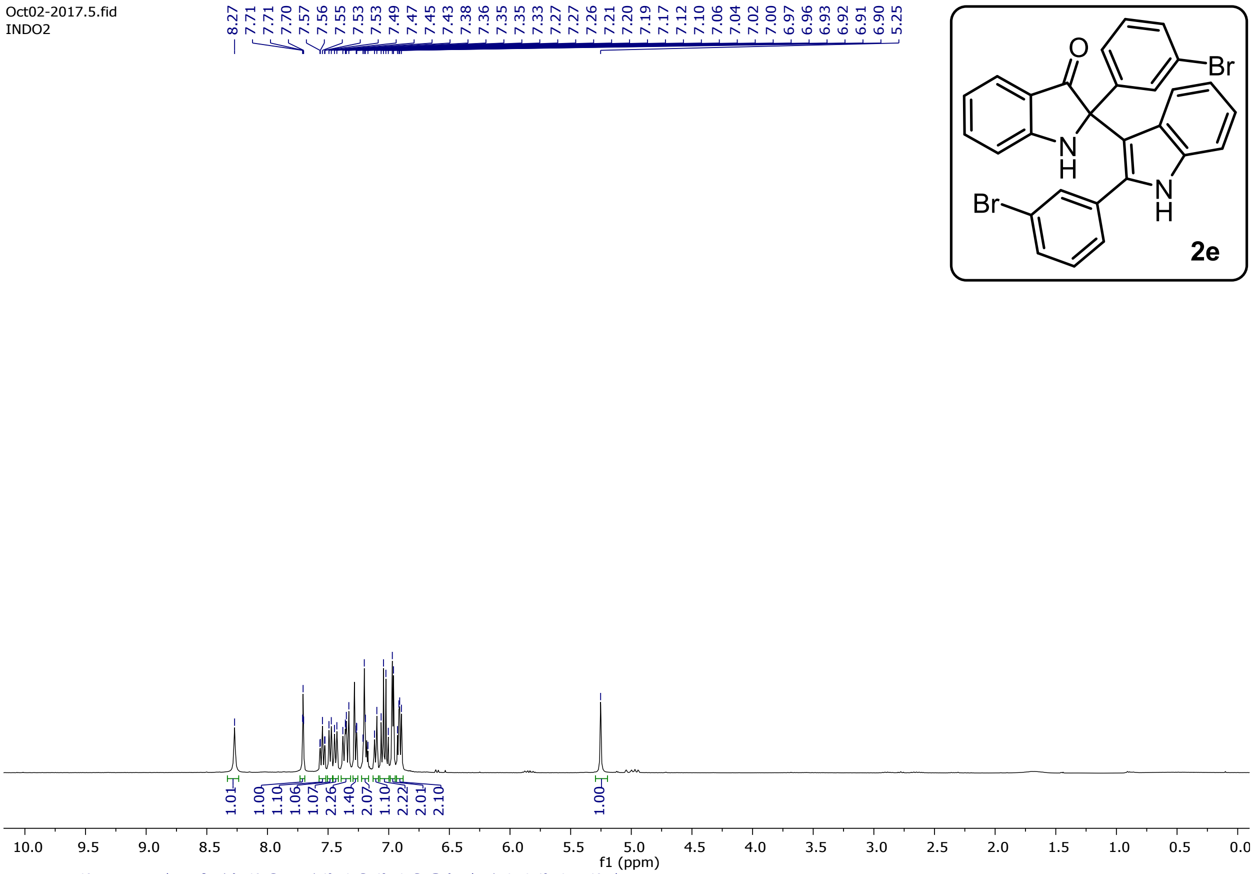
Oct02-2017.5.fid
INDO2
8.27 7.71 7.71 7.70 7.57 7.56 7.55 7.53 7.53 7.49 7.47 7.45 7.43 7.38 7.36 7.35 7.35 7.33 7.27 7.27 7.26 7.21 7.20 7.19 7.17 7.12 7.10 7.06 7.04 7.02 7.00 6.97 6.96 6.93 6.92 6.91 6.90 5.25
1.01 1.00 1.10 1.06 1.07 2.26 1.40 2.07 1.10 2.22 2.01 2.10 1.00
O
Br
N
H
Br
N
H
2e
f1 (ppm)

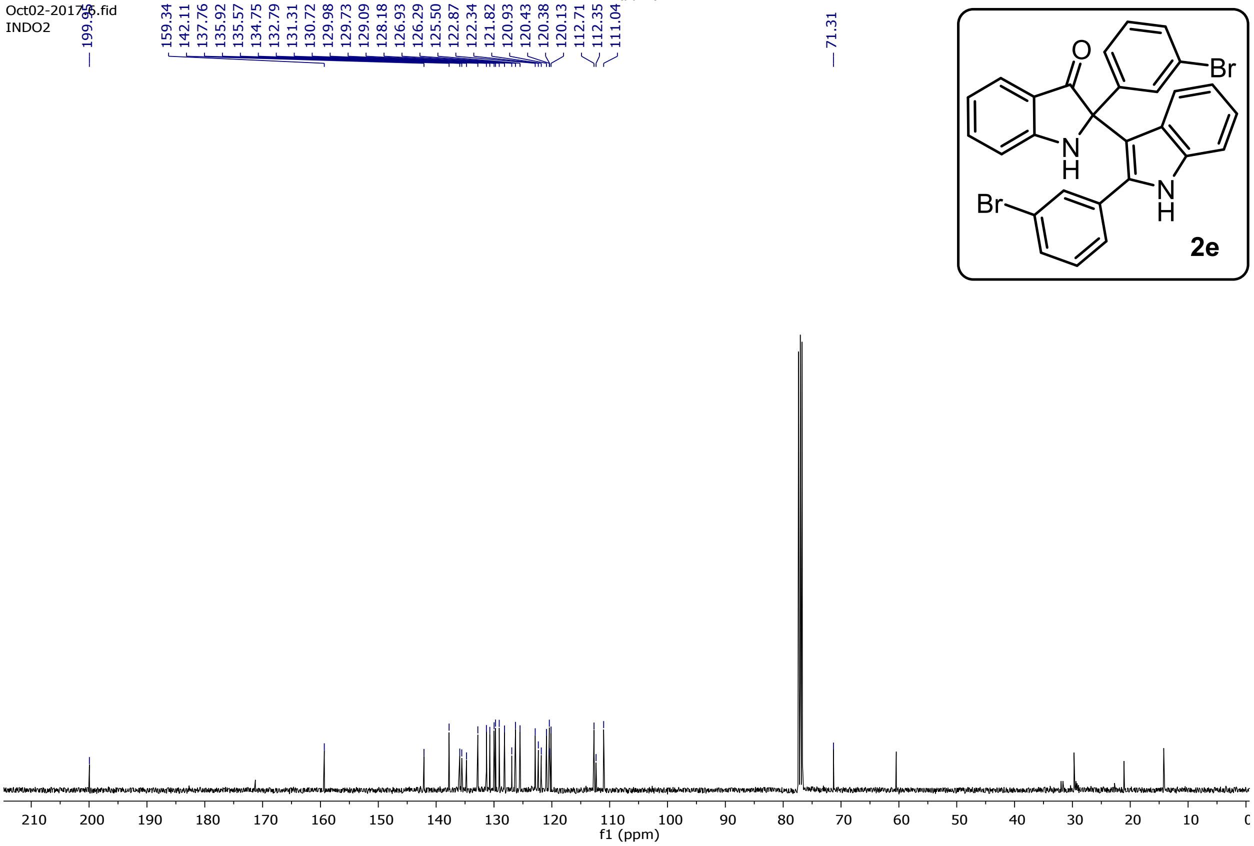
Oct02-2017.6.fid
INDO2
199.95 159.34 142.11 137.76 135.92 135.57 134.75 132.79 131.31 130.72 129.98 129.73 129.09 128.18 126.93 126.29 125.50 122.87 122.34 121.82 120.93 120.43 120.38 120.13 112.71 112.35 111.04 71.31
O
Br
N
H
Br
N
H
2e
f1 (ppm)

Qualitative Compound Report

**Data File**	IND 02.d	**Sample Name**	IND 02
**Sample Type**	Sample	**Position**	P2-D4
**Instrument Name**	Instrument 1	**User Name**	
**Acq Method**	water_meoh_grad_6min_reg.m	**Acquired Time**	1/5/2018 11:19:44 AM
**IRM Calibration Status**	Success	**DA Method**	PROCESSNEW.m
**Comment**			

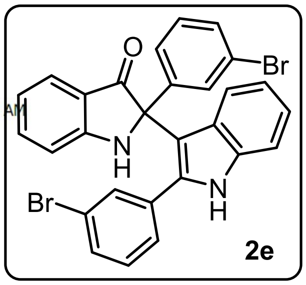

2e

**Sample Group**		**Info.**	
**Stream Name**	LC 1	**Acquisition SW Version**	6200 series TOF/6500 series Q-TOF B.06.01 (B6172 SP1)

Compound Table

Compound Label	RT	Mass	Abund	Formula	Tgt Mass	Diff (ppm)
Cpd 1: 3.401 558.9818	3.401	555.9761	74707	C28 H18 Br2 N2 O	555.9786	-4.52

Compound Label	*m/z*	RT	Algorithm	Mass
Cpd 1: 3.401 558.9818	580.9638	3.401	Find By Formula	555.9761

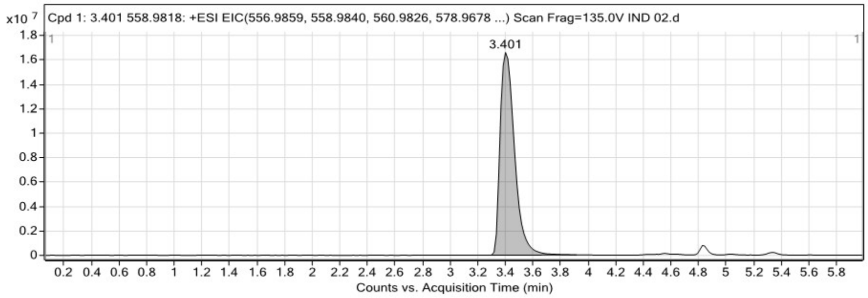

MS Zoomed Spectrum

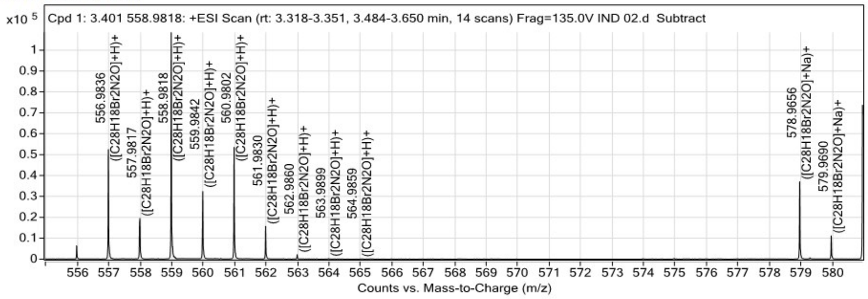

MS Spectrum Peak List

m/z	*Calc m/z*	Diff(ppm)	z	Abund	Formula	Ion
556.9836	556.9859	3.98	1	53488.55	C28H18Br2N2O	(M+H)+
557.9817	557.9891	13.22	1	19551.57	C28H18Br2N2O	(M+H)+

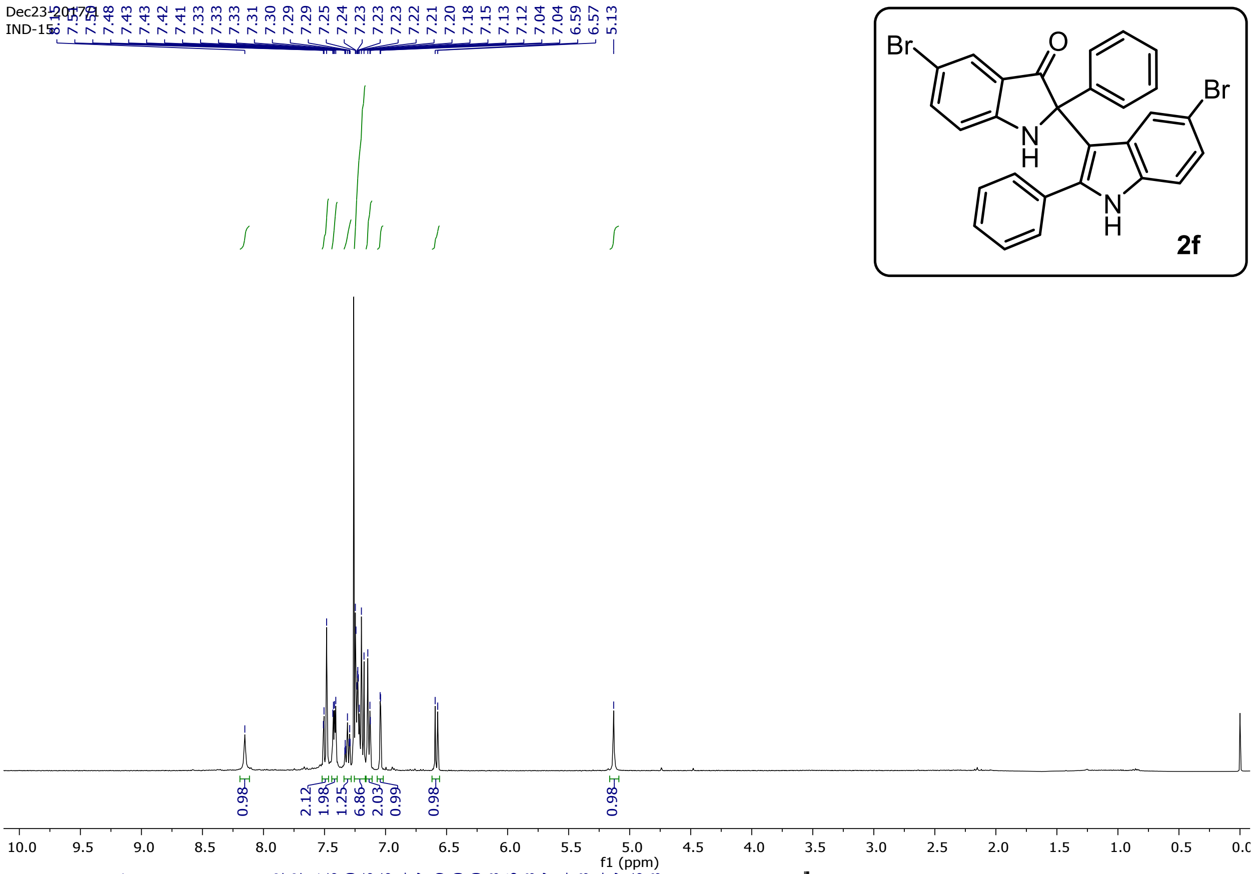

Dec23-2017/1
IND-15
7.48
7.43
7.43
7.42
7.41
7.33
7.33
7.33
7.31
7.30
7.29
7.29
7.25
7.24
7.23
7.23
7.23
7.22
7.21
7.20
7.18
7.15
7.13
7.12
7.04
7.04
6.59
6.57
5.13
Br
O
Br
N
H
N
H
2f
0.98
2.12
1.98
1.25
6.86
2.03
0.99
0.98
0.98
10.0 9.5 9.0 8.5 8.0 7.5 7.0 6.5 6.0 5.5 5.0 4.5 4.0 3.5 3.0 2.5 2.0 1.5 1.0 0.5
f1 (ppm)

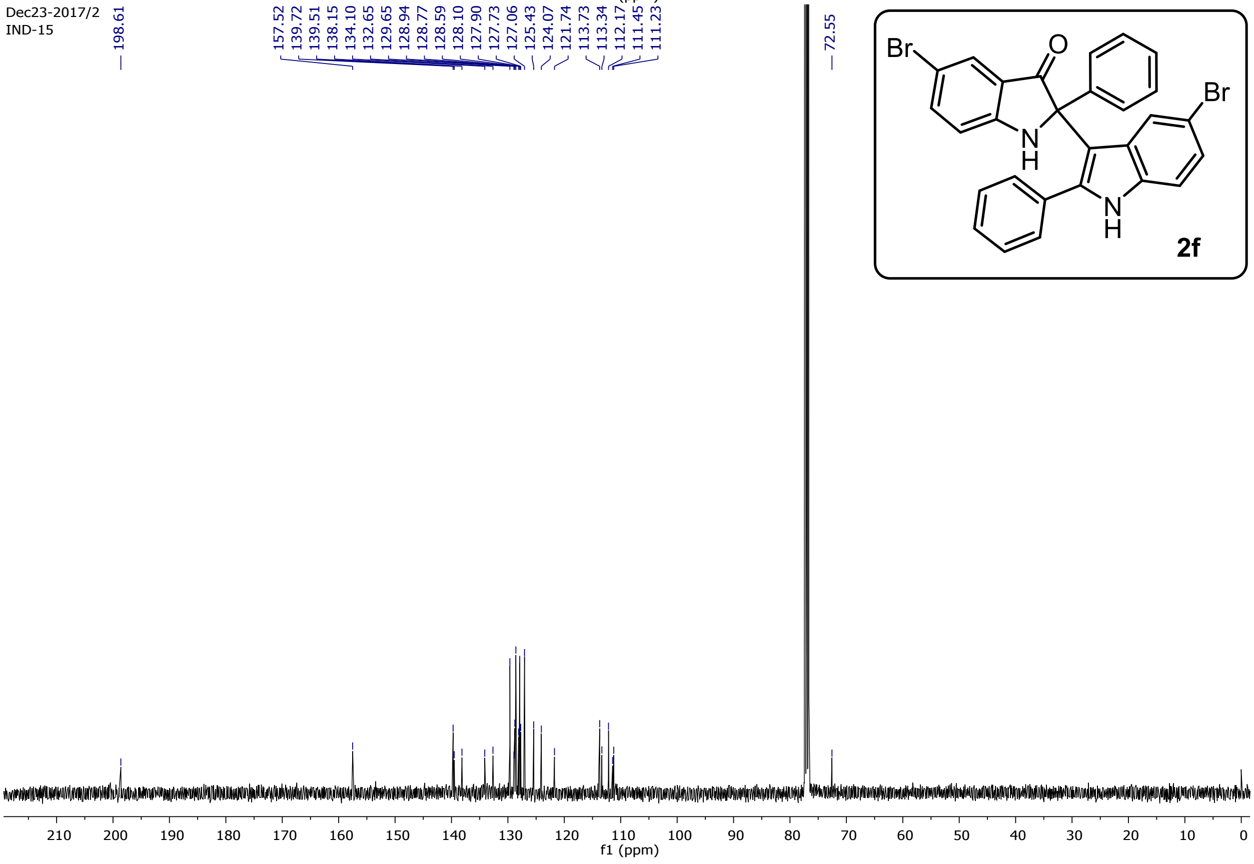

Dec23-2017/2
IND-15
198.61
157.52
139.72
139.51
138.15
134.10
132.65
129.65
128.94
128.77
128.59
128.10
127.90
127.73
127.06
125.43
124.07
121.74
113.73
113.34
112.17
111.45
111.23
72.55
Br
O
Br
N
H
N
H
2f
210 200 190 180 170 160 150 140 130 120 110 100 90 80 70 60 50 40 30 20 10 0
f1 (ppm)

Qualitative Compound Report

**Data File**	INd 15.d	**Sample Name**	IND 15
**Sample Type**	Sample	**Position**	P2-E1
**Instrument Name**	Instrument 1	**User Name**	
**Acq Method**	water_meoh_grad_6min_reg.m	**Acquired Time**	1/5/2018 1:[illegible]:57 PM
**IRM Calibration Status**	Success	**DA Method**	PROCESSNEW.m
**Comment**			

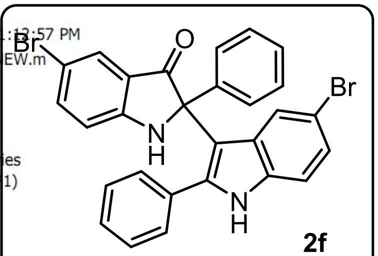

2f

**Sample Group**		**Info.**	
**Stream Name**	LC 1	**Acquisition SW Version**	6200 series TOF/6500 series Q-TOF B.06.01 (B6172 SP1)

Compound Table

Compound Label	RT	Mass	Abund	Formula	Tgt Mass	Diff (ppm)
Cpd 1: 3.588 558.9818	3.588	555.9761	114294	C28 H18 Br2 N2 O	555.9786	-4.51

Compound Label	*m/z*	RT	Algorithm	Mass
Cpd 1: 3.588 558.9818	580.9638	3.588	Find By Formula	555.9761

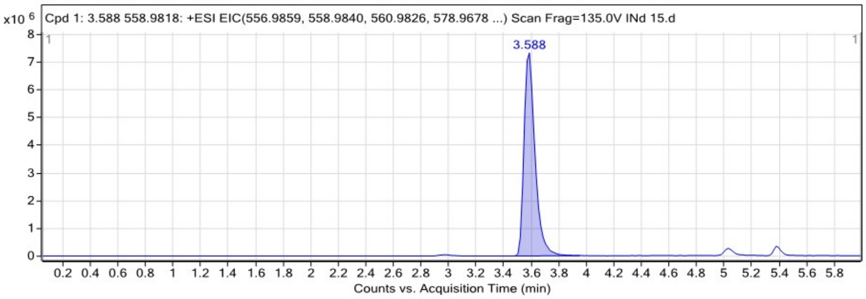

MS Zoomed Spectrum

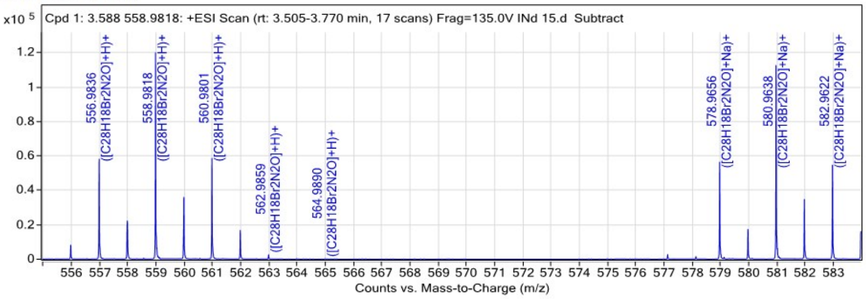

MS Spectrum Peak List

m/z	*Calc m/z*	Diff(ppm)	z	Abund	Formula	Ion
556.9836	556.9859	4.07	1	59351.38	C28H18Br2N2O	(M+H)+
557.9813	557.9891	14.05	1	22361.84	C28H18Br2N2O	(M+H)+

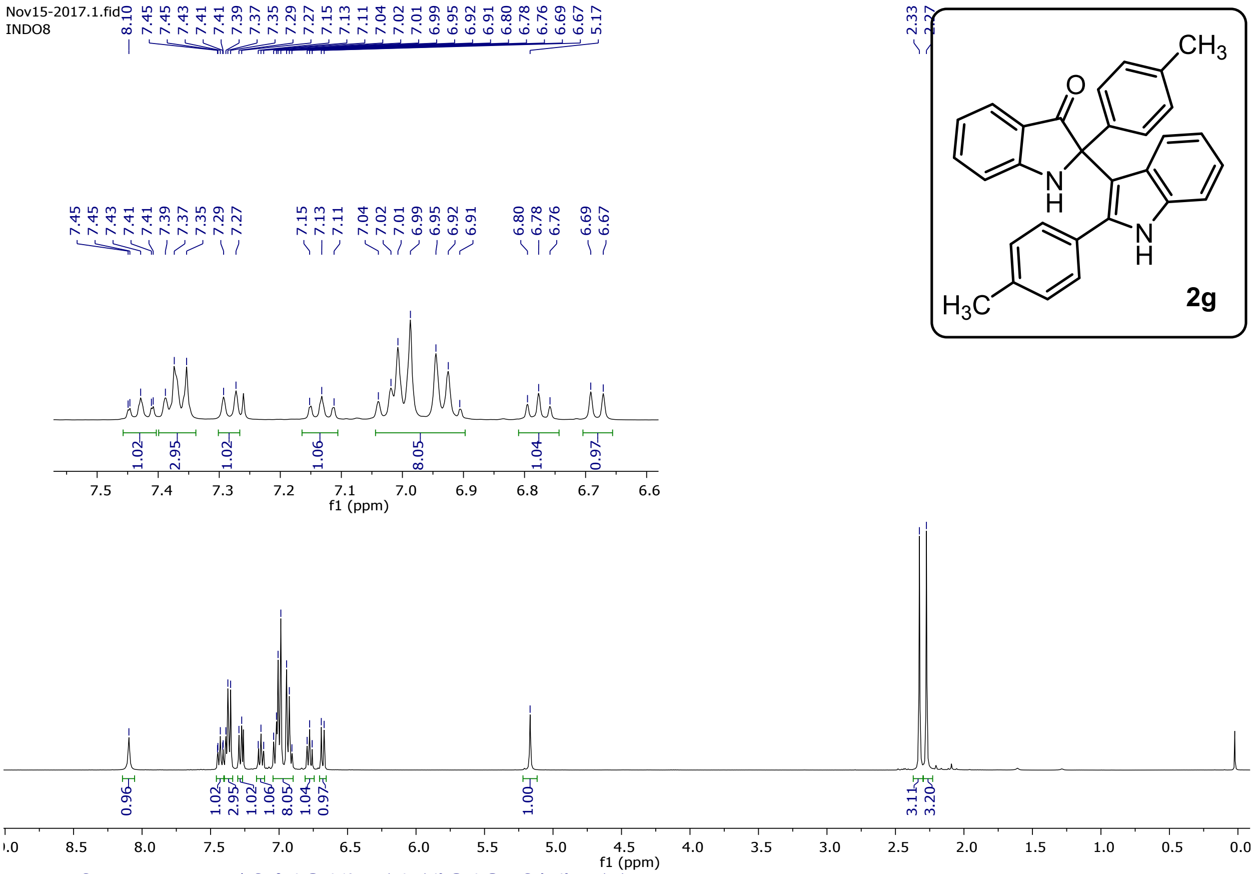
Nov15-2017.1.fid
INDO8
2g
f1 (ppm)

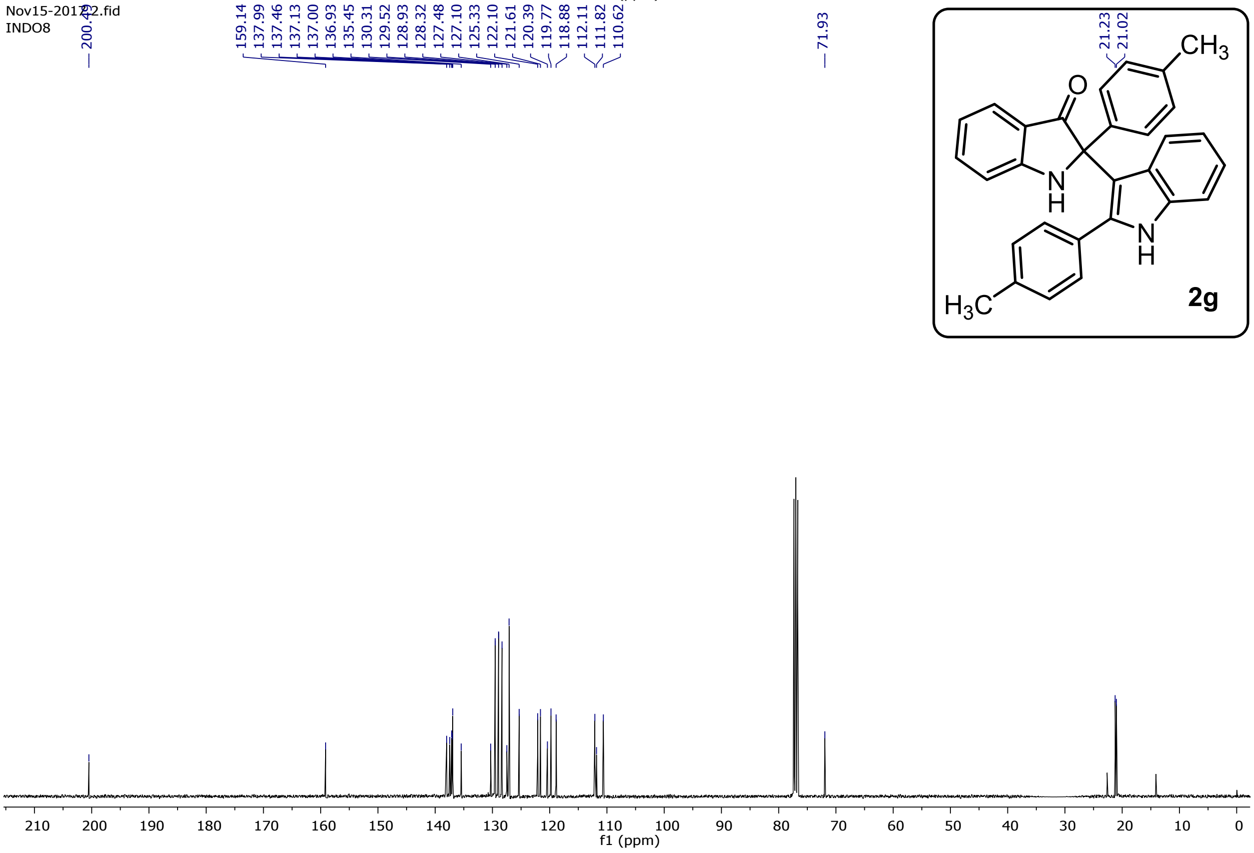
INDO8
2g
f1 (ppm)

Qualitative Compound Report

**Data File**	IND 08.d	**Sample Name**	IND 08
**Sample Type**	Sample	**Position**	P2-D7
**Instrument Name**	Instrument 1	**User Name**	
**Acq Method**	water_meoh_grad_6min_reg.m	**Acquired Time**	1/5/2018 12:02:06 PM
**IRM Calibration Status**	Success	**DA Method**	PROCESSNEW.m
**Comment**			

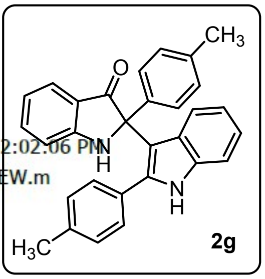

2g

**Sample Group**		**Info.**	
**Stream Name**	LC 1	**Acquisition SW Version**	6200 series TOF/6500 series Q-TOF B.06.01 (B6172 SP1)

Compound Table

Compound Label	RT	Mass	Abund	Formula	Tgt Mass	Diff (ppm)
Cpd 1: 3.549 353.1272	3.549	428.1868	59	C30 H24 N2 O	428.1889	-4.9

Compound Label	*m/z*	RT	Algorithm	Mass
Cpd 1: 3.549 353.1272	451.176	3.549	Find By Formula	428.1868

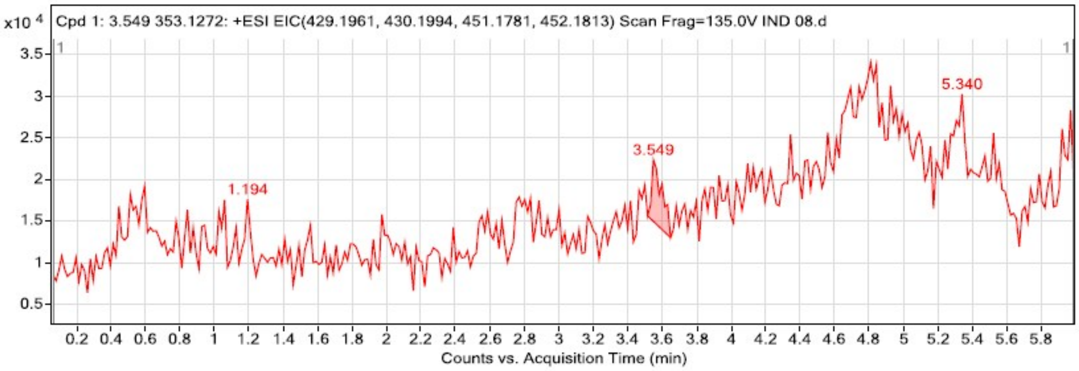

MS Zoomed Spectrum

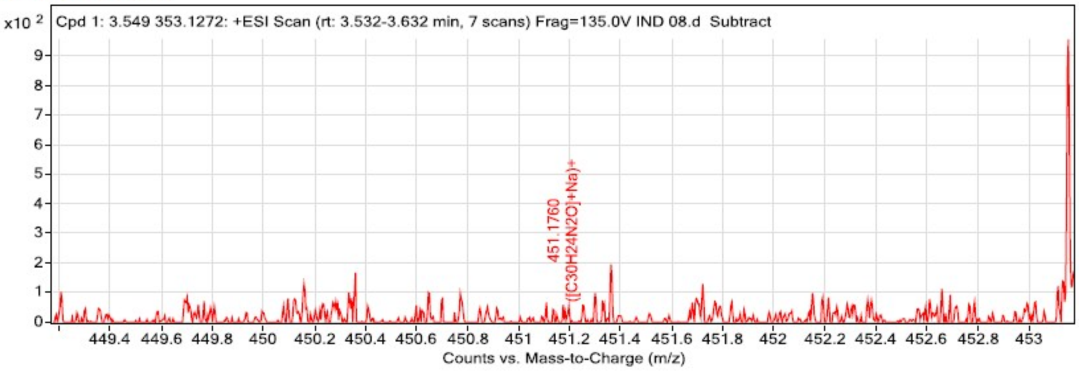

MS Spectrum Peak List

m/z	*Calc m/z*	Diff(ppm)	z	Abund	Formula	Ion
451.176	451.1781	4.66	1	59.44	C30H24N2O	(M+Na)+

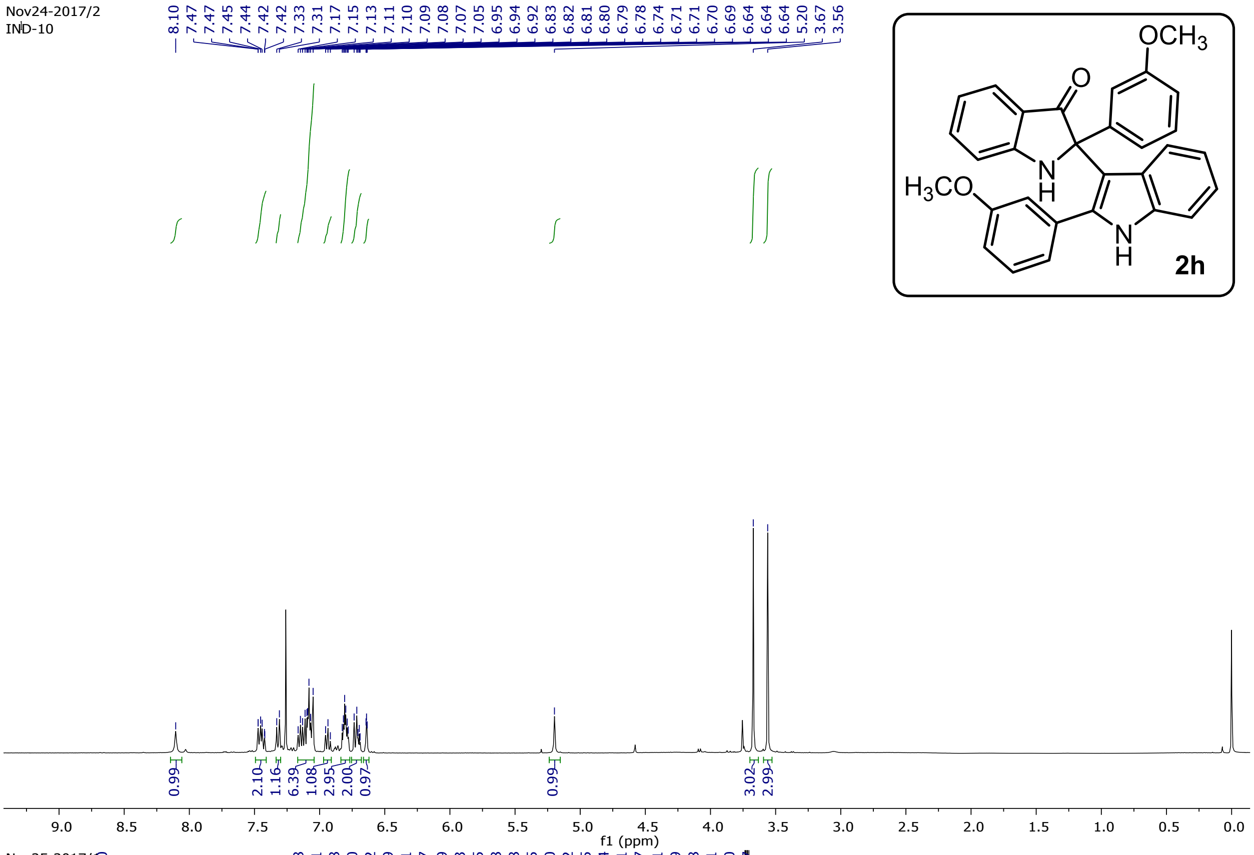
Nov24-2017/2
IND-10
8.10 7.47 7.47 7.45 7.44 7.42 7.42 7.33 7.31 7.17 7.15 7.13 7.11 7.10 7.09 7.08 7.07 7.05 6.95 6.94 6.92 6.83 6.82 6.81 6.80 6.79 6.78 6.74 6.71 6.71 6.70 6.69 6.64 6.64 6.64 5.20 3.67 3.56
0.99 2.10 1.16 6.39 1.08 2.95 2.00 0.97 0.99 3.02 2.99
9.0 8.5 8.0 7.5 7.0 6.5 6.0 5.5 5.0 4.5 4.0 3.5 3.0 2.5 2.0 1.5 1.0 0.5 0.0
f1 (ppm)
OCH3
O
N
H
H3CO
N
H
2h

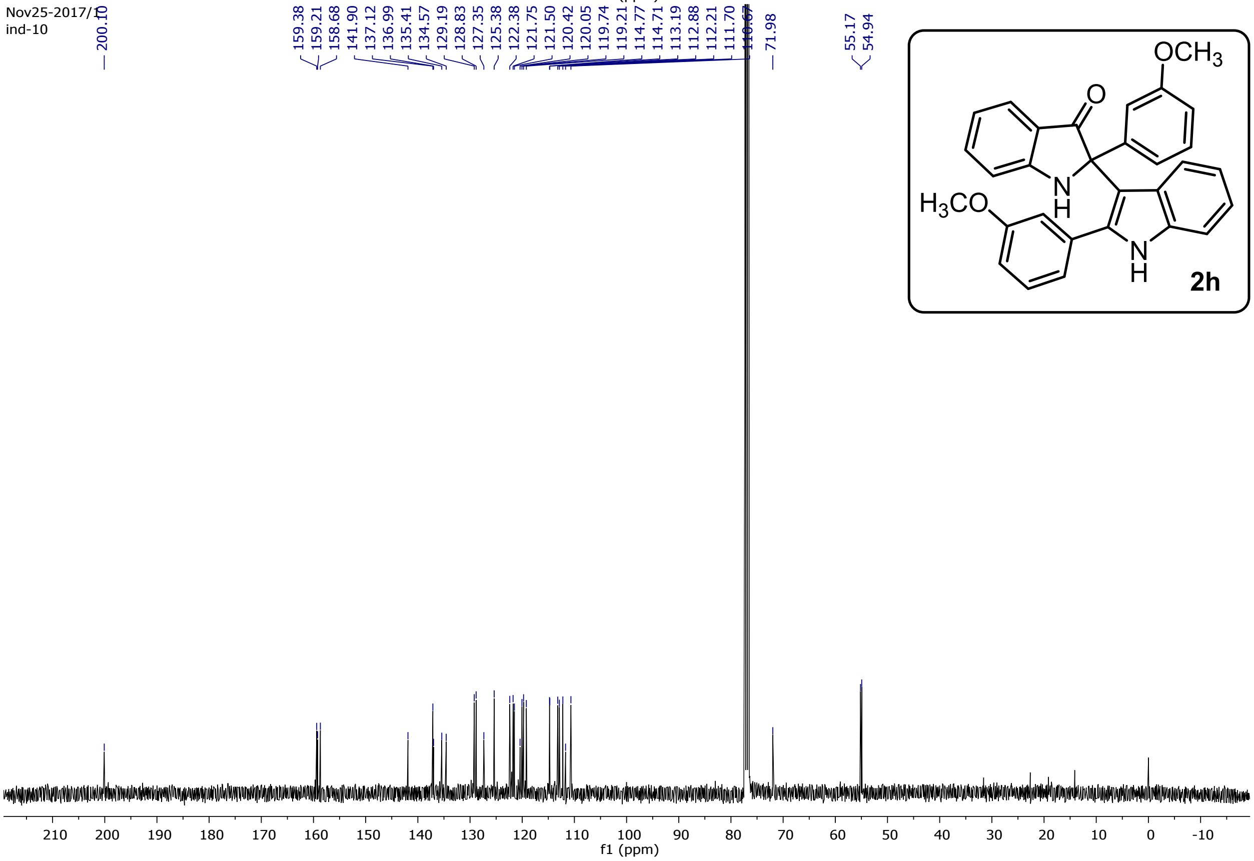
Nov25-2017/10
ind-10
200.10
159.38 159.21 158.68 141.90 137.12 136.99 135.41 134.57 129.19 128.83 127.35 125.38 122.38 121.75 121.50 120.42 120.05 119.74 119.21 114.77 114.71 113.19 112.88 112.21 111.70 110.67 71.98
55.17 54.94
210 200 190 180 170 160 150 140 130 120 110 100 90 80 70 60 50 40 30 20 10 0 -10
f1 (ppm)
OCH3
O
N
H
H3CO
N
H
2h

Qualitative Compound Report

**Data File**	IND 10.d	**Sample Name**	IND 10
**Sample Type**	Sample	**Position**	P2-D9
**Instrument Name**	Instrument 1	**User Name**	
**Acq Method**	water_meoh_grad_6min_reg.m	**Acquired Time**	1/5/2018 12:30:30 PM
**IRM Calibration Status**	Success	**DA Method**	PROCESSNEW.m
**Comment**			

**Sample Group**		**Info.**	
**Stream Name**	LC 1	**Acquisition SW Version**	6200 series TOF/6500 series Q-TOF B.06.01 (B6172 SP1)

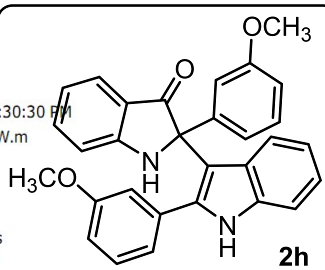

2h

Compound Table

Compound Label	RT	Mass	Abund	Formula	Tgt Mass	Diff (ppm)
Cpd 1: 1.437 483.1666	1.437	460.1773	155498	C30 H24 N2 O3	460.1787	-3.11

Compound Label	*m/z*	RT	Algorithm	Mass
Cpd 1: 1.437 483.1666	483.1666	1.437	Find By Formula	460.1773

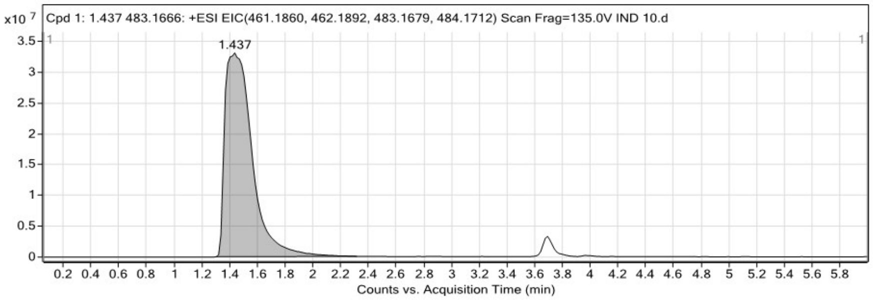

MS Zoomed Spectrum

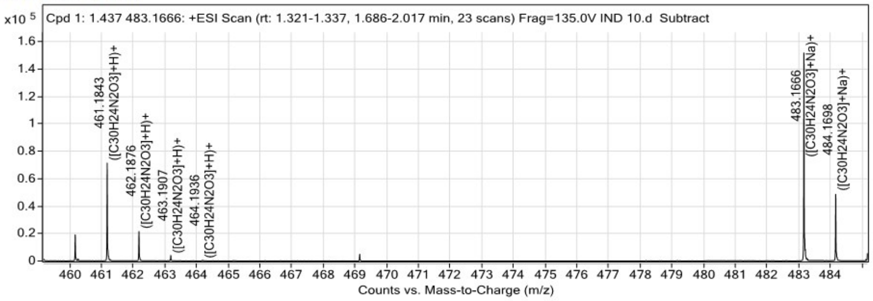

MS Spectrum Peak List

m/z	*Calc m/z*	Diff(ppm)	z	Abund	Formula	Ion
461.1843	461.186	3.58	1	71739.17	C30H24N2O3	(M+H)+
462.1876	462.1892	3.42	1	22086.86	C30H24N2O3	(M+H)+

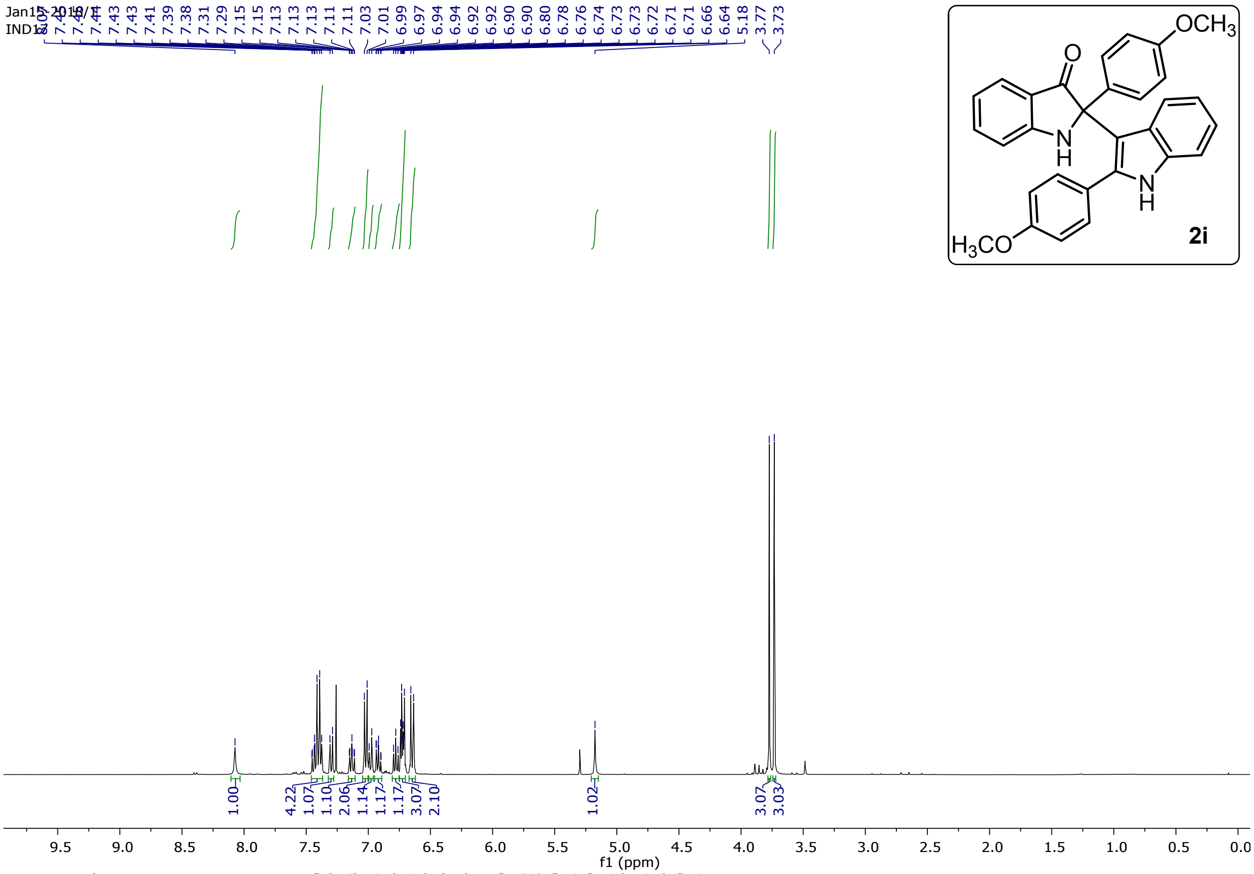
Jan15-2018/1
IND17
7.43 7.43 7.41 7.39 7.38 7.31 7.29 7.15 7.15 7.13 7.13 7.13 7.11 7.11 7.03 7.01 6.99 6.97 6.94 6.94 6.92 6.92 6.90 6.90 6.80 6.78 6.76 6.74 6.73 6.73 6.72 6.71 6.71 6.66 6.64 5.18 3.77 3.73
1.00 4.22 1.07 1.10 2.06 1.14 1.17 1.17 3.07 2.10 1.02 3.07 3.03
9.5 9.0 8.5 8.0 7.5 7.0 6.5 6.0 5.5 5.0 4.5 4.0 3.5 3.0 2.5 2.0 1.5 1.0 0.5 0.0
f1 (ppm)
OCH3
O
N H
N H
H3CO
2i

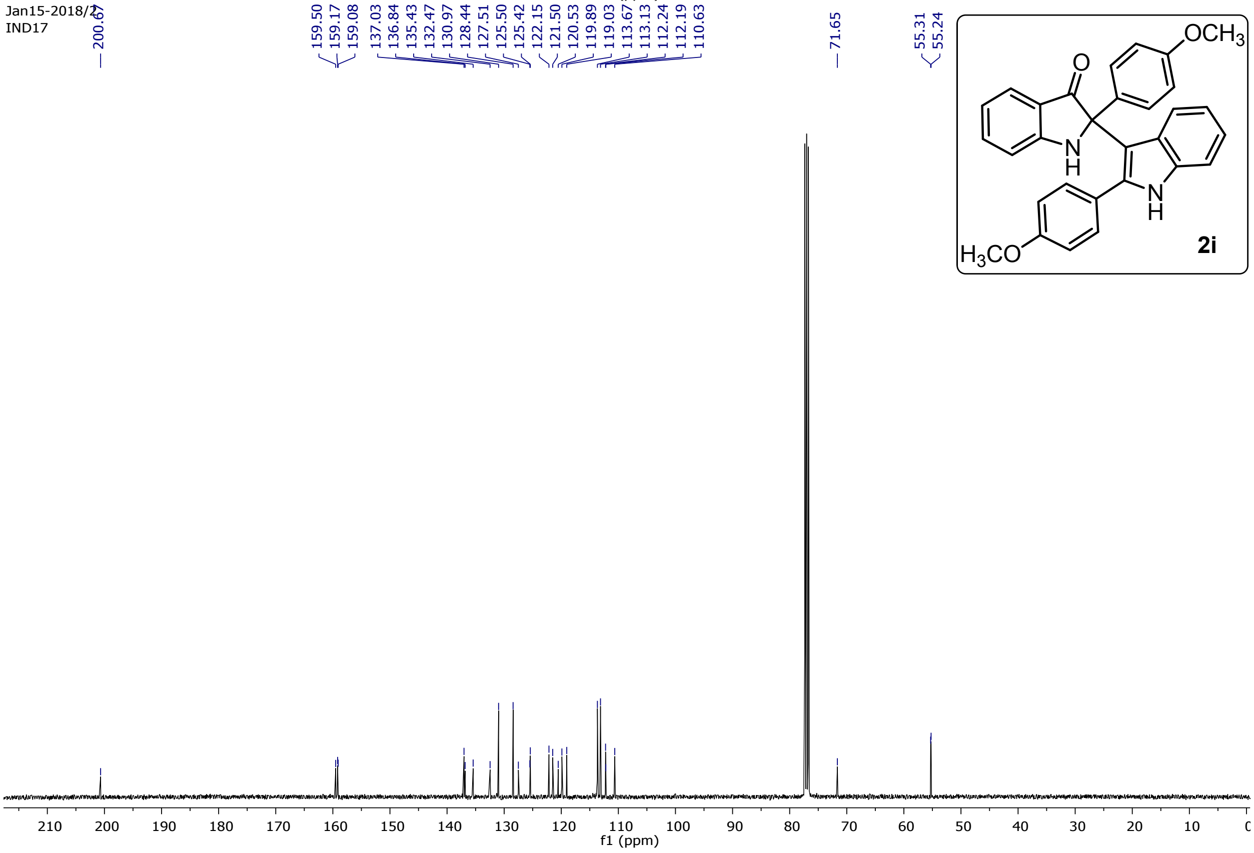
Jan15-2018/2
IND17
200.67
159.50 159.17 159.08 137.03 136.84 135.43 132.47 130.97 128.44 127.51 125.50 125.42 122.15 121.50 120.53 119.89 119.03 113.67 113.13 112.24 112.19 110.63
71.65
55.31 55.24
210 200 190 180 170 160 150 140 130 120 110 100 90 80 70 60 50 40 30 20 10
f1 (ppm)
OCH3
O
N H
N H
H3CO
2i

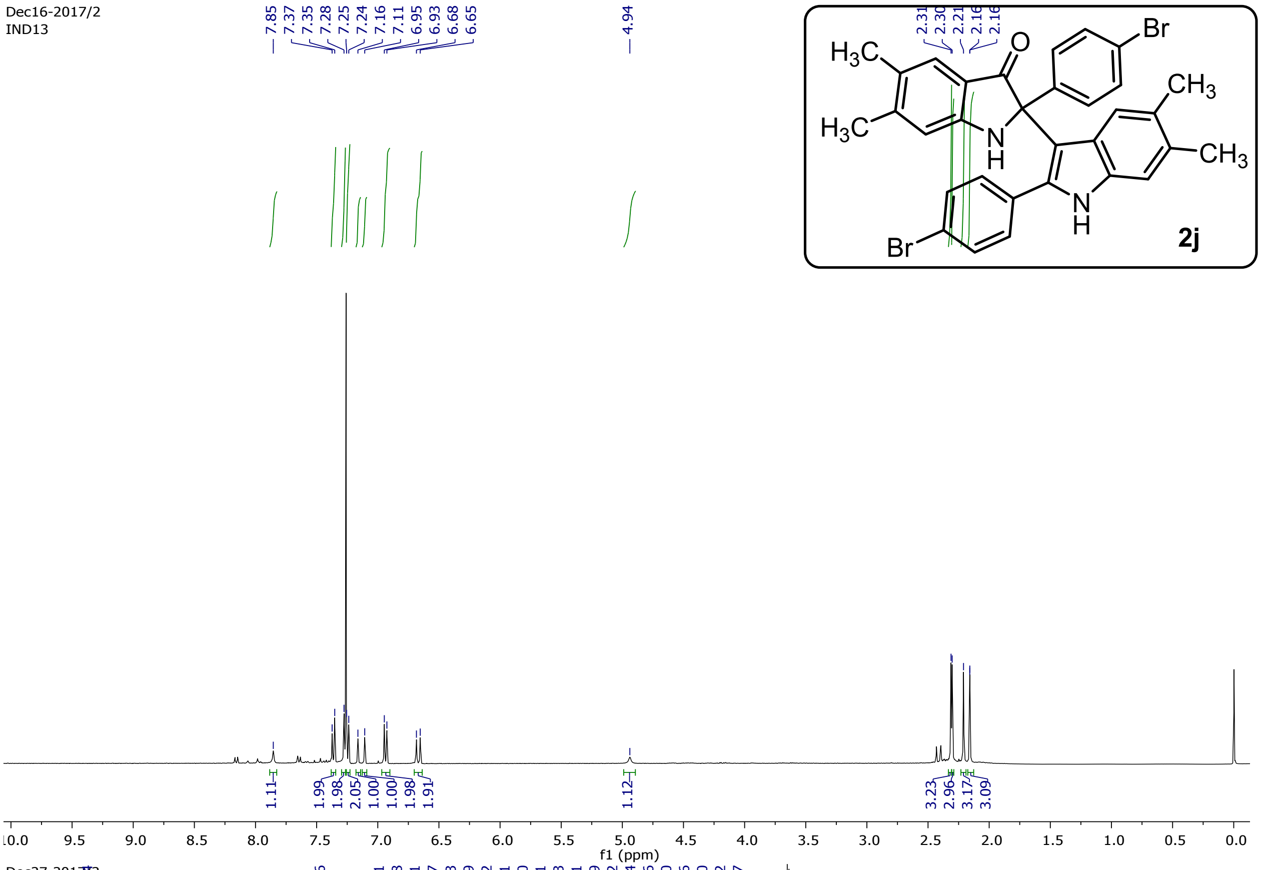
Dec16-2017/2
IND13
7.85
7.37
7.35
7.28
7.25
7.24
7.16
7.11
6.95
6.93
6.68
6.65
4.94
2.31
2.30
2.21
2.16
2.16
1.11
1.99
1.98
2.05
1.00
1.00
1.98
1.91
1.12
3.23
2.96
3.17
3.09
f1 (ppm)
Br
O
H_3C
CH_3
N
H
Br
2j

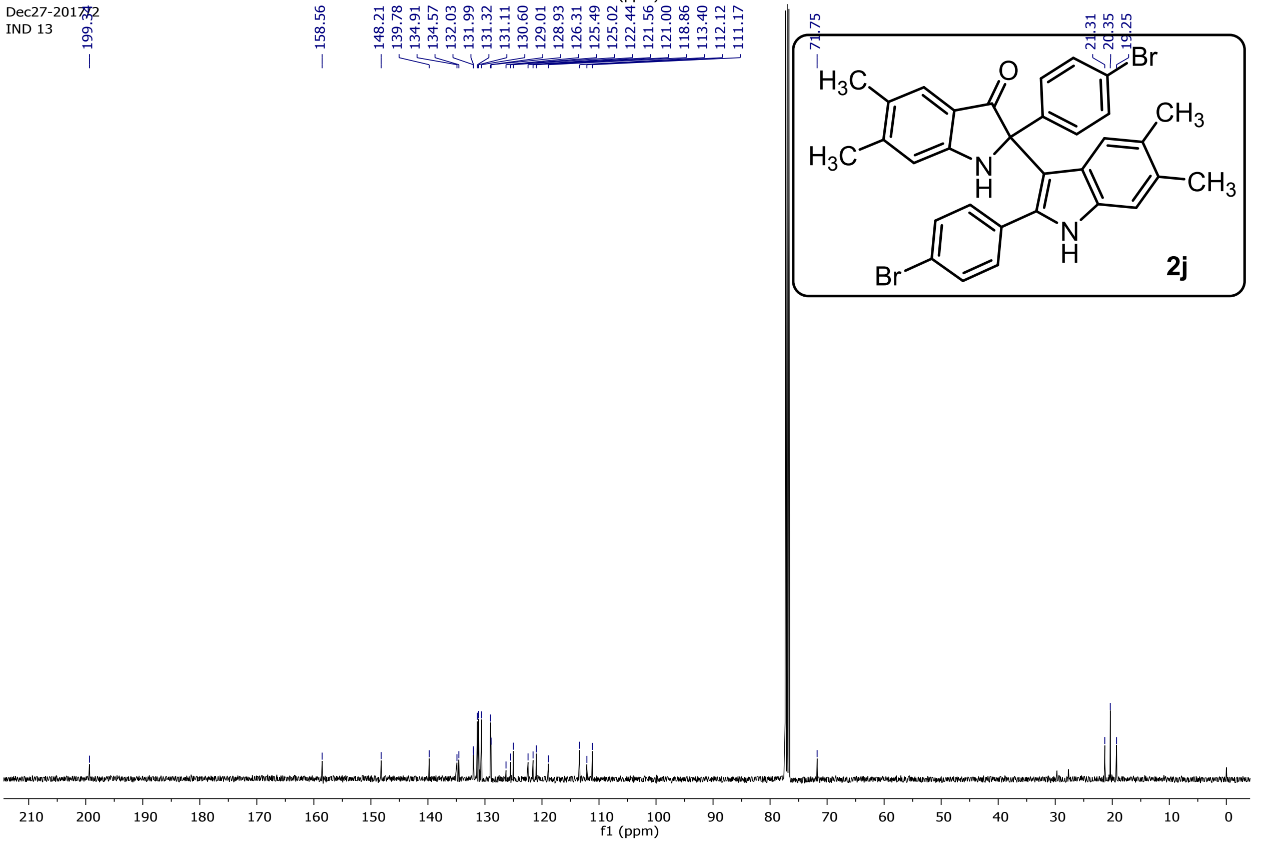
Dec27-2017/2
IND 13
199.34
158.56
148.21
139.78
134.91
134.57
132.03
131.99
131.32
131.11
130.60
129.01
128.93
126.31
125.49
125.02
122.44
121.56
121.00
118.86
113.40
112.12
111.17
71.75
21.31
20.35
19.25
f1 (ppm)
O
Br
H_3C
CH_3
N
H
Br
2j

Qualitative Compound Report

**Data File**	IND 13.d	**Sample Name**	IND 13
**Sample Type**	Sample	**Position**	P1-[illegible]
**Instrument Name**	Instrument 1	**User Name**	
**Acq Method**	water_meoh_grad_6min_reg.m	**Acquired Time**	1/5/2018 1[illegible]:58:47 PM
**IRM Calibration Status**	Success	**DA Method**	[illegible]PROCESSNEW.m
**Comment**			

**Sample Group**		**Info.**	
**Stream Name**	LC 1	**Acquisition SW Version**	6200 series TOF/6500 series Q-TOF B.06.01 (B6172 SP1)

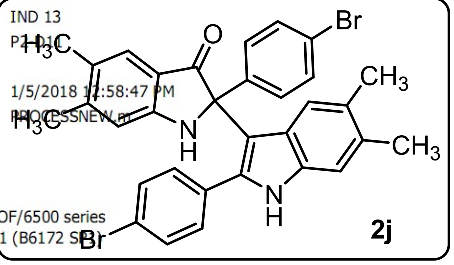

2j

Compound Table

Compound Label	RT	Mass	Abund	Formula	Tgt Mass	Diff (ppm)
Cpd 1: 4.581 610.1825	4.581	612.038	29875	C32 H26 Br2 N2 O	612.0412	-5.27

Compound Label	*m/z*	RT	Algorithm	Mass
Cpd 1: 4.581 610.1825	637.0269	4.581	Find By Formula	612.038

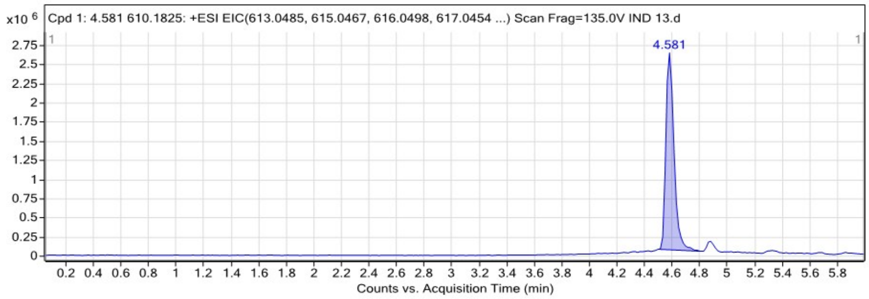

MS Zoomed Spectrum

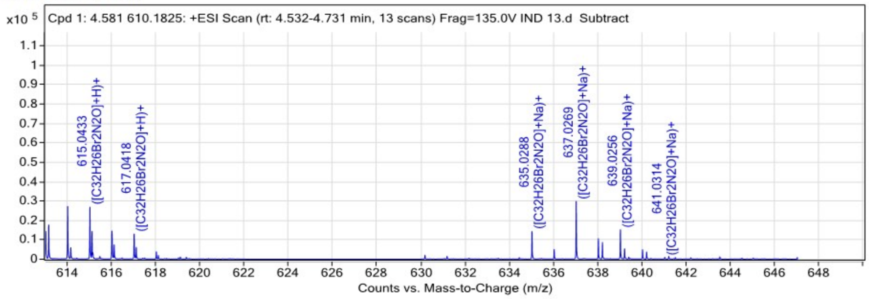

MS Spectrum Peak List

m/z	*Calc m/z*	Diff(ppm)	z	Abund	Formula	Ion
615.0433	615.0467	5.5	1	27156.81	C32H26Br2N2O	(M+H)+
616.0391	616.0498	17.24	1	14915.89	C32H26Br2N2O	(M+H)+

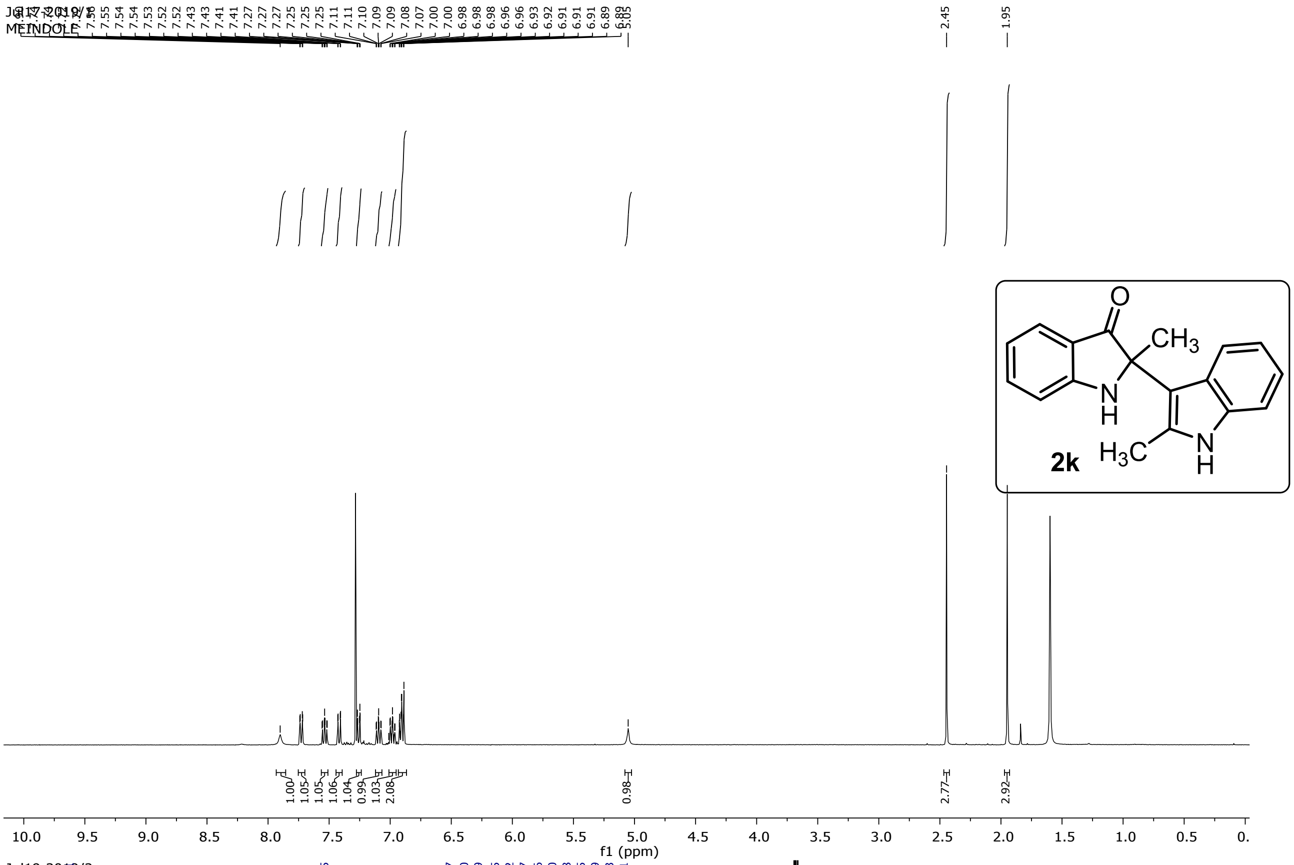
7.55
7.54
7.54
7.53
7.52
7.52
7.43
7.43
7.41
7.41
7.27
7.27
7.25
7.25
7.25
7.11
7.11
7.10
7.09
7.09
7.08
7.07
7.00
7.00
6.98
6.98
6.98
6.96
6.96
6.93
6.92
6.92
6.91
6.91
6.91
6.89
5.05
2.45
1.95
1.00
1.05
1.05
1.06
1.04
0.99
1.03
2.08
0.98
2.77
2.92
2k
O
CH3
N
H
H3C
N
H
10.0
9.5
9.0
8.5
8.0
7.5
7.0
6.5
6.0
5.5
5.0
4.5
4.0
3.5
3.0
2.5
2.0
1.5
1.0
0.5
f1 (ppm)
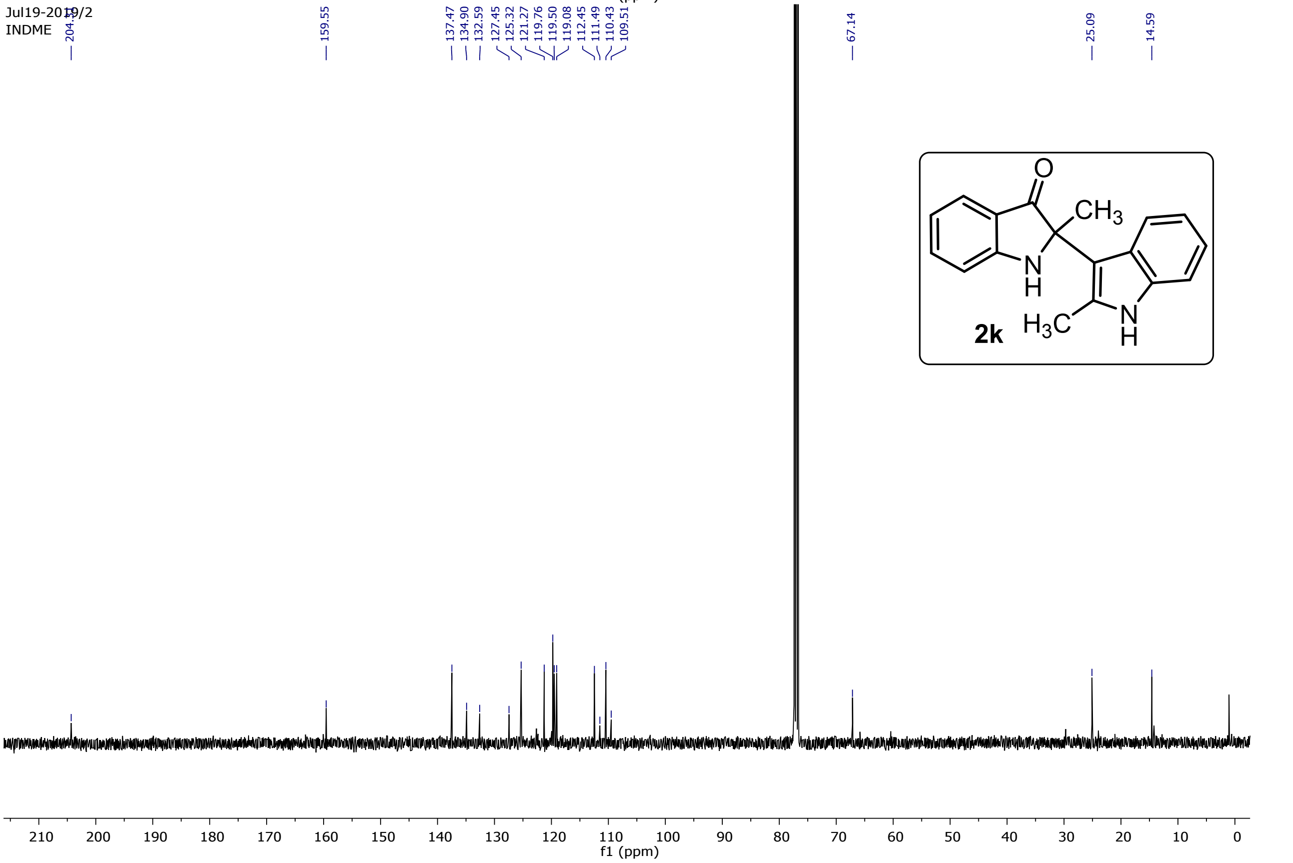
Jul19-2019/2
INDME
159.55
137.47
134.90
132.59
127.45
125.32
121.27
119.76
119.50
119.08
112.45
111.49
110.43
109.51
67.14
25.09
14.59
2k
O
CH3
N
H
H3C
N
H
210
200
190
180
170
160
150
140
130
120
110
100
90
80
70
60
50
40
30
20
10
0
f1 (ppm)

HRMS Facility, BITS Pilani, Pilani Campus
Compound Report

**Data File**	VS-INDD.d	**Sample Name**	VS-INDD
**Sample Type**	Sample	**Position**	P2-D1
**Instrument Name**	Instrument 1	**User Name**	
**Acq Method**	ChB60ChD40_isocratic_esi_positive_3min.bintu.m	**Acquired Time**	7/19/2019 5:34:30 PM
**IRM Calibration Status**	Success	**DA Method**	PROCESSNEW.m
**Comment**			

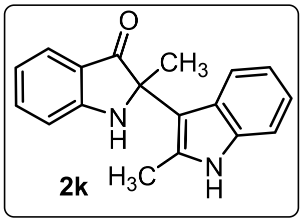

2k

**Sample Group**		**Info.**	
**Stream Name**	LC 1	**Acquisition SW Version**	6200 series TOF/6500 series Q-TOF B.06.01 (B6172 SP1)

Compound Table

Compound Label	RT	Mass	Abund	Formula	Tgt Mass	Diff (ppm)
Cpd 1: 0.191 404.2067	0.191	276.1262	36358	C18 H16 N2 O	276.1263	-0.27

Compound Label	*m/z*	RT	Algorithm	Mass
Cpd 1: 0.191 404.2067	277.1334	0.191	Find By Formula	276.1262

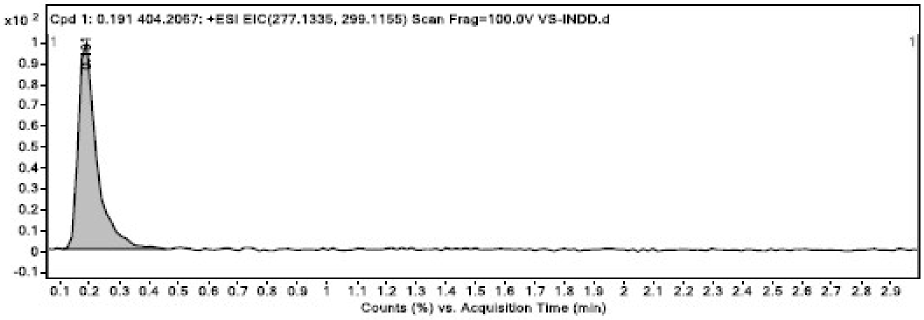

MS Zoomed Spectrum

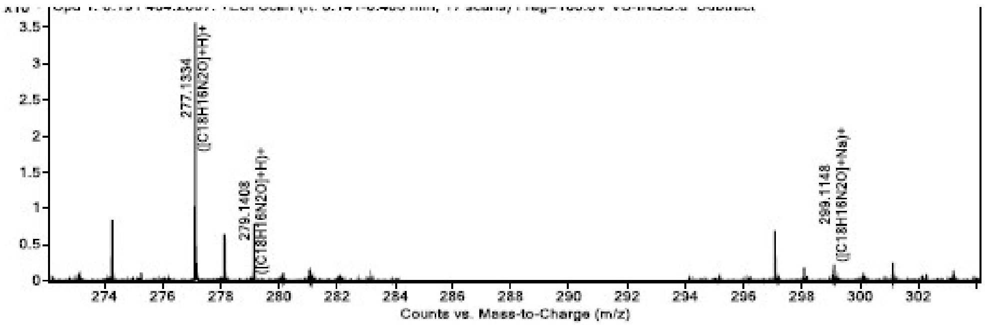

MS Spectrum Peak List

m/z	*Calc m/z*	Diff(ppm)	z	Abund	Formula	Ion
277.1334	277.1335	0.37	1	36358.38	C18H16N2O	(M+H)+
278.137	278.1367	-0.91	1	6537.04	C18H16N2O	(M+H)+
279.1408	279.1397	-4.27	1	596.3	C18H16N2O	(M+H)+
299.1148	299.1155	2.44	1	2278.87	C18H16N2O	(M+Na)+
300.1177	300.1186	3	1	478.05	C18H16N2O	(M+Na)+

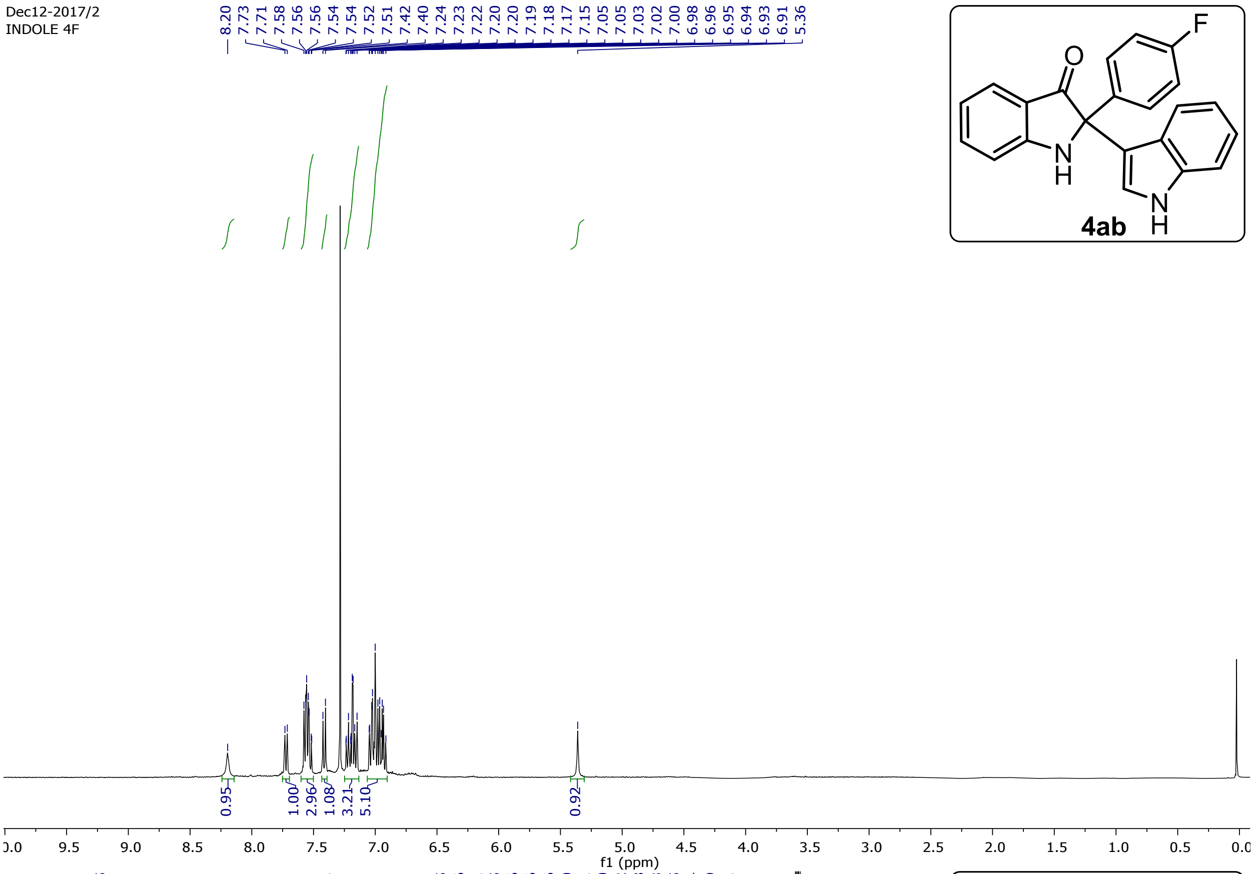
Dec12-2017/2
INDOLE 4F
8.20
7.73
7.71
7.58
7.56
7.56
7.54
7.54
7.52
7.51
7.42
7.40
7.24
7.23
7.22
7.20
7.20
7.19
7.18
7.17
7.15
7.05
7.05
7.03
7.02
7.00
6.98
6.96
6.95
6.94
6.93
6.91
5.36
0.95
1.00
2.96
1.08
3.21
5.10
0.92
f1 (ppm)
O
F
N
H
N
H
4ab

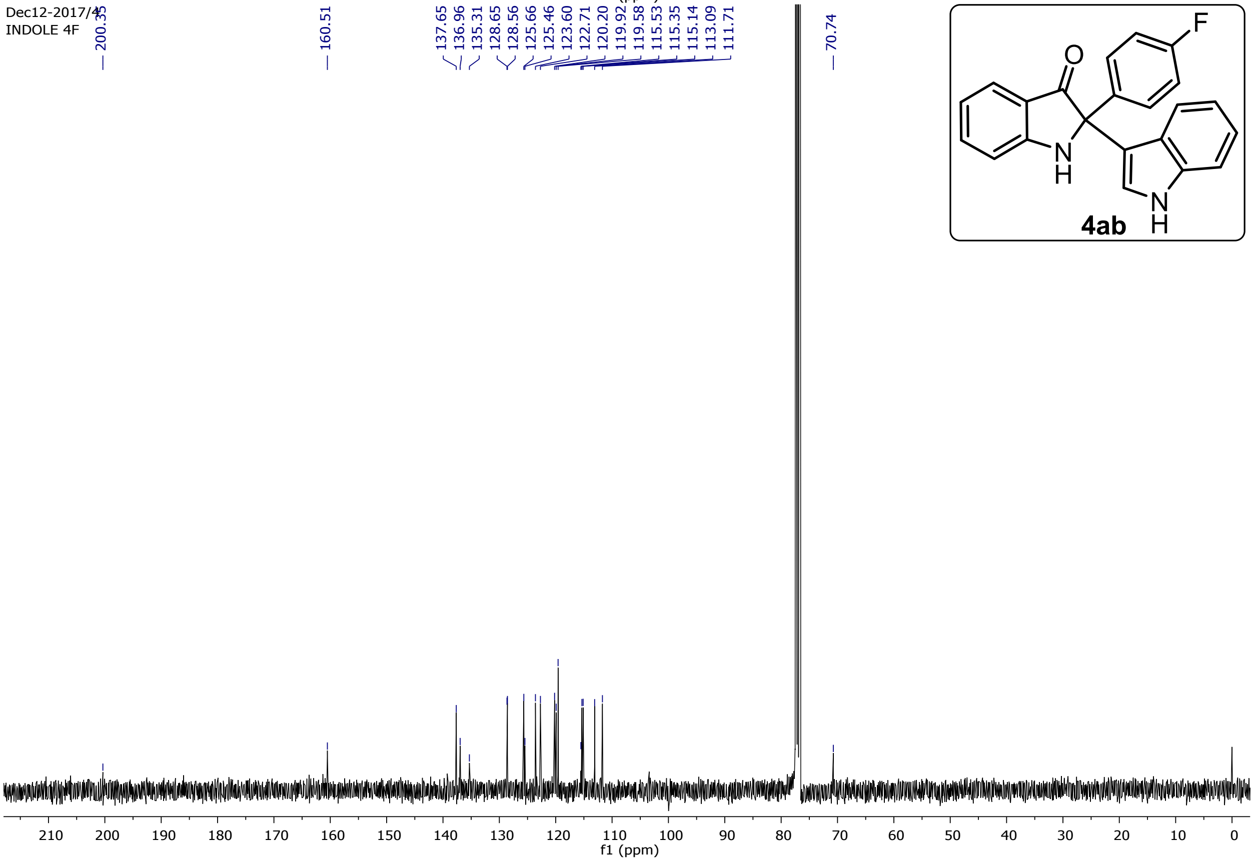
Dec12-2017/4
INDOLE 4F
200.35
160.51
137.65
136.96
135.31
128.65
128.56
125.66
125.46
123.60
122.71
120.20
119.92
119.58
115.53
115.35
115.14
113.09
111.71
70.74
f1 (ppm)
O
F
N
H
N
H
4ab

Qualitative Compound Report

**Data File**	INDOLE 4F.d	**Sample Name**	INDOLE 4F
**Sample Type**	Sample	**Position**	P2-E2
**Instrument Name**	Instrument 1	**User Name**	
**Acq Method**	water_meoh_grad_6min_reg.m	**Acquired Time**	1/5/2018 1:27:05 PM
**IRM Calibration Status**	Success	**DA Method**	PROCESSNEW.m
**Comment**			

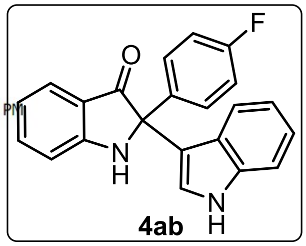

4ab

**Sample Group**		**Info.**	
**Stream Name**	LC 1	**Acquisition SW Version**	6200 series TOF/6500 series Q-TOF B.06.01 (B6172 SP1)

Compound Table

Compound Label	RT	Mass	Abund	Formula	Tgt Mass	Diff (ppm)
Cpd 1: 1.282 365.1057	1.282	342.1165	101297	C22 H15 F N2 O	342.1168	-1.06

Compound Label	*m/z*	RT	Algorithm	Mass
Cpd 1: 1.282 365.1057	365.1057	1.282	Find By Formula	342.1165

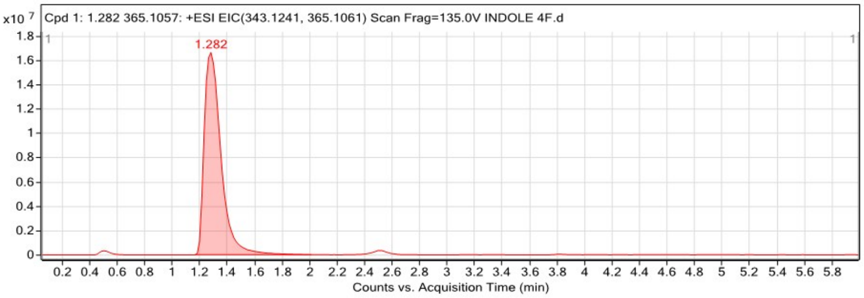

MS Zoomed Spectrum

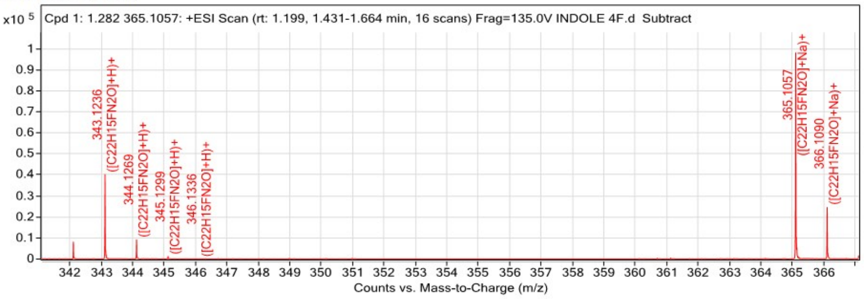

MS Spectrum Peak List

m/z	*Calc m/z*	Diff(ppm)	z	Abund	Formula	Ion
343.1236	343.1241	1.41	1	40267.52	C22H15FN2O	(M+H)+
344.1269	344.1273	1.08	1	9355.85	C22H15FN2O	(M+H)+

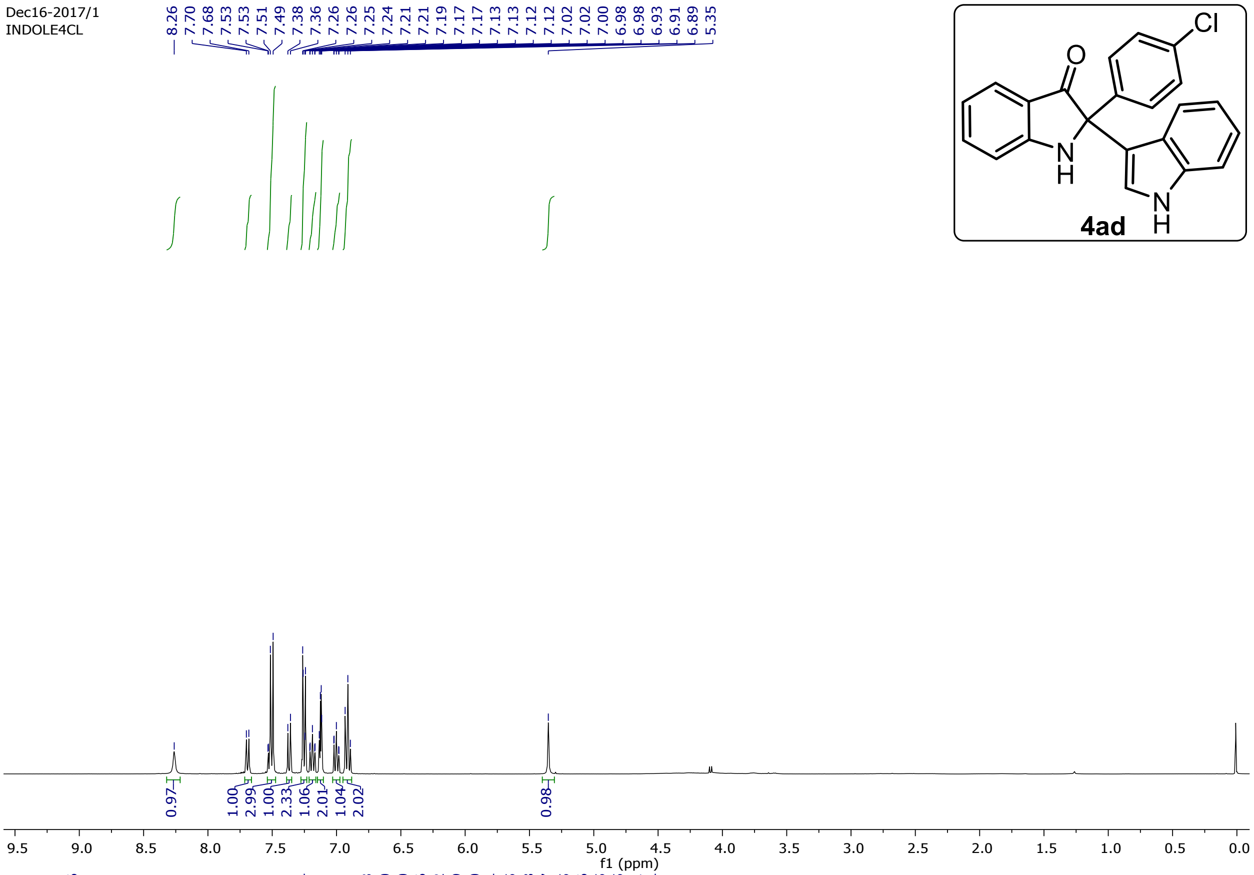
Dec16-2017/1
INDOLE4CL
8.26
7.70
7.68
7.53
7.53
7.51
7.49
7.38
7.36
7.26
7.26
7.25
7.24
7.21
7.21
7.19
7.17
7.17
7.13
7.13
7.12
7.12
7.02
7.02
7.00
6.98
6.98
6.93
6.91
6.89
5.35
Cl
O
N
H
N
H
4ad
0.97
1.00
2.99
1.00
2.33
1.06
2.01
1.04
2.02
0.98
9.5
9.0
8.5
8.0
7.5
7.0
6.5
6.0
5.5
5.0
4.5
4.0
3.5
3.0
2.5
2.0
1.5
1.0
0.5
0.0
f1 (ppm)

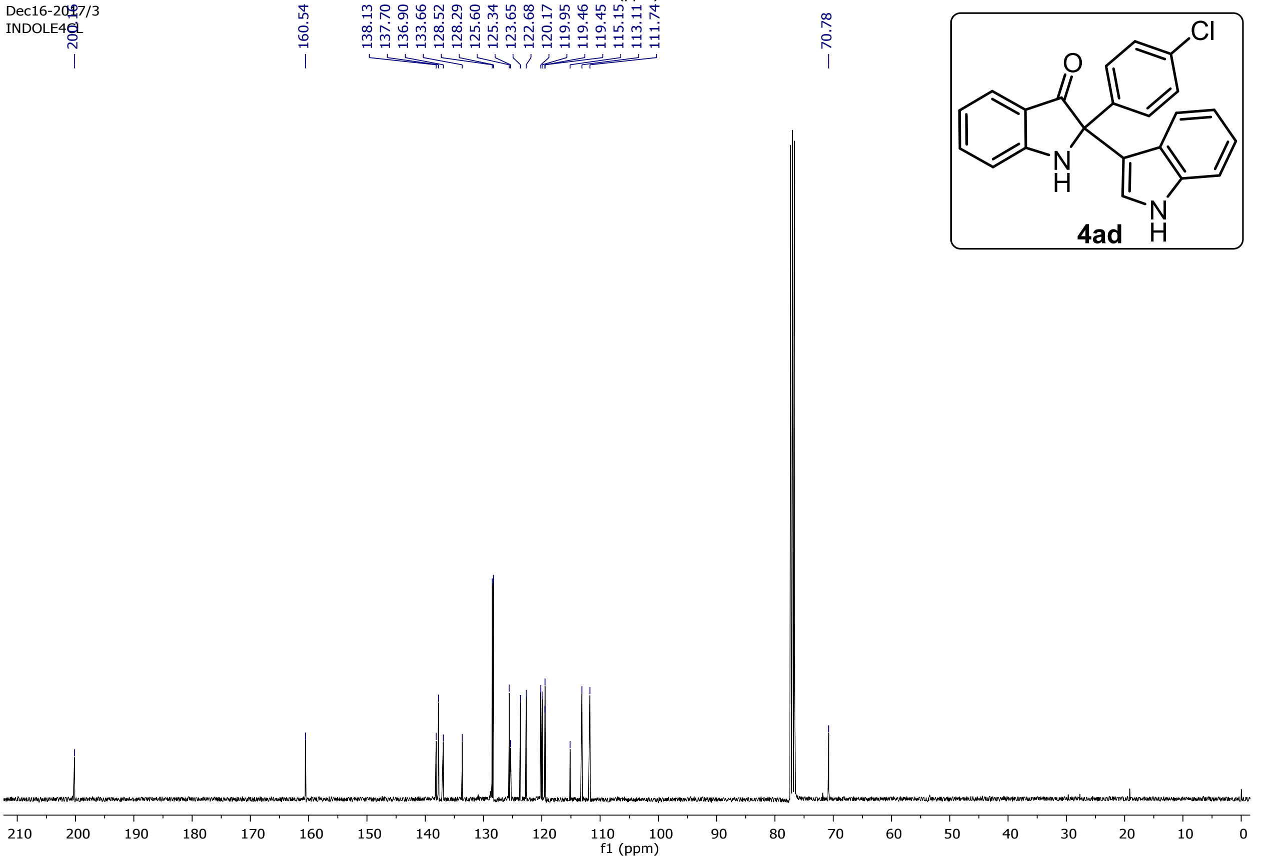
Dec16-2017/3
INDOLE4CL
160.54
138.13
137.70
136.90
133.66
128.52
128.29
125.60
125.34
123.65
122.68
120.17
119.95
119.46
119.45
115.15
113.11
111.74
70.78
Cl
O
N
H
N
H
4ad
210
200
190
180
170
160
150
140
130
120
110
100
90
80
70
60
50
40
30
20
10
0
f1 (ppm)

Qualitative Compound Report

**Data File**	Indole 4 Cl.d	**Sample Name**	Indole 4 Cl
**Sample Type**	Sample	**Position**	P1-F9
**Instrument Name**	Instrument 1	**User Name**	
**Acq Method**	water_acn_grad_6min_reg.m	**Acquired Time**	10/18/2017 10:28:10 PM
**IRM Calibration Status**	Success	**DA Method**	PROCESSNEW.m
**Comment**			

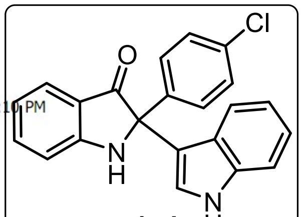

4ad

**Sample Group**		**Info.**	
**Stream Name**	LC 1	**Acquisition SW Version**	6200 series TOF/6500 series Q-TOF B.06.01 (B6172 SP1)

Compound Table

Compound Label	RT	Mass	Abund	Formula	Tgt Mass	Diff (ppm)
Cpd 1: 1.132	1.132	358.0877	65442	C22 H15 Cl N2 O	358.0873	1.2

Compound Label	*m/z*	RT	Algorithm	Mass
Cpd 1: 1.132	381.077	1.132	Find By Formula	358.0877

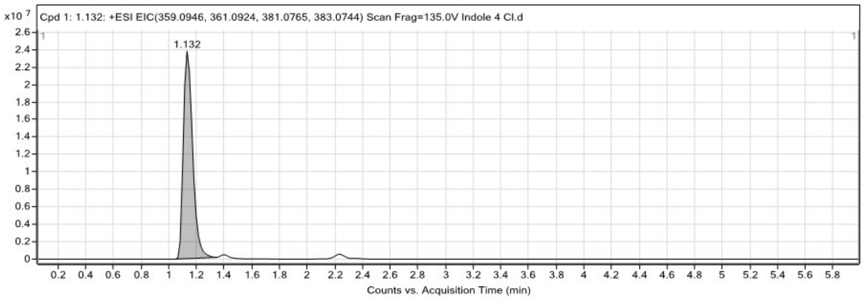

MS Zoomed Spectrum

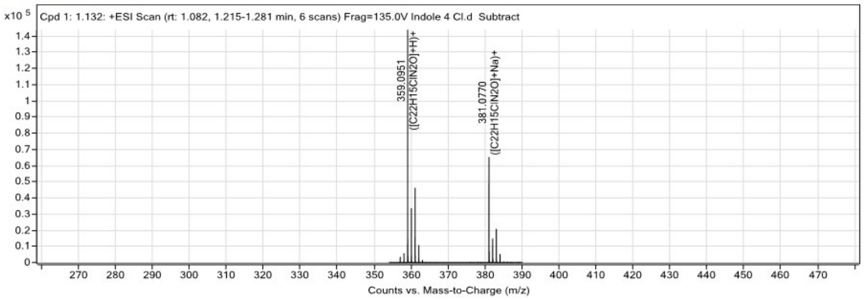

MS Spectrum Peak List

m/z	*Calc m/z*	Diff(ppm)	z	Abund	Formula	Ion
359.0951	359.0946	-1.47	1	144883.69	C22H15ClN2O	(M+H)+
360.0982	360.0978	-1.13	1	34112.25	C22H15ClN2O	(M+H)+

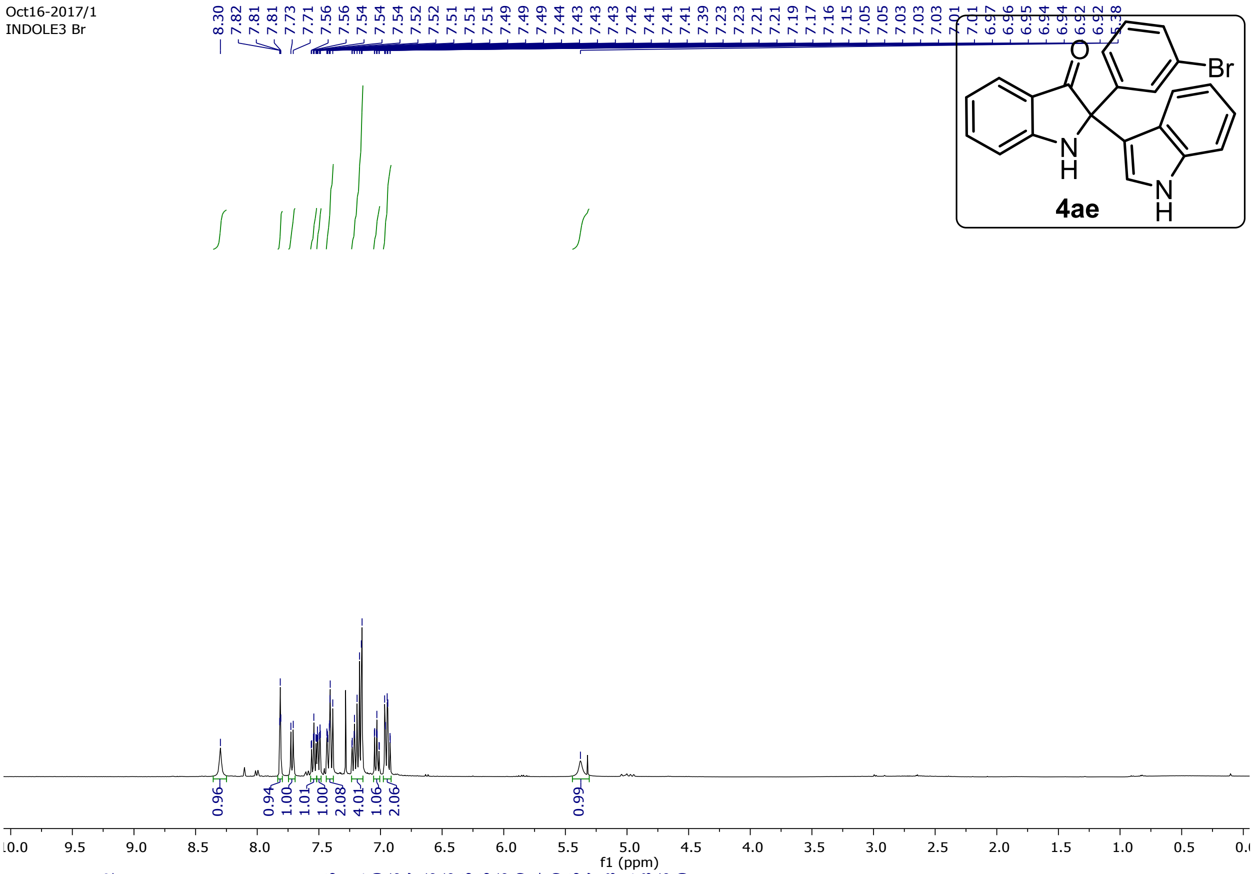
Oct16-2017/1
INDOLE3 Br
8.30 7.82 7.81 7.81 7.73 7.71 7.56 7.56 7.54 7.54 7.54 7.52 7.52 7.51 7.51 7.51 7.49 7.49 7.49 7.44 7.43 7.43 7.43 7.42 7.41 7.41 7.41 7.39 7.23 7.23 7.21 7.21 7.19 7.17 7.16 7.15 7.05 7.05 7.03 7.03 7.03 7.01 7.01 6.97 6.96 6.95 6.94 6.94 6.92 6.92 5.38
0.96 0.94 1.00 1.01 1.00 2.08 4.01 1.06 2.06 0.99
O
Br
N
H
4ae
f1 (ppm)
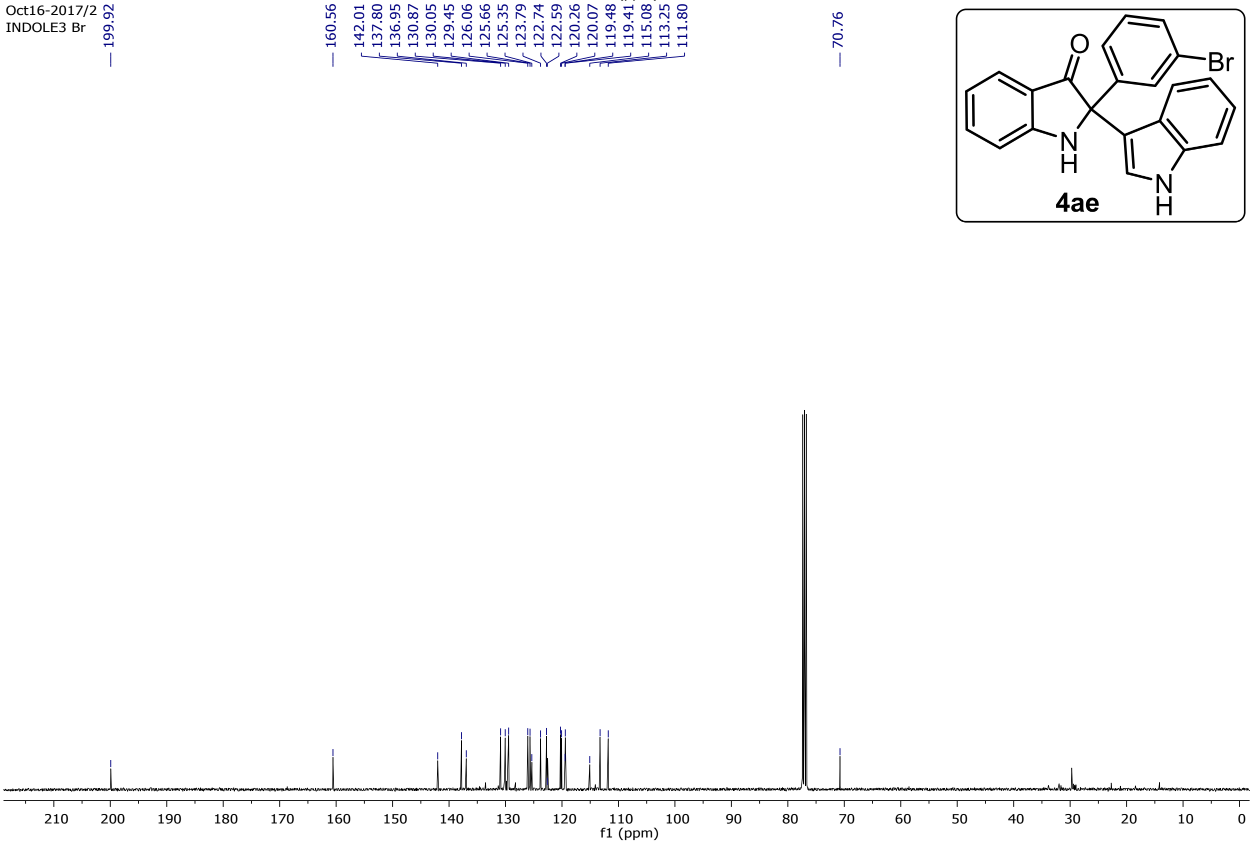
Oct16-2017/2
INDOLE3 Br
199.92 160.56 142.01 137.80 136.95 130.87 130.05 129.45 126.06 125.66 125.35 123.79 122.74 122.59 120.26 120.07 119.48 119.41 115.08 113.25 111.80 70.76
O
Br
N
H
4ae
f1 (ppm)

Qualitative Compound Report

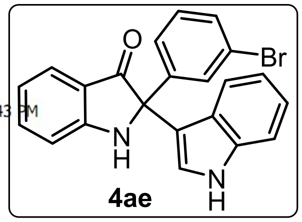

**Data File**	Inodle 3 Br.d	**Sample Name**	Indole 3 Br
**Sample Type**	Sample	**Position**	P1-F7
**Instrument Name**	Instrument 1	**User Name**	
**Acq Method**	water_acn_grad_6min_reg.m	**Acquired Time**	10/18/2017 9:59:43 PM
**IRM Calibration Status**	Success	**DA Method**	PROCESSNEW.m
**Comment**			

**Sample Group**		**Info.**	
**Stream Name**	LC 1	**Acquisition SW Version**	6200 series TOF/6500 series Q-TOF B.06.01 (B6172 SP1)

Compound Table

Compound Label	RT	Mass	Abund	Formula	Tgt Mass	Diff (ppm)
Cpd 1: 1.096	1.096	402.0368	172822	C22 H15 Br N2 O	402.0368	0.14

Compound Label	*m/z*	RT	Algorithm	Mass
Cpd 1: 1.096	403.0441	1.096	Find By Formula	402.0368

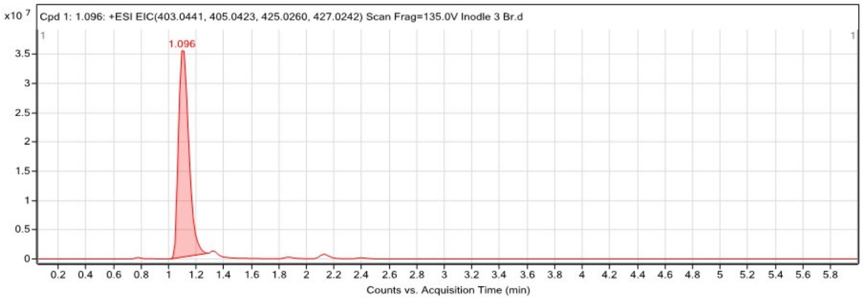

MS Zoomed Spectrum

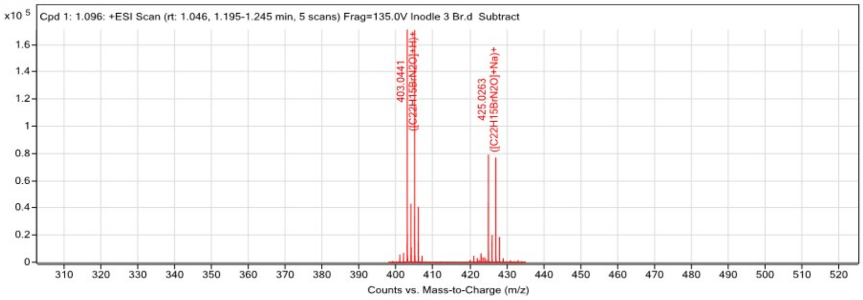

MS Spectrum Peak List

m/z	*Calc m/z*	Diff(ppm)	z	Abund	Formula	Ion
403.0441	403.0441	-0.18	1	172821.62	C22H15BrN2O	(M+H)+
404.0469	404.0472	0.97	1	43398.09	C22H15BrN2O	(M+H)+

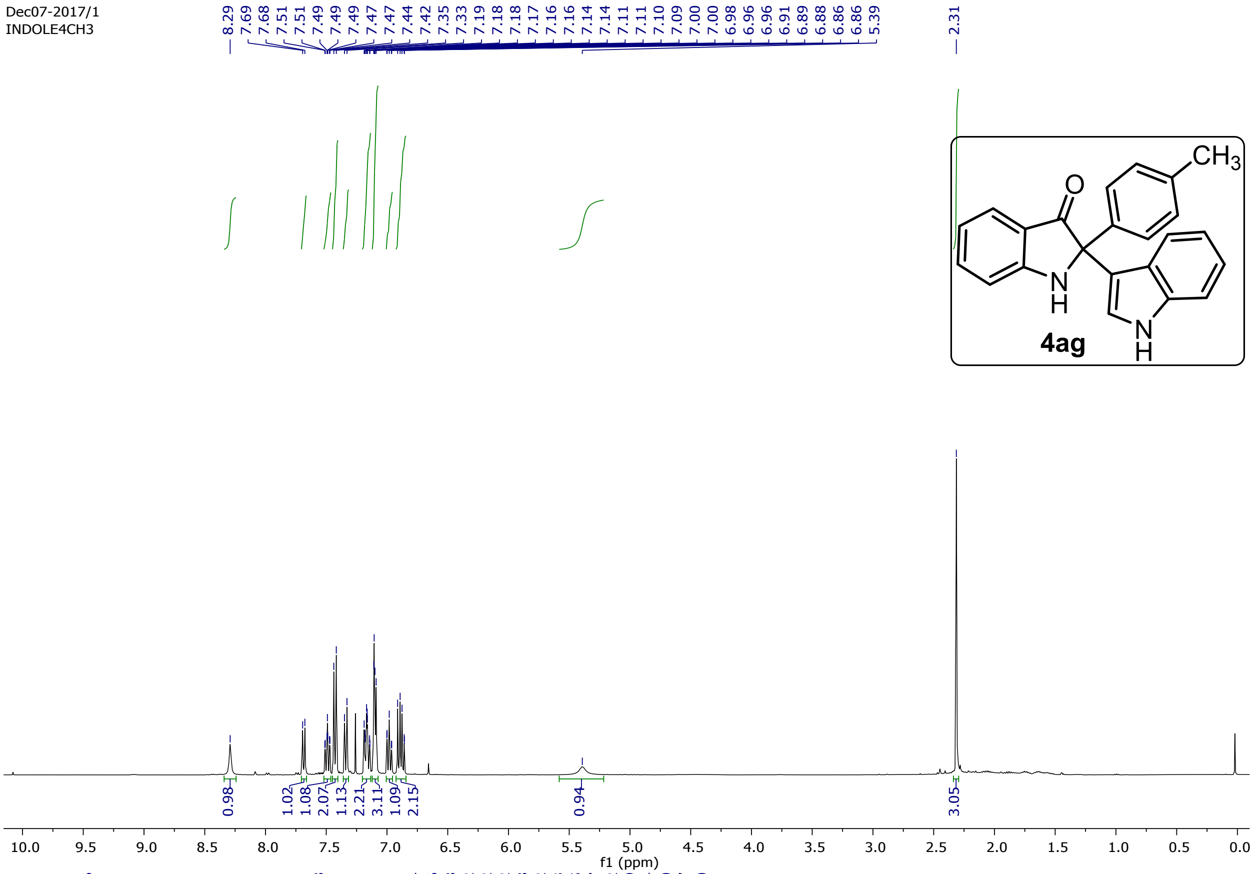
Dec07-2017/1
INDOLE4CH3
8.29 7.69 7.68 7.51 7.49 7.49 7.49 7.47 7.47 7.44 7.42 7.35 7.33 7.19 7.18 7.18 7.17 7.16 7.16 7.14 7.14 7.11 7.11 7.10 7.09 7.00 7.00 6.98 6.96 6.96 6.91 6.89 6.88 6.86 6.86 5.39
2.31
CH3
O
N
H
4ag
N
H
0.98
1.02
1.08
2.07
1.13
2.21
3.11
1.09
2.15
0.94
3.05
10.0 9.5 9.0 8.5 8.0 7.5 7.0 6.5 6.0 5.5 5.0 4.5 4.0 3.5 3.0 2.5 2.0 1.5 1.0 0.5 0.0
f1 (ppm)

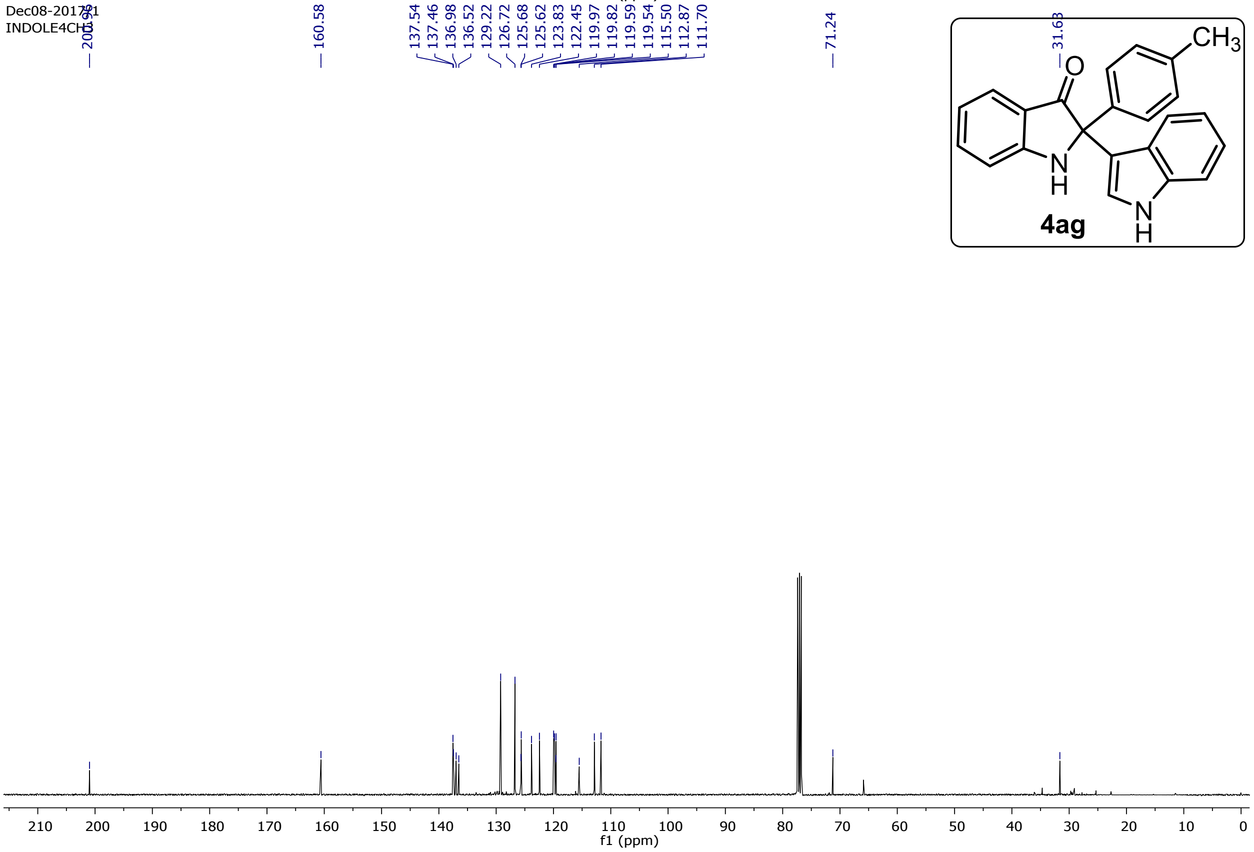
INDOLE4CH3
160.58
137.54 137.46 136.98 136.52 129.22 126.72 125.68 125.62 123.83 122.45 119.97 119.82 119.59 119.54 115.50 112.87 111.70
71.24
31.63
CH3
O
N
H
4ag
N
H
210 200 190 180 170 160 150 140 130 120 110 100 90 80 70 60 50 40 30 20 10 0
f1 (ppm)

Qualitative Compound Report

**Data File**	Indole 4 Me.d	**Sample Name**	Indole 4 Me
**Sample Type**	Sample	**Position**	P1-F8
**Instrument Name**	Instrument 1	**User Name**	
**Acq Method**	water_acn_grad_6min_reg.m	**Acquired Time**	10/18/2017 10:13:57 PM
**IRM Calibration Status**	Success	**DA Method**	PROCESSNEW.m
**Comment**			

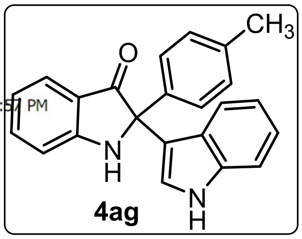

4ag

**Sample Group**		**Info.**	
**Stream Name**	LC 1	**Acquisition SW Version**	6200 series TOF/6500 series Q-TOF B.06.01 (B6172 SP1)

Compound Table

Compound Label	RT	Mass	Abund	Formula	Tgt Mass	Diff (ppm)
Cpd 1: 1.008	1.008	338.1419	133416	C23 H18 N2 O	338.1419	-0.11

Compound Label	*m/z*	RT	Algorithm	Mass
Cpd 1: 1.008	339.1492	1.008	Find By Formula	338.1419

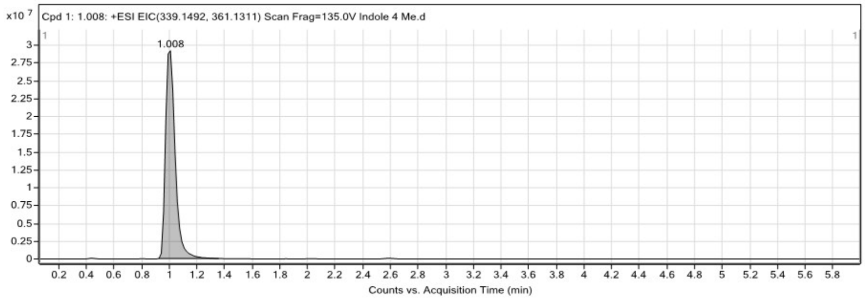

MS Zoomed Spectrum

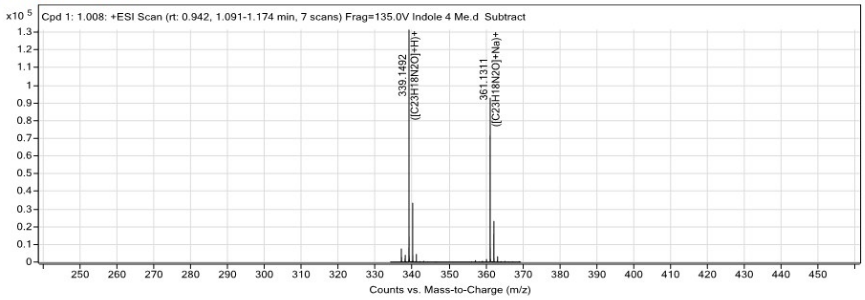

MS Spectrum Peak List

m/z	*Calc m/z*	Diff(ppm)	z	Abund	Formula	Ion
339.1492	339.1492	0.11	1	133415.77	C23H18N2O	(M+H)+
340.1524	340.1524	0.12	1	34113.54	C23H18N2O	(M+H)+

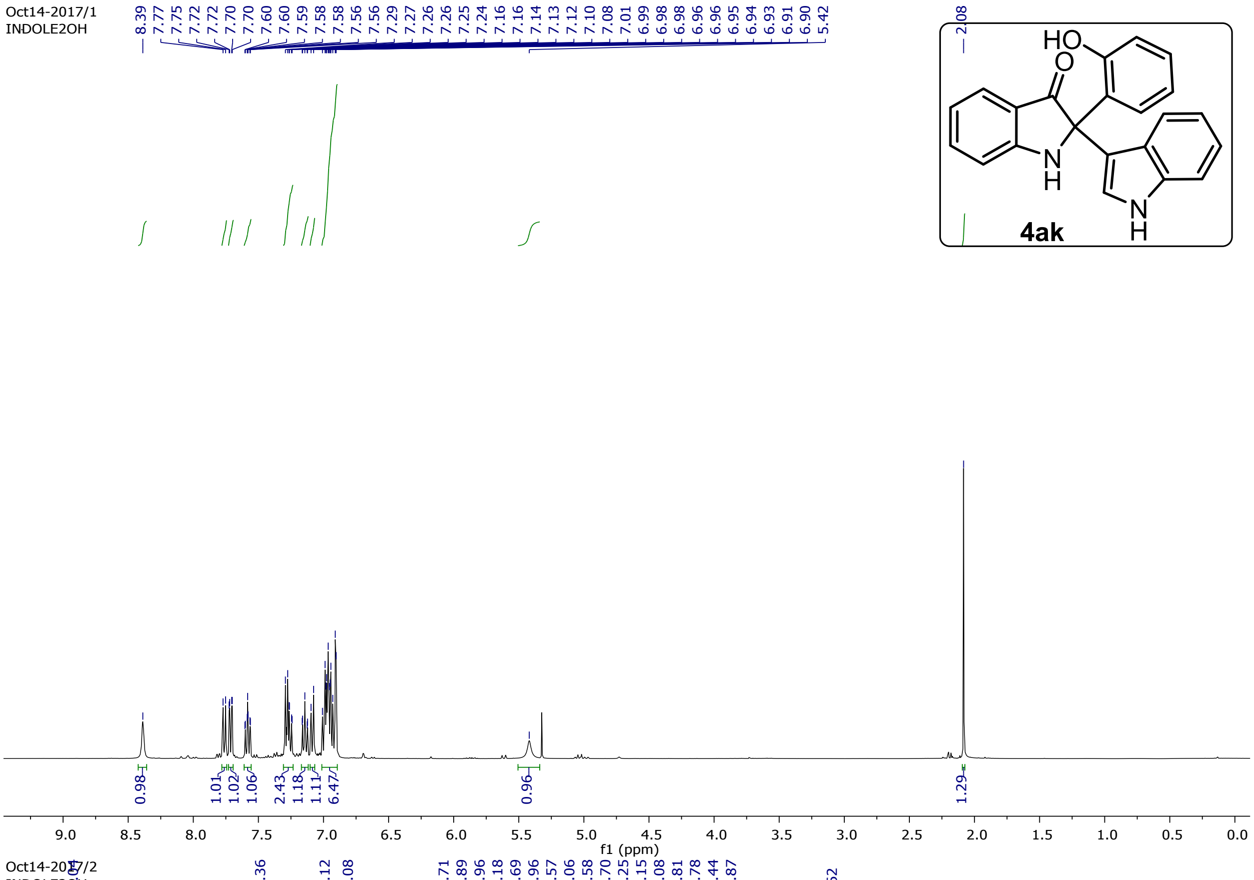
Oct14-2017/1
INDOLE2OH
8.39 7.77 7.75 7.72 7.72 7.70 7.70 7.60 7.60 7.59 7.58 7.58 7.56 7.56 7.29 7.27 7.26 7.26 7.25 7.24 7.16 7.16 7.14 7.13 7.12 7.10 7.08 7.01 6.99 6.98 6.98 6.96 6.96 6.95 6.94 6.93 6.91 6.90 5.42
2.08
HO
O
N
H
N
H
4ak
0.98 1.01 1.02 1.06 2.43 1.18 1.11 6.47 0.96 1.29
9.0 8.5 8.0 7.5 7.0 6.5 6.0 5.5 5.0 4.5 4.0 3.5 3.0 2.5 2.0 1.5 1.0 0.5 0.0
f1 (ppm)

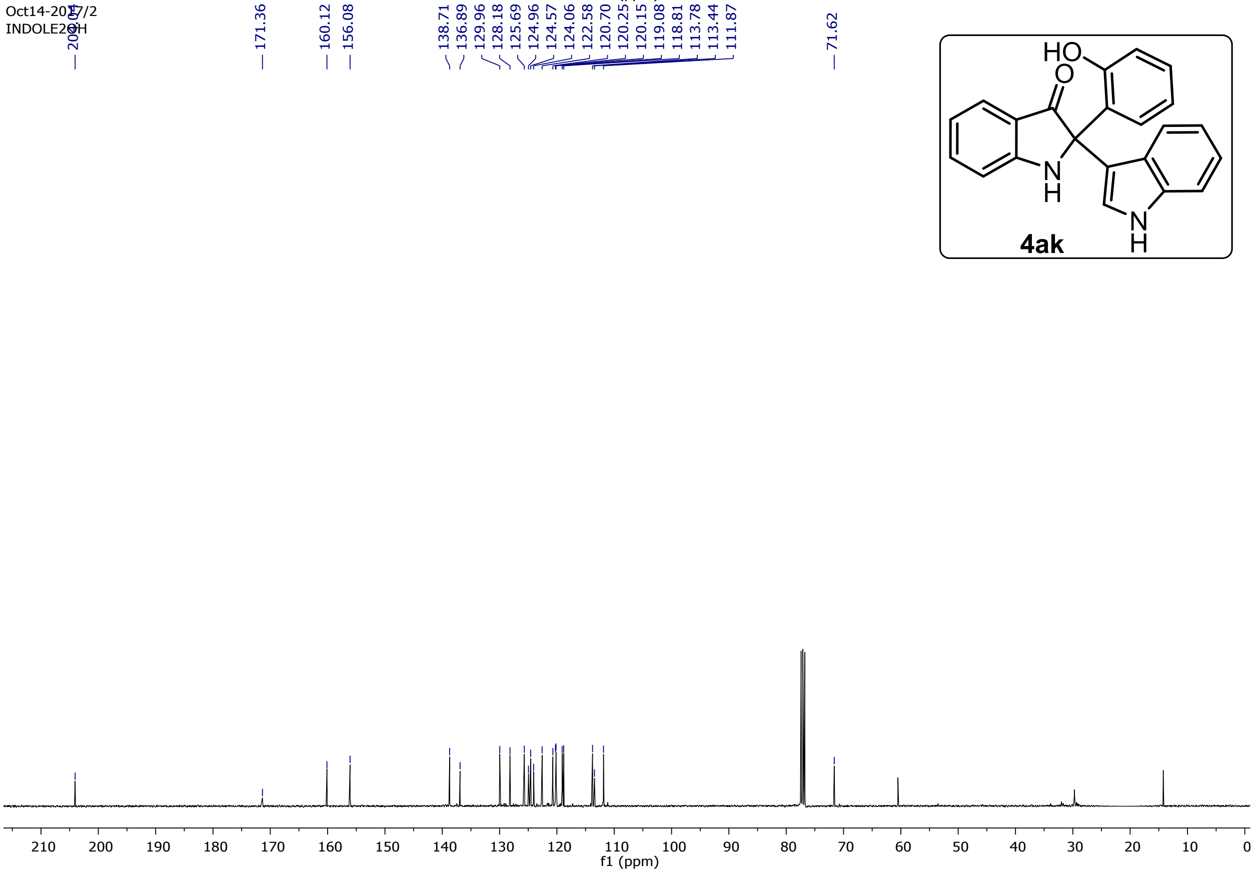
Oct14-2017/2
INDOLE2OH
204.07
171.36
160.12 156.08
138.71 136.89 129.96 128.18 125.69 124.96 124.57 124.06 122.58 120.70 120.25 120.15 119.08 118.81 113.78 113.44 111.87
71.62
HO
O
N
H
N
H
4ak
210 200 190 180 170 160 150 140 130 120 110 100 90 80 70 60 50 40 30 20 10 0
f1 (ppm)

Qualitative Compound Report

**Data File**	indole 2 OH.d	**Sample Name**	Indole 2 OH
**Sample Type**	Sample	**Position**	P1-F6
**Instrument Name**	Instrument 1	**User Name**	
**Acq Method**	water_acn_grad_6min_reg.m	**Acquired Time**	10/18/2017 9:45:32 PM
**IRM Calibration Status**	Success	**DA Method**	PROCESSNEW.m
**Comment**			

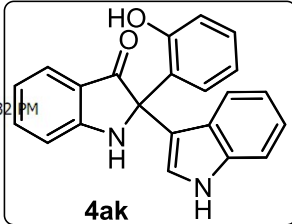

4ak

**Sample Group**		**Info.**	
**Stream Name**	LC 1	**Acquisition SW Version**	6200 series TOF/6500 series Q-TOF B.06.01 (B6172 SP1)

Compound Table

Compound Label	RT	Mass	Abund	Formula	Tgt Mass	Diff (ppm)
Cpd 1: 0.722	0.722	340.1214	88191	C22 H16 N2 O2	340.1212	0.51

Compound Label	*m/z*	RT	Algorithm	Mass
Cpd 1: 0.722	341.1286	0.722	Find By Formula	340.1214

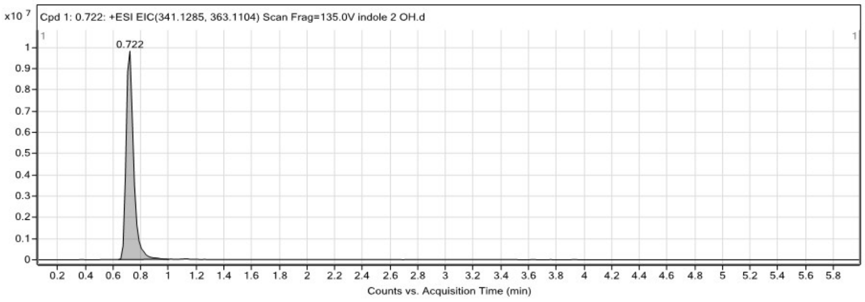

MS Zoomed Spectrum

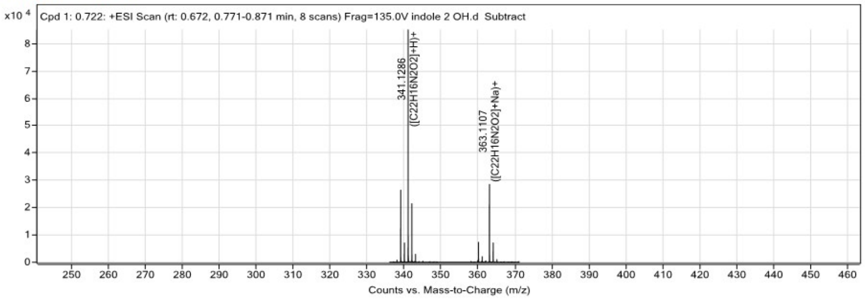

MS Spectrum Peak List

m/z	*Calc m/z*	Diff(ppm)	z	Abund	Formula	Ion
341.1286	341.1285	-0.31	1	88191	C22H16N2O2	(M+H)+
342.1318	342.1316	-0.51	1	21964.47	C22H16N2O2	(M+H)+

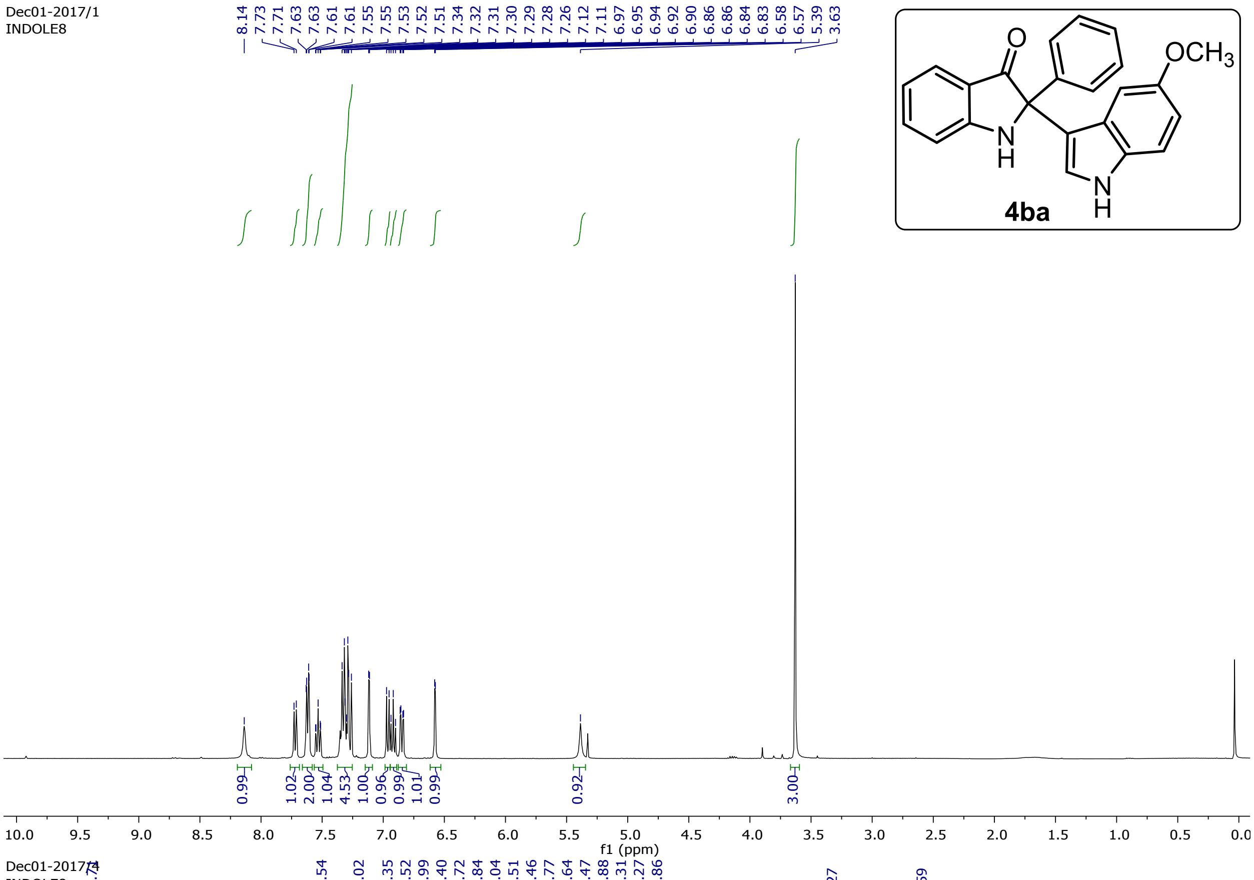
Dec01-2017/1
INDOLE8
8.14
7.73
7.71
7.63
7.63
7.61
7.61
7.55
7.55
7.53
7.52
7.51
7.34
7.32
7.31
7.30
7.29
7.28
7.26
7.12
7.11
6.97
6.95
6.94
6.92
6.90
6.86
6.86
6.84
6.83
6.58
6.57
5.39
3.63
0.99
1.02
2.00
1.04
4.53
1.00
0.96
0.99
1.01
0.99
0.92
3.00
f1 (ppm)
OCH3
4ba

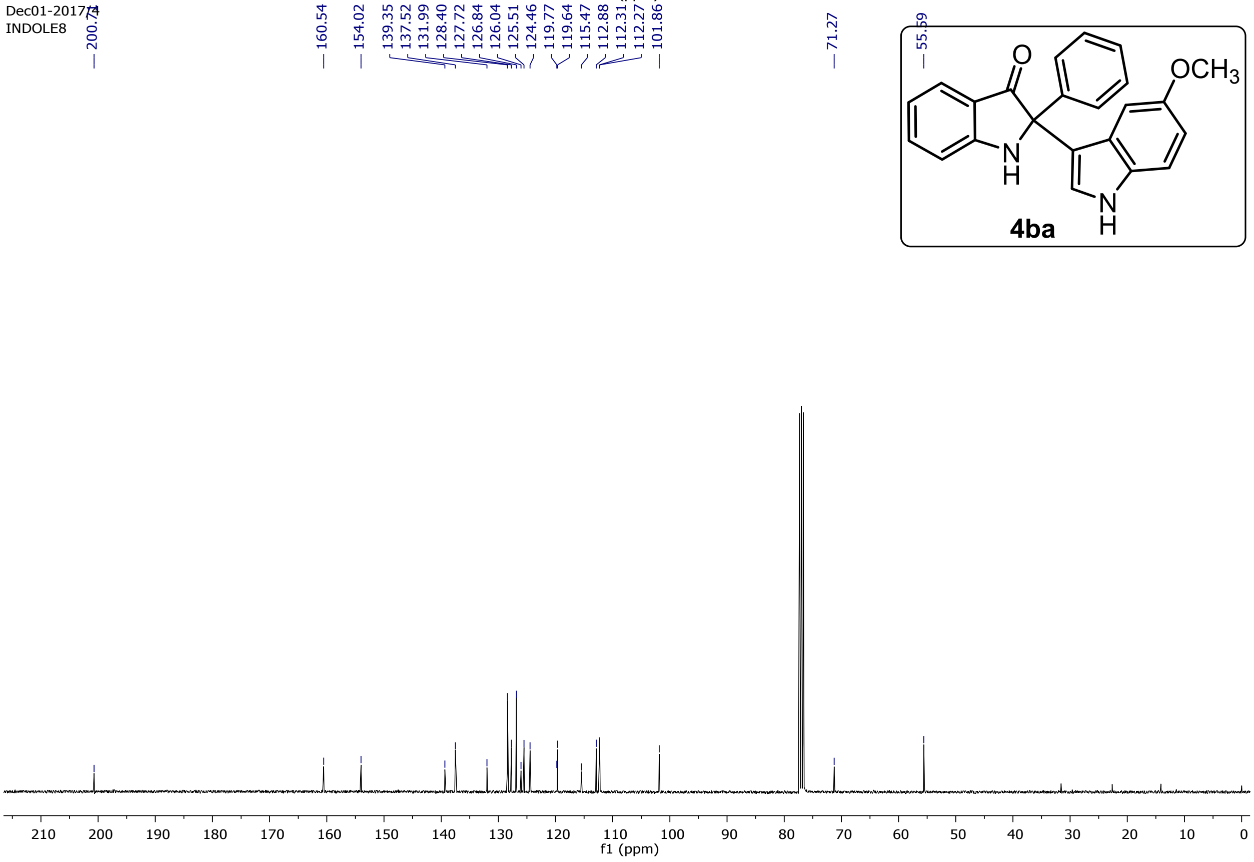
Dec01-2017/4
INDOLE8
200.74
160.54
154.02
139.35
137.52
131.99
128.40
127.72
126.84
126.04
125.51
124.46
119.77
119.64
115.47
112.88
112.31
112.27
101.86
71.27
55.59
f1 (ppm)
OCH3
4ba

Qualitative Compound Report

**Data File**	INDOLE 8.d	**Sample Name**	INDOLE 8
**Sample Type**	Sample	**Position**	P2-E4
**Instrument Name**	Instrument 1	**User Name**	
**Acq Method**	water_meoh_grad_6min_reg.m	**Acquired Time**	1/5/2018 1:55:20 PM
**IRM Calibration Status**	Success	**DA Method**	PROCESSNEW.m
**Comment**			

**Sample Group**		**Info.**	
**Stream Name**	LC 1	**Acquisition SW Version**	6200 series TOF/6500 series Q-TOF B.06.01 (B6172 SP1)

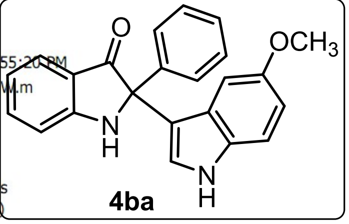

4ba

Compound Table

Compound Label	RT	Mass	Abund	Formula	Tgt Mass	Diff (ppm)
Cpd 1: 1.042 409.1613	1.042	354.136	103571	C23 H18 N2 O2	354.1368	-2.22

Compound Label	*m/z*	RT	Algorithm	Mass
Cpd 1: 1.042 409.1613	377.1253	1.042	Find By Formula	354.136

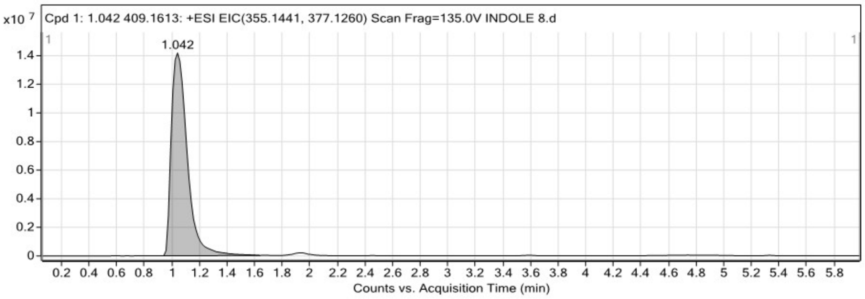

MS Zoomed Spectrum

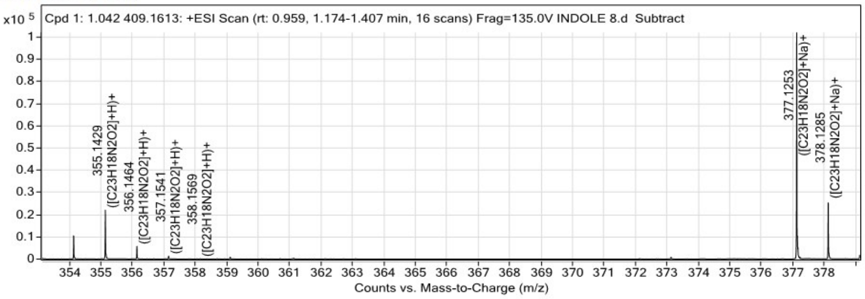

MS Spectrum Peak List

m/z	*Calc m/z*	Diff(ppm)	z	Abund	Formula	Ion
355.1429	355.1441	3.3	1	22090.02	C23H18N2O2	(M+H)+
356.1464	356.1473	2.54	1	5752	C23H18N2O2	(M+H)+

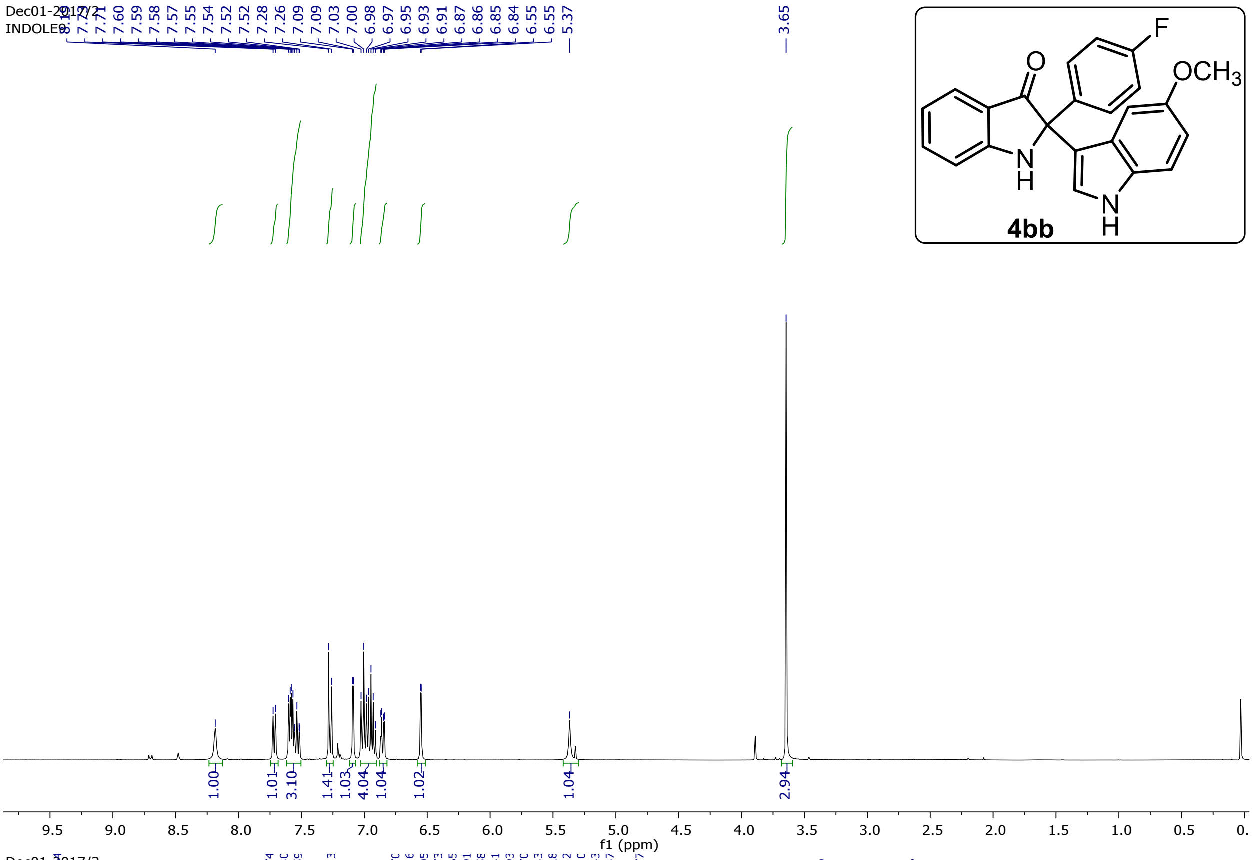
Dec01-2017/2
INDOLE9
7.72 7.71 7.60 7.59 7.58 7.57 7.55 7.54 7.52 7.52 7.28 7.26 7.09 7.09 7.03 7.00 6.98 6.97 6.95 6.93 6.91 6.87 6.86 6.85 6.84 6.55 6.55 5.37
3.65
1.00 1.01 3.10 1.41 1.03 4.04 1.04 1.02 1.04 2.94
9.5 9.0 8.5 8.0 7.5 7.0 6.5 6.0 5.5 5.0 4.5 4.0 3.5 3.0 2.5 2.0 1.5 1.0 0.5 0.
f1 (ppm)
F
OCH3
O
N
H
N
H
4bb

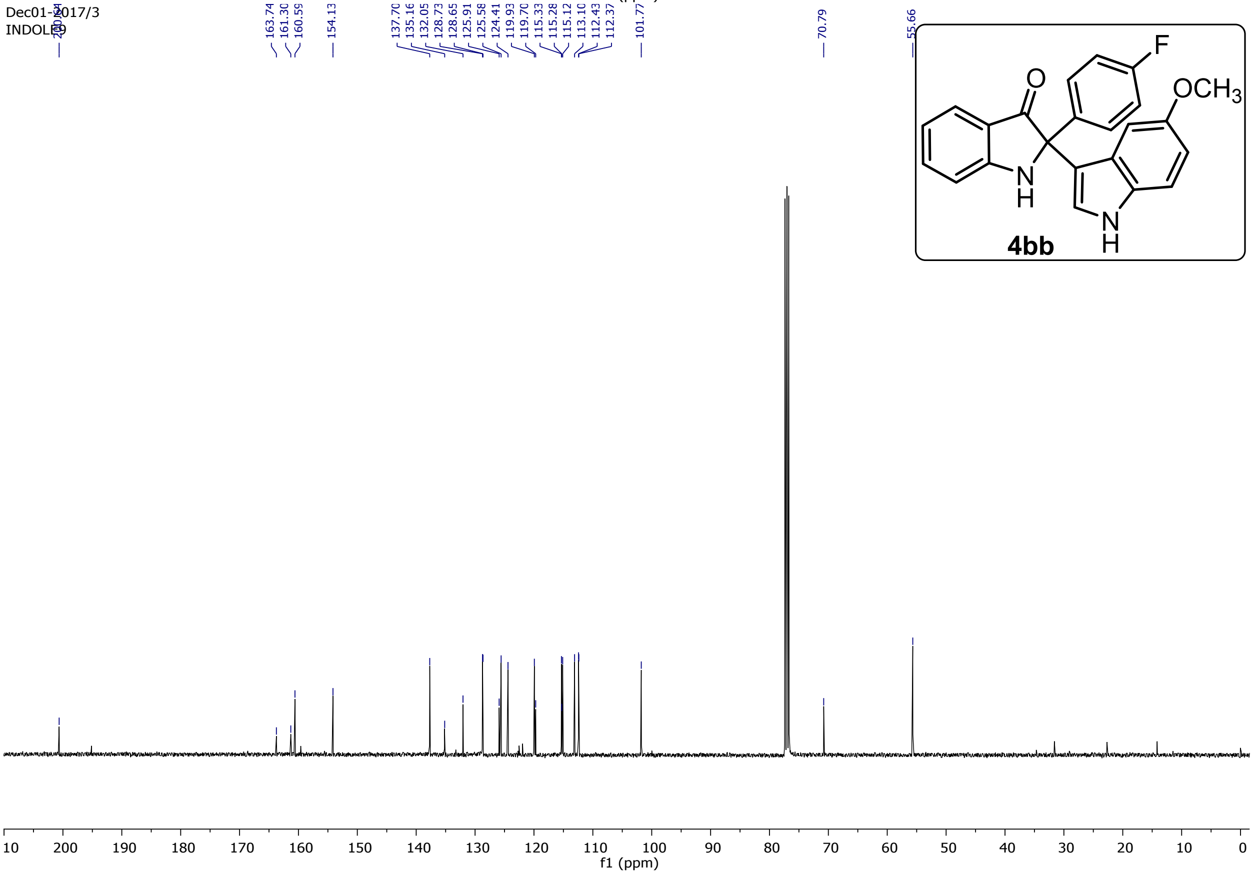
Dec01-2017/3
INDOLE9
163.74 161.30 160.59
154.13
137.70 135.16 132.05 128.73 128.65 125.91 125.58 124.41 119.93 119.70 115.33 115.28 115.12 113.10 112.43 112.37
101.77
70.79
55.66
10 200 190 180 170 160 150 140 130 120 110 100 90 80 70 60 50 40 30 20 10 0
f1 (ppm)
F
OCH3
O
N
H
N
H
4bb

Qualitative Compound Report

**Data File**	INDOLE 9.d	**Sample Name**	INDOLE 9
**Sample Type**	Sample	**Position**	P2-E5
**Instrument Name**	Instrument 1	**User Name**	
**Acq Method**	water_meoh_grad_6min_reg.m	**Acquired Time**	1/5/2018 2:09:27 PM
**IRM Calibration Status**	Success	**DA Method**	PROCESSNEW.m
**Comment**			

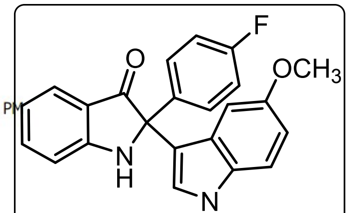

4bb

**Sample Group**		**Info.**	
**Stream Name**	LC 1	**Acquisition SW Version**	6200 series TOF/6500 series Q-TOF B.06.01 (B6172 SP1)

Compound Table

Compound Label	RT	Mass	Abund	Formula	Tgt Mass	Diff (ppm)
Cpd 1: 1.176 395.1157	1.176	372.1264	96028	C23 H17 F N2 O2	372.1274	-2.63

Compound Label	*m/z*	RT	Algorithm	Mass
Cpd 1: 1.176 395.1157	395.1157	1.176	Find By Formula	372.1264

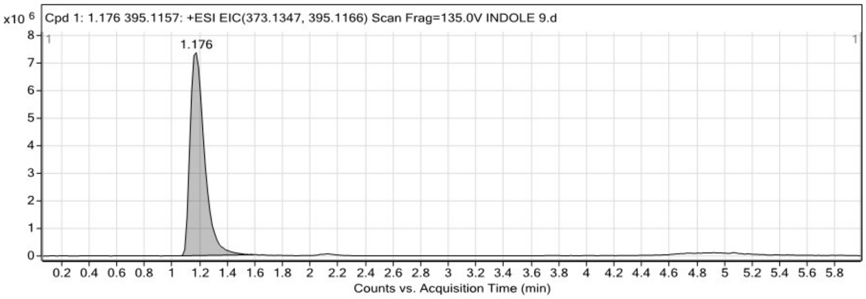

MS Zoomed Spectrum

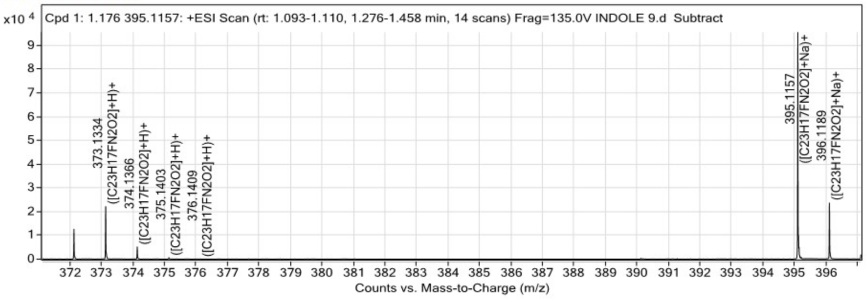

MS Spectrum Peak List

m/z	*Calc m/z*	Diff(ppm)	z	Abund	Formula	Ion
373.1334	373.1347	3.51	1	22215.47	C23H17FN2O2	(M+H)+
374.1366	374.1379	3.53	1	5205.3	C23H17FN2O2	(M+H)+

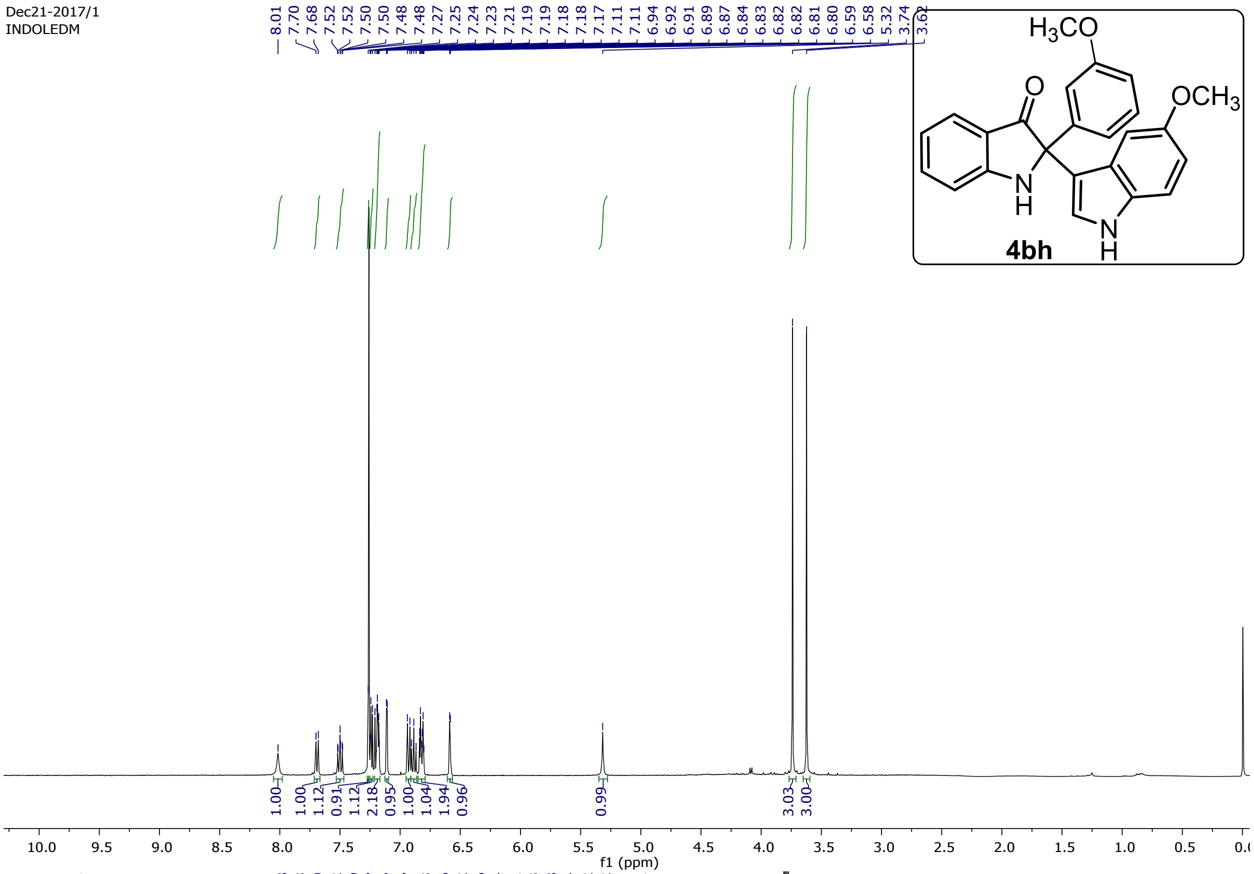
Dec21-2017/1
INDOLEDM
8.01 7.70 7.68 7.52 7.52 7.50 7.50 7.48 7.48 7.27 7.25 7.24 7.23 7.21 7.19 7.19 7.18 7.18 7.17 7.11 7.11 6.94 6.92 6.91 6.89 6.87 6.84 6.83 6.82 6.82 6.81 6.80 6.59 6.58 5.32 3.74 3.62
H3CO
O
OCH3
N
H
N
H
4bh
1.00 1.00 1.12 0.91 1.12 2.18 0.95 1.00 1.04 1.94 0.96 0.99 3.03 3.00
10.0 9.5 9.0 8.5 8.0 7.5 7.0 6.5 6.0 5.5 5.0 4.5 4.0 3.5 3.0 2.5 2.0 1.5 1.0 0.5 0.0
f1 (ppm)

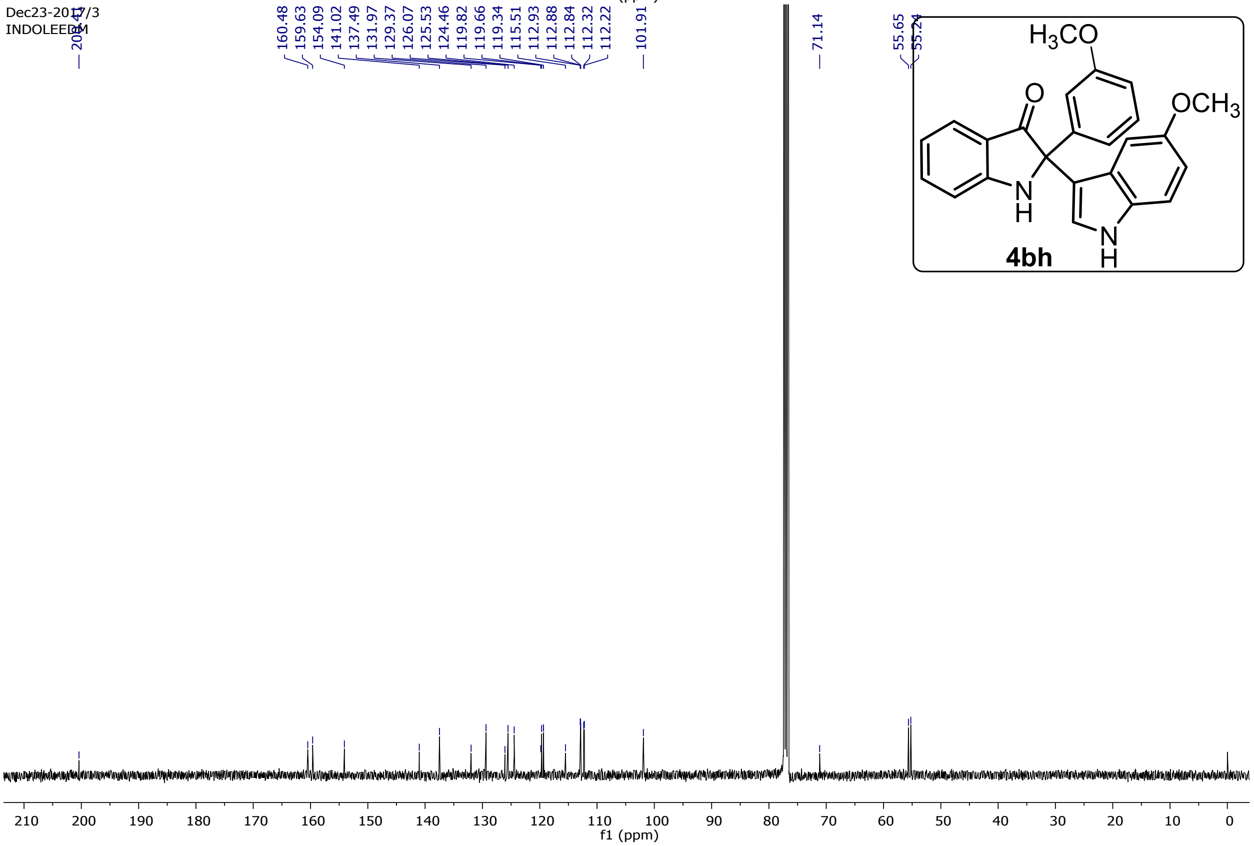
Dec23-2017/3
INDOLEEDM
160.48 159.63 154.09 141.02 137.49 131.97 129.37 126.07 125.53 124.46 119.82 119.66 119.34 115.51 112.93 112.88 112.84 112.32 112.22 101.91 71.14 55.65 55.24
H3CO
O
OCH3
N
H
N
H
4bh
210 200 190 180 170 160 150 140 130 120 110 100 90 80 70 60 50 40 30 20 10 0
f1 (ppm)

Qualitative Compound Report

**Data File**	Blank 15.d	**Sample Name**	INDOLE DM
**Sample Type**	Sample	**Position**	P2-E6
**Instrument Name**	Instrument 1	**User Name**	
**Acq Method**	water_meoh_grad_6min_reg.m	**Acquired Time**	1/5/2018 2:23:34 PM
**IRM Calibration Status**	Success	**DA Method**	PROCESSNEW.m
**Comment**			

**Sample Group**		**Info.**	
**Stream Name**	LC 1	**Acquisition SW Version**	6200 series TOF/6500 series Q-TOF B.06.01 (B6172 SP1)

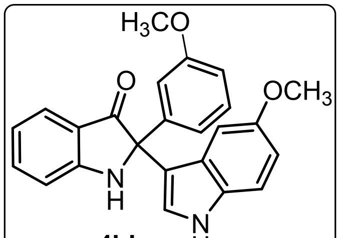

4bh

Compound Table

Compound Label	RT	Mass	Abund	Formula	Tgt Mass	Diff (ppm)
Cpd 1: 1.010 409.1613	1.01	384.1465	96761	C24 H20 N2 O3	384.1474	-2.29

Compound Label	*m/z*	RT	Algorithm	Mass
Cpd 1: 1.010 409.1613	407.1358	1.01	Find By Formula	384.1465

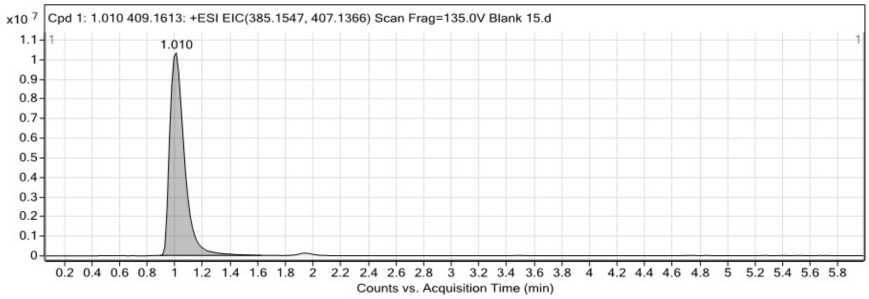

MS Zoomed Spectrum

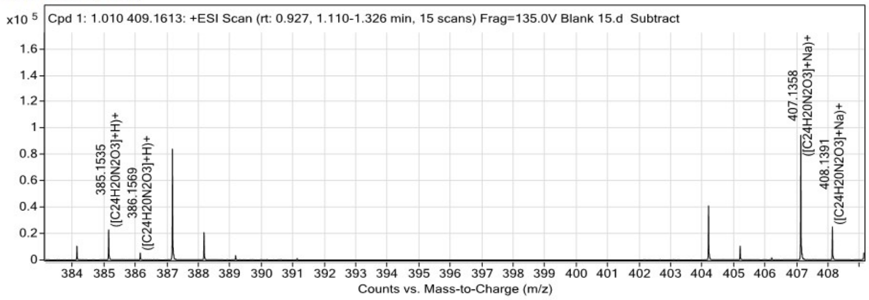

MS Spectrum Peak List

m/z	*Calc m/z*	Diff(ppm)	z	Abund	Formula	Ion
385.1535	385.1547	2.98	1	23216.58	C24H20N2O3	(M+H)+
386.1569	386.1579	2.42	1	5579	C24H20N2O3	(M+H)+

Crystal structure data for (2g):

Crystal structure of 2-(*p*-tolyl)-2-(2-(p-tolyl)-1*H*-indol-3-yl)indolin-3-one(2g):

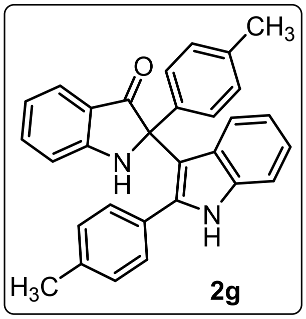

2g

(CCDC No.1832862)

Single crystals of2-(*p*-tolyl)-2-(2-(p-tolyl)-1*H*-indol-3-yl)indolin-3-one$C_{61}H_{49}Cl_3N_4O_2$was selected on aXtaLAB Pro: Kappa dual offset/far diffractometer. The crystal was kept at 93(2) K during data collection. Using Olex2,[1] the structure was solved with the ShelXT[2] structure solution program using Intrinsic Phasing and refined with theShelXL[3] refinement package using Least Squares minimization.

Crystal Data for $C_{61}H_{49}Cl_3N_4O_2$ (M =976.39 g/mol): triclinic, space group P-1 (no. 2), a = 13.13930(10) Å, b = 14.65600(10) Å, c = 15.47680(10) Å, α = 110.2300(10)°, β = 91.2620(10)°, γ = 108.1490(10)°, V = 2629.10(4) Å^3, Z = 2, T = 93(2) K, μ(CuKα) = 1.943 mm^{-1}, $Dcalc$ = 1.233 g/cm^3, 28497 reflections measured (7.156° ≤ 2Θ ≤ 149.04°), 10338 unique (R_{int} = 0.0246, R_{sigma} = 0.0226) which were used in all calculations. The final R_1 was 0.0623 (I >2σ(I)) and wR_2 was 0.1867 (all data).

**TableS1:Crystal structure data of (2g): (CCDC No.1832862)**<br>**Table 1 Crystal data and structure refinement for exp_255-IND8a.**	
Identification code	**CCDC No.1832862**
Empirical formula	$C_{61}H_{49}Cl_3N_4O_2$
Formula weight	976.39
Temperature/K	93(2)
Crystal system	triclinic

Space group	P-1
a/Å	13.13930(10)
b/Å	14.65600(10)
c/Å	15.47680(10)
α/°	110.2300(10)
β/°	91.2620(10)
γ/°	108.1490(10)
Volume/Å^3	2629.10(4)
Z	2
ρ_{calc}g/cm^3	1.233
μ/mm^{-1}	1.943
F(000)	1020.0
Crystal size/mm^3	0.2 × 0.2 × 0.1
Radiation	CuKα (λ = 1.54184)
2Θ range for data collection/°	7.156 to 149.04
Index ranges	-15 ≤ h ≤ 16, -18 ≤ k ≤ 18, -19 ≤ l ≤ 13
Reflections collected	28497
Independent reflections	10338 [R_{int} = 0.0246, R_{sigma} = 0.0226]
Data/restraints/parameters	10338/0/635
Goodness-of-fit on F^2	1.069
Final R indexes [I>=2σ (I)]	R_1 = 0.0623, wR_2 = 0.1847

Final R indexes [all data]	R_1 = 0.0645, wR_2 = 0.1867
Largest diff. peak/hole / e Å^{-3}	0.49/-0.84

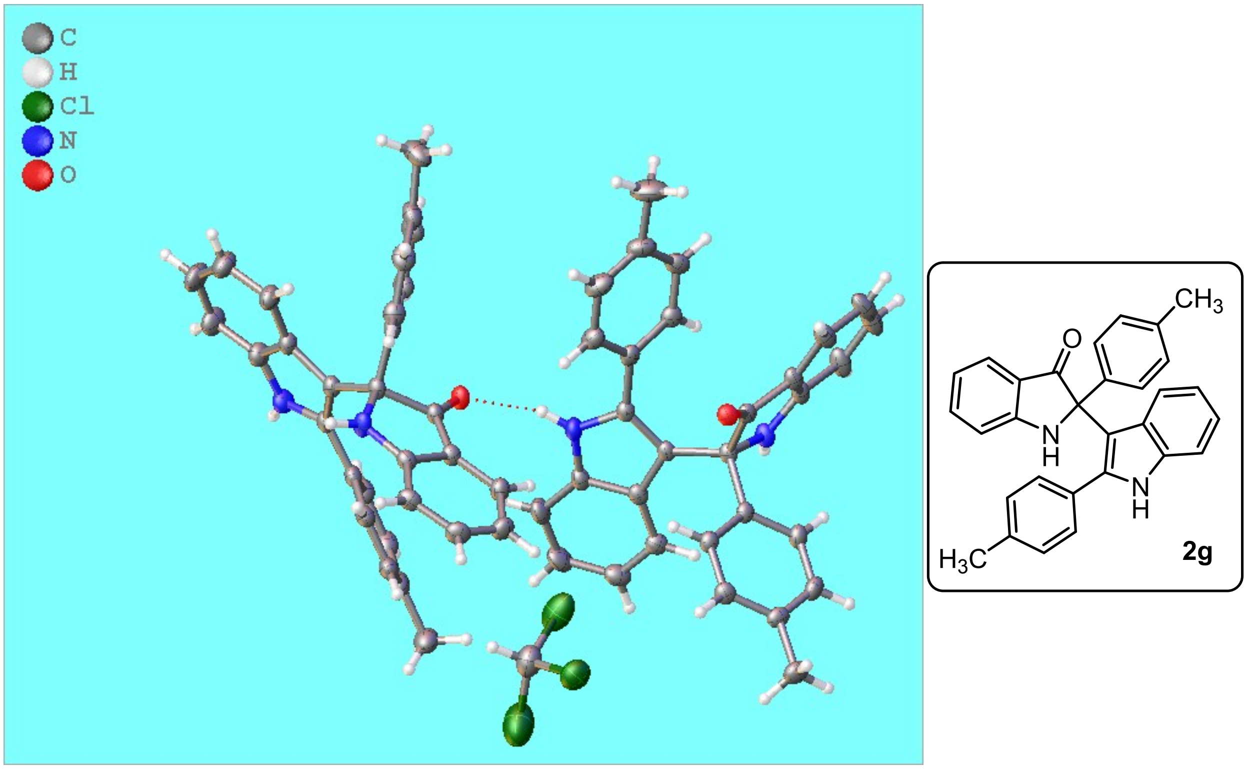

FigureS2 Single crystal X-ray of **2g(CCDC No.1832862)**. The thermal ellipsoids are drawn at the 50% probability level. The compound was crystalized with $CHCl_3$.

References:

1. Dolomanov, O.V., Bourhis, L.J., Gildea, R.J, Howard, J.A.K. &Puschmann, H. (2009), J. Appl. Cryst. 42, 339-341.
2. Sheldrick, G.M. (2015). ActaCryst. A71, 3-8.
3. Sheldrick, G.M. (2015). ActaCryst. C71, 3-8.
